# Comparison of efficacy of non-pharmacological intervention for post-stroke dysphagia: a systematic review and Bayesian network meta-analysis

**DOI:** 10.1186/s12868-023-00825-0

**Published:** 2023-10-16

**Authors:** Hao Zhu, Xinyuan Deng, Guorui Luan, Yu Zhang, Yichen Wu

**Affiliations:** 1 The First Clinical Medical College, Anhui University of Chinese Medicine, Hefei, China; 2 Anhui Integrated Traditional Chinese and Western Medicine Hospital, Hefei, China; 3 Tuina Department, The First Affiliated Hospital of Anhui University of Chinese Medicine, Hefei, China

**Keywords:** Post-stroke dysphagia, Non-pharmacological intervention, Effectiveness, Bayesian network meta-analysis

## Abstract

Increasingly, non-pharmacological interventions are being identified and applied to post-stroke dysphagia. Nevertheless, there is insufficient evidence to assess which type of interventions are more effective. In this study, the randomized controlled trials of non-pharmacological interventions on post-stroke dysphagia were retrieved from the relevant databases. Including 96 studies and 12 non-drug treatments. Then, and the network meta-analysis is carried out by statistical software. The results show: In the aspects of videofluoroscopic swallowing study (VFSS), Standardized Swallowing Assessment (SSA), swallowing-quality of life (SWAL-QOL), Water swallow test (WST); Acupuncture + electrotherapy + rehabilitation training, acupuncture + rehabilitation training + massage, electrotherapy + rehabilitation training, acupuncture + electrotherapy + rehabilitation training, electrotherapy, acupuncture + rehabilitation training + acupoints sticking application have significant effects in post-stroke dysphagia. Compared with other interventions, they have more advantages in improving the above indicators. A substantial number of high-quality randomized clinical trials are still necessary in the prospective to validate the therapeutic effectiveness of non-pharmacological interventions in post-stroke dysphagia and the results of this Bayesian network meta-analysis.

**Supplementary Information:**

The online version contains supplementary material available at 10.1186/s12868-023-00825-0.

## Introduction

Stroke was one of the diseases with a particularly high morbidity and mortality rate in the world [1]. The third national survey of causes of death in China revealed that stroke was the first. The World Stroke Organization's Global Stroke Facts 2022 indicated that stroke is the second leading cause of death in the world, leading to an increased economic burden, globally concentrated in low- and middle-income countries [2]. Post-stroke dysphagia is among the most common complications of stroke, with an prevalence of approximately 37–78% [3, 4]. The occurrence of post-stroke dysphagia is associated with dysfunction of certain organs (e.g., lips, tongue, pharynx, etc.) caused by cortical damage to the swallowing cortex, cortical medullary damage, and damage to the medullary swallowing center [5], which severely affects the patient's ingestion. Studies have shown that compared with patients with dysphagia caused by non-stroke, the respiratory function of post-stroke dysphagia patients is significantly decreased, and it is related to the severity of dysphagia [6]. This may increase the risk of pulmonary infections and aspiration pneumonia [7], which increased patient mortality by 11–16% [8–12]. Dysphagia was a risk factor for post-stroke secondary stroke [13], and the poor quality of life and high treatment costs had caused great burden to families and the country [14].

Currently, in addition to pharmacological treatment, which improves swallowing speed to some extent but has side effects and is very limited for post-stroke dysphagia, more non-pharmacological treatments are being discovered and applied. Promoting the recovery of swallowing function is the focus of the treatment of this disease. Guidelines [15–17] recommend non-pharmacological treatments including rehabilitation, Neuromuscular Electrical Stimulation (NMES), balloon dilation, acupuncture, and tui-na. An evaluation study by Bath PM et al. [18] showed that non-pharmacological treatments (incorporating interventions such as acupuncture, rehabilitation training, NMES, PES, transcranial direct current stimulation (tDCS), Transcranial Magnetic Stimulation (TMS)) were effective in reducing the length of stay and the incidence of pulmonary infections in patients with post-stroke dysphagia. A systematic evaluation by Lu et al. [19] showed that there is sufficient evidence to support that acupuncture can promote recovery of swallowing function in patients with post-stroke dysphagia. A META analysis by Liao et al. [20] noted that repetitive transcranial magnetic stimulation (rTMS) improved swallowing function in patients with dysphagia and that high-frequency repetitive transcranial magnetic stimulation may be more effective. The results of an RCT [21] showed that NMES could become an aggressive treatment modality for post-stroke dysphagia. These non-pharmacological treatments are safe, effective and have been widely used in the clinic, becoming a routine choice for clinicians and patients. Unfortunately, there are no studies that have systematically compared different types of nonpharmacologic treatments. Therefore, a comparison of the efficacy of non-pharmacological treatments for post-stroke dysphagia is warranted.

Network Meta is an indirect statistical analysis method. When encountering situations where there is no directly comparable primary studies, or where there are directly comparable primary studies but their quantity or quality is unsatisfactory; The main function of network meta is to comprehensively evaluate various interventions in the same evidence body at the same time [22].

In this study, RCTs of non-pharmacological treatment of post-stroke dysphagia from 2000 to the present were retrieved for a net meta-analysis, aiming to provide a reference for how to choose the optimal option for clinical treatment of this disease.

## Methods

### Search method

We conducted a comprehensive search of PubMed, EMBASE, Cochrane Library, CINAHL, SinoMed, VIP Database for Chinese Technical Periodicals (VIP), WANFANG DATA and CNKI for the period January 2000 to October 2022. See the Additional file 1 for specific search strategies.

Two researchers (HZ,XD) selected articles that met the inclusion and exclusion criteria, imported all articles into EndNote X8 (Clarivate, Philadelphia, PA, USA) to filtered out duplicate articles, and obtained the results after screening the titles and abstracts. When the article selection is uncertain, the third party shall participate in the negotiation and finally decide whether to include it.

### Inclusion criteria

#### ①Subject

- Adults aged 18–90 years who meet the diagnostic criteria for post-stroke dysphagia;
- No significant intellectual and consciousness impairment; no local lesions of the pharynx such as thyroid disorders, local infections, ulcers, etc.; no serious complications of vital organs (heart, lung, liver, kidney).

#### ②Intervention

The interventions in the treatment and control groups in this review were non-pharmacological including: acupuncture, manipulation and other stimulation point therapy, surface neuromuscular electrical stimulation (e.g., tDCS, transcranial magnetic stimulation and surface neuromuscular electrical stimulation), rehabilitation training (e.g., tongue and jaw resistance training, tongue training and swallowing training, and routine care). The treatment and control groups could not be different methods of the same intervention. There are no restrictions on the frequency and duration of treatment.

#### ③Outcome indicators

Primary outcome indicators included VFSS (The videofluoroscopic swallowing study) [23], SSA (Standardized Swallowing Assessment) [24]; secondary outcome indicators included SWAL-QOL (swallowing-quality of life) [25], WST (water swallow test) [26].

The scale of VFSS is 0–10, and the higher scores, the better swallowing function. The scores range of SSA is 17–46, and the lower scores, the better swallowing function. The score range of SWAL-QOL is 44–220, and the higher scores, the better quality of life. The score range of WST is 1–5, and the lower scores, the better swallowing function.

#### ④Study design

Articles that used a randomized controlled design and had access to the full text and complete study data.

### Quality assessment

Quality assessment was performed by two independent researchers using the Cochrane Collaboration Risk of Bias tool [27]. The Cochrane Collaboration Risk of Bias tool consists of seven items: random sequence generation, allocation concealment, blinding of participants and personnel, blinding of outcome assessment, incomplete outcome data, selective reporting, and other biases. Each item is divided into low risk, high risk or unclear risk. The overall risk of study bias was defined as “low” when all of the above items were rated as “low risk”, “high” when one or more of the seven items were rated as “high risk”, and "unclear" in all other cases. When differences arise, they should be resolved through discussion or negotiation with a third-party researcher.

### Data extraction

Two researchers independently extracted baseline information and outcome data into tabular form, including the first author, publication year, disease duration, sample size, participant characteristics (age and gender), interventions measures, adverse events and outcomes data.

### Data synthesis and statistical analysis

Review manager 5.3 (Cochrane Library, London, UK) software was used for literature quality evaluation. The network structure diagram was drawn using Stata 17.0 (StataCorp LLC,USA), ADDIS v1.16.8 (IMI Get Real Initiative,EU) and Gemtc-gui-0.14.3 (IMI Get Real Initiative,EU) software for network analysis.

Nodes in the network structure diagram indicate different interventions, and nodes and connecting lines are weighted according to the number of studies containing directly compared interventions. Larger nodes indicate more occurrences in the corresponding direct comparisons, and thicker connecting lines indicate a higher number of corresponding two-by-two direct comparisons.

The node-splitting analysis method should be used to test the local inconsistency. If P > 0.05, it shows that direct comparison and indirect comparison are very consistent. When the direct evidence and indirect evidence are inconsistent, refer to the direct comparison result.

The 4 Markov chains are used to set the initial values, the initial value of the model is 2.5, the iteration step size is fine-tuned by 10, the number of iterations is adjusted by 20,000, and the number of simulated iterations is 50,000. After the software parameters are set, the convergence of the iterative effect is judged by the potential scale reduced factor (PSRF), and when the value of PSRF is close to or equal to 1 (1 ≤ PSRF ≤ 1.05), the convergence is complete, indicating that the model is stable and the data can be analyzed.

For the outcome indicators, all the outcome indicators included in this study were continuous variables and were expressed as effect values and their 95% confidence interval credibility interval (CI). The data in the cells under the interventions represent the MD values and 95% Cl values of the efficacy between the interventions with this corresponding column and the row interventions. When 95%Cl contains 0 indicates that the results are not significant, and when 95%Cl does not contain 0 indicates that the results are significant. MD < 1 means that row intervention is superior to column intervention, and vice versa. The interventions were then analyzed according to a probability ranking table to know the strengths and weaknesses of each intervention. See Additional file 2 for specific software operation procedures and guidelines.

## Results

### Search results

**Fig. 1:**
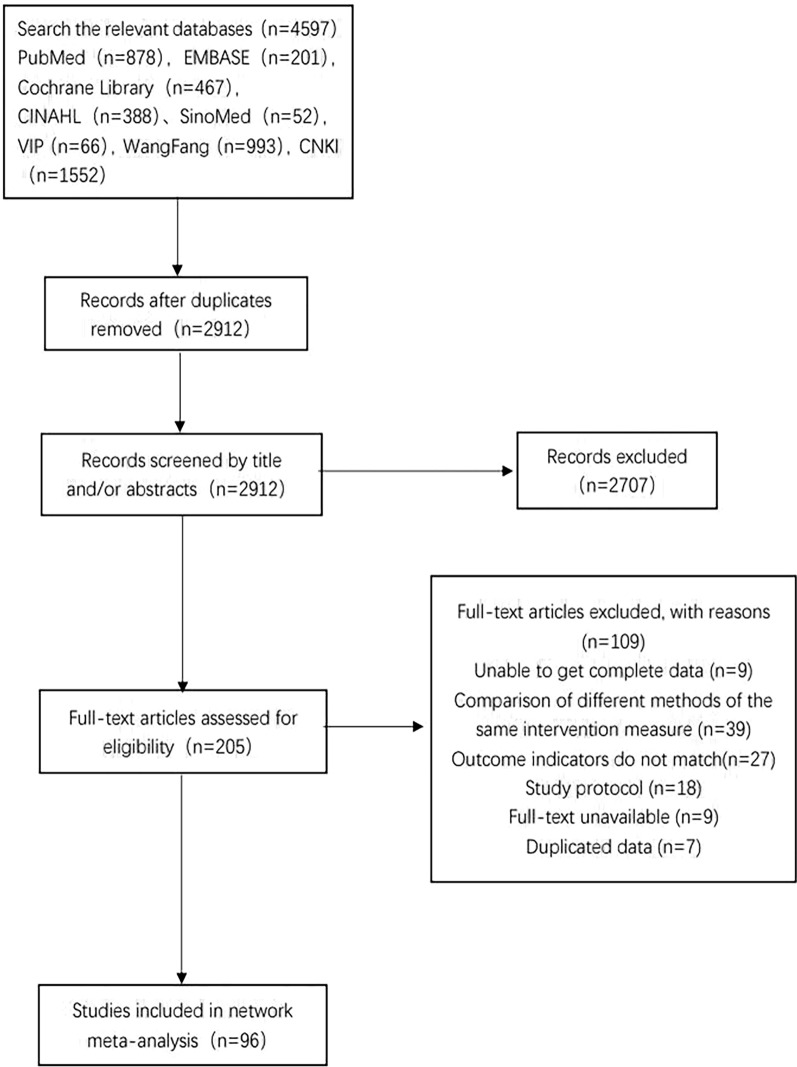
Screening flow diagram

A systematic search of seven electronic databases produced 4,594 potentially relevant records. After removing 1682 duplicates, the title and abstract of each record were screened, and 2709 records that did not meet the inclusion criteria were excluded. The full text of the remaining 203 records was retrieved to further assess their eligibility, and 109 studies were excluded based on the reasons listed in Fig. 1. Ultimately, 96 studies were included that met the criteria for the net meta-analysis. Figure 1 showed the search process.

### Quality evaluation of the included studies

**Fig. 2:**
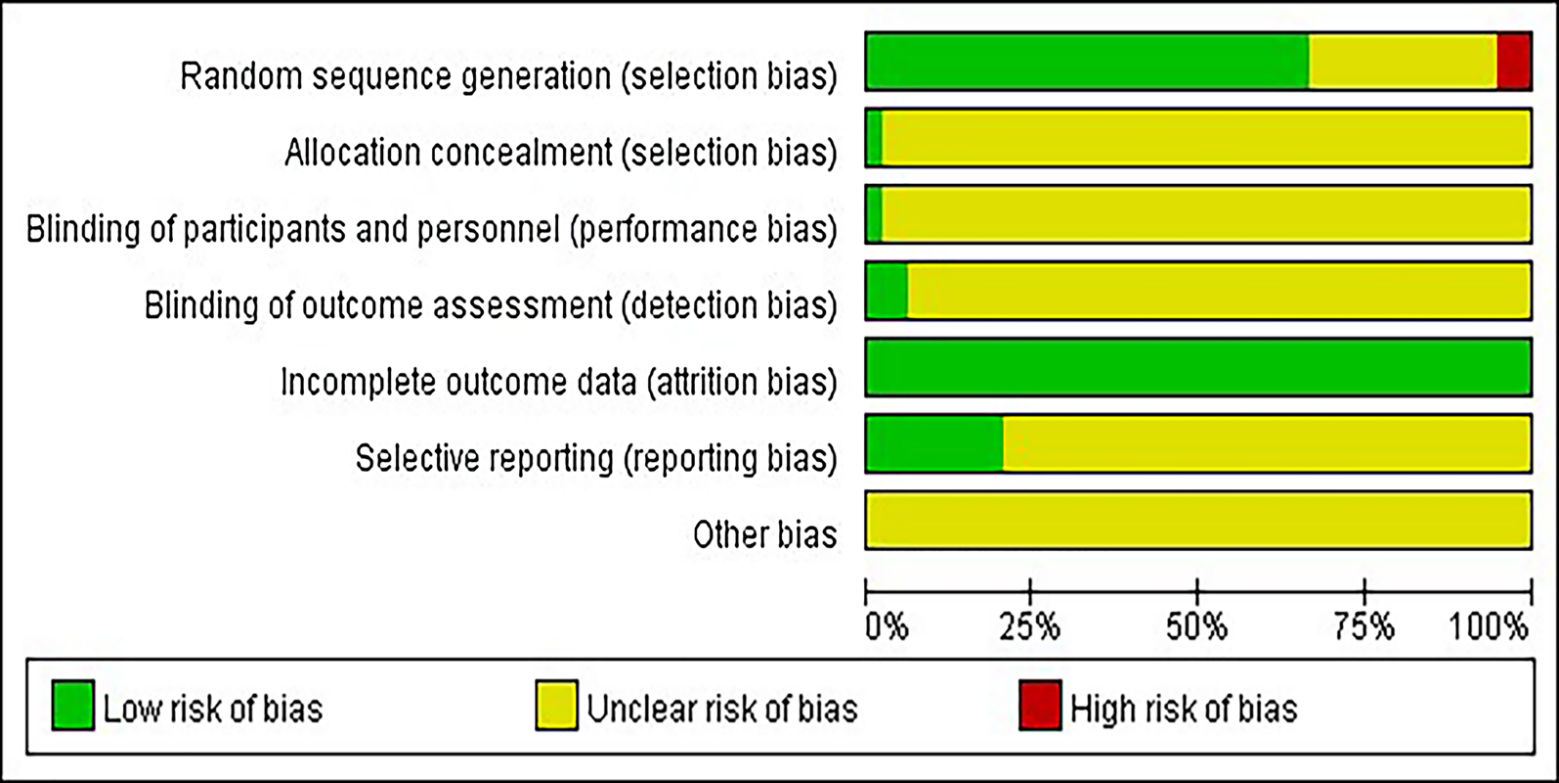
Risk of bias in inclusion literature

A total of 96 articles were included in the quality assessment of the included literature. 65 of the 96 included articles mentioned the specific randomization method (58 of them used random number table method and 7 used computer software randomization method) and 28 articles mentioned the word random but did not mention the specific randomization method. Regarding blinding, 6 of the 96 articles mentioned blinding of data outcome statisticians, and 3 articles mentioned blinding of participants. Among the included articles, 20 articles mentioned case shedding, dropouts, and lost visits. The results are plotted in Fig. 2 using RevMan 5.3 software. Table 1 shows the characteristics of each study included in our network meta-analysis, and details of the specific included literature are shown in Additional file 3. For documentation purposes, the 12 non-pharmacological interventions covered by the included literature were defined as follows: A:acupuncture B:electrotherapy C: rehabilitation training D: conventional treatment E: acupuncture + electrotherapy F: acupuncture + rehabilitation training G: electrotherapy + rehabilitation training H: acupuncture + electrotherapy + rehabilitation training I: acupoints sticking J: acupuncture + rehabilitation training + massage K: rehabilitation training + acupoints sticking L: acupuncture + rehabilitation training + acupoints sticking. The included literature interventions all included conventional treatment.

**Table 1:** Table of characteristics of included literature

Intervention	Proportion
A	9 (4.43%)
B	5 (2.46%)
C	71 (34.98%)
D	8 (3.94%)
E	6 (2.96%)
F	51 (25.12%)
G	28 (13.79%)
H	17 (8.37%)
I	2 (0.99%)
J	1 (0.49%)
K	2 (0.99%)
L	3 (1.48%)
Sample size	
Treatment group	4201 (60.92%)
Control group	2694 (39.08%)
Gender	
Male	2604 (58.98%)
Female	1811 (41.02%)
Average duration of treatment	
Less than or equal to 30 days	75 (78.13%)
More than 30 days	21 (21.87%)
Outcome indicators	
①	36 (25%)
②	39 (27.08%)
③	27 (18.75%)
④	42 (29.17%)

①VFSS

②SSA

③SWAL

④WST

### Qualitative and meta-analysis of two-by-two comparisons of interventions

Due to the limited space of the article, see the Additional file 4 for details.

### Efficacy and ranking probability of non-pharmacological interventions obtained by Bayesian network meta-analysis

#### VFSS score

**Fig. 3:**
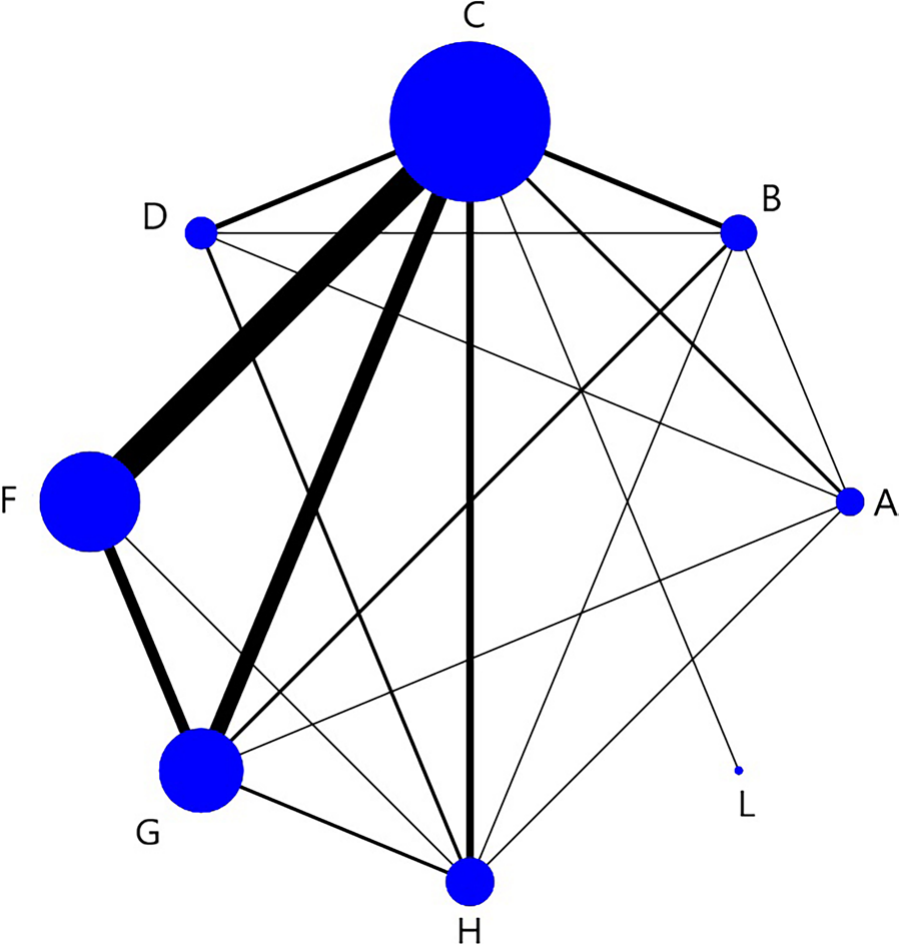
VFSS network structure diagram. The node size represents the sample size, and the connecting line between nodes represents the number of RCT. The same below

Figure 3 showed a network structure diagram of the VFSS score, indicating that eight types of non-pharmacological interventions provided data, with the most common comparison being between C and F.

Table 2 showed the relative impact of different interventions on VFSS. Interpretation of results: F, G, H > A, B, C, D and K > D and C > D (Symbol > indicates that the former has more advantages than the latter in improving indicators). There were no significant differences in the comparison of the remaining methods.

**Table 2:** Relative effects of different interventions on the VFSS

	A	B	C	D	F	G	H	K
A	0							
B	0.02 (−1.70, 1.79)	0						
C	0.08 (−1.34, 1.48)	0.05 (−1.23, 1.28)	0					
D	1.53 (−0.18, 3.24)	1.50 (−0.14, 3.14)	**1.45 (0.19, 2.72)**	0				
F	−**1.70 (**−**3.20, **−**0.22)**	−**1.73 (**−**3.08, **−**0.40)**	−**1.77 (**−**2.36, **−**1.20)**	−**3.23 (**−**4.60, **−**1.89)**	0			
G	−**1.60 (**−**3.01, **−**0.15)**	−**1.62 (**−**2.96, **−**0.36)**	−**1.67 (**−**2.37, **−**0.95)**	−**3.12 (**−**4.50, **−**1.76)**	0.10 (−0.67, 0.87)	0		
H	−**2.44 (**−**4.04, **−**0.88)**	−**2.48 (**−**3.92, **−**1.03)**	−**2.51 (**−**3.48, **−**1.54)**	−**3.97 (**−**5.31, **−**2.63)**	−0.74 (−1.80, 0.32)	−0.85 (−1.87, 0.16)	0	
K	−1.38 (−4.30, 1.50)	−1.41 (−4.31, 1.43)	−1.45 (−4.03, 1.07)	−**2.92 (**−**5.78, **−**0.12)**	0.32 (−2.34, 2.92)	0.23 (−2.46, 2.84)	1.07 (−1.67, 3.75)	0

Bold emphasis means that the results are statistically significant

**Fig. 4:**
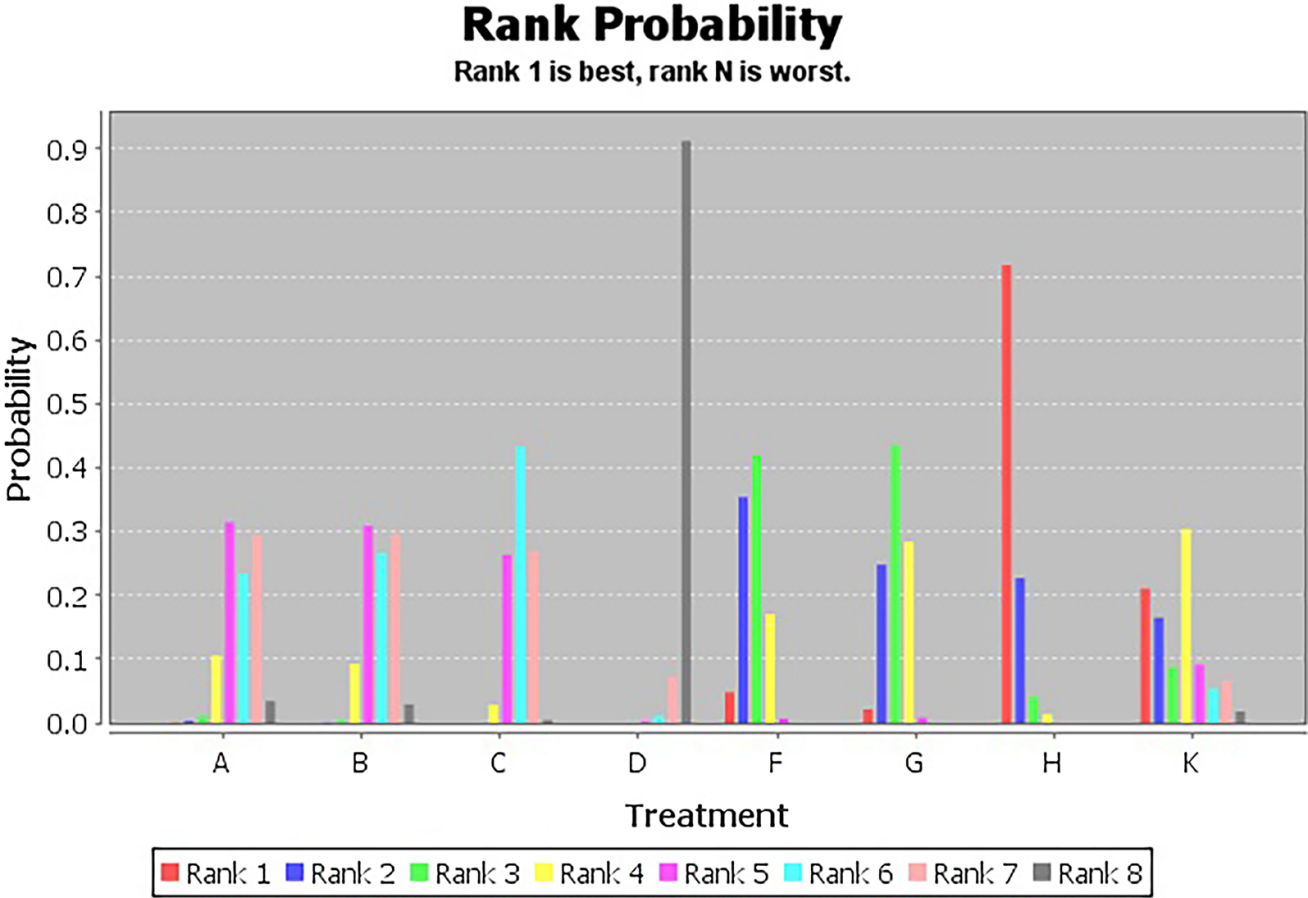
VFSS probability diagram

Figure 4 showed the ranking probabilities of different non-pharmacological interventions on VFSS. The results showed that the probability ranking was H (72%) > K (21%) > F (5%) > G (2%), suggesting that H was the most likely to be the best intervention.

#### SSA

**Fig. 5:**
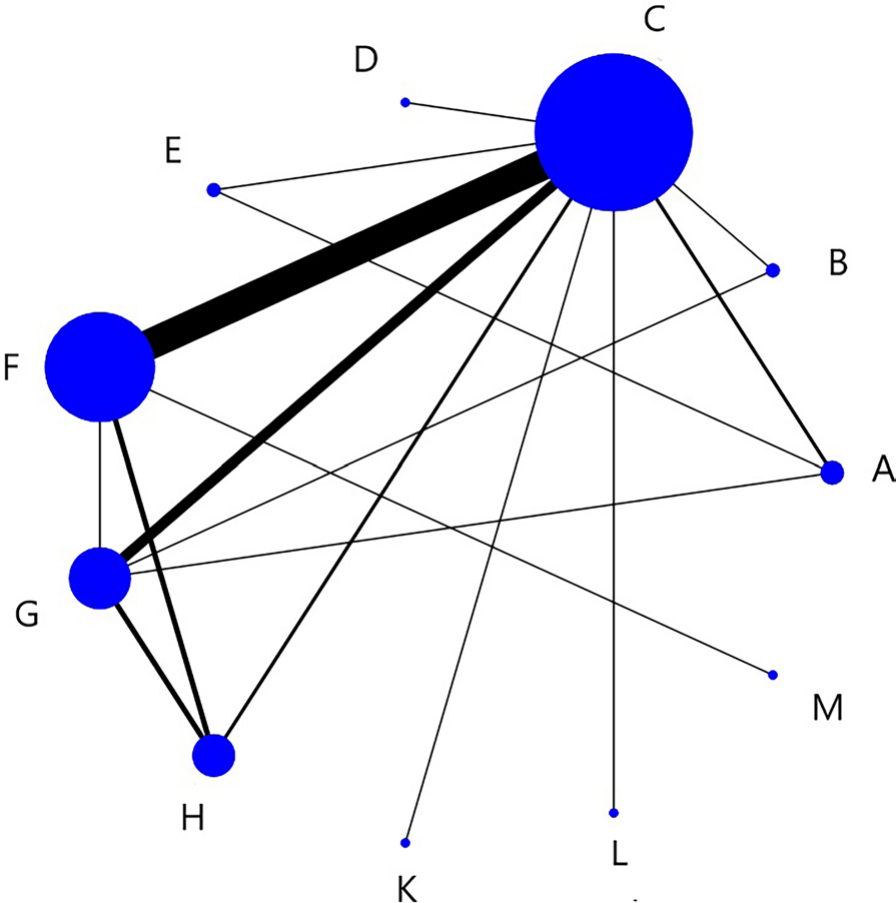
SSA network structure diagram

Figure 5 showed a network structure diagram of the SSA, indicating that 11 types of non-pharmacological interventions provided data, with the most common comparison being between C and F. Table 3 showed the relative impact of different interventions on SSA. The results showed that H > A and F, G, H > B and E, F, G, H, J, K > D and E, F, G, H > C (Symbol > indicates that the former has more advantages than the latter in improving indicators). The remaining methods were not significantly different from each other.

**Table 3:** Relative effects of different interventions on the SSA

	A	B	C	D	E	F
A	0					
B	−3.13 (−9.67, 3.28)	0				
C	−1.26 (−4.65, 2.23)	1.90 (−3.51, 7.50)	0			
D	−6.39 (−13.41, 0.82)	−3.22 (−11.42, 5.28)	−5.16 (−11.31, 1.15)	0		
E	4.12 (−0.72, 8.85)	7.26 (−0.05, 14.74)	**5.32 (0.40, 10.30)**	**10.52 (2.50, 18.30)**	0	
F	2.47 (−1.21, 6.23)	**5.60 (0.07, 11.34)**	**3.69 (2.22, 5.13)**	**8.86 (2.35, 15.18)**	−1.63 (−6.79, 3.49)	0
G	2.68 (−1.01, 6.44)	**5.82 (0.45, 11.42)**	**3.88 (1.82, 5.92)**	**9.04 (2.32, 15.52)**	−1.42 (−6.58, 3.66)	0.20 (−2.09, 2.53)
H	**4.77 (0.60, 8.92)**	**7.89 (2.00, 13.91)**	**5.97 (3.47, 8.53)**	**11.14 (4.33, 17.70)**	0.66 (−4.88, 5.98)	2.27 (−0.36, 4.91)
J	5.44 (−2.11, 12.94)	8.56 (−0.05, 17.34)	6.63 (−0.04, 13.30)	**11.77 (2.56, 20.85)**	1.36 (−7.08, 9.52)	2.94 (−3.45, 9.39)
K	4.46 (−2.83, 11.39)	7.56 (−0.67, 15.96)	5.63 (−0.49, 11.97)	**10.76 (2.01, 19.42)**	0.34 (−7.57, 8.21)	1.95 (−4.34, 8.40)
L	0.70 (−6.34, 7.74)	3.87 (−4.37, 12.10)	1.90 (−4.35, 8.01)	7.07 (−1.91, 15.56)	−3.44 (−11.34, 4.52)	−1.80 (−8.21, 4.48)

G	H	J	K	L
0				
2.07 (−0.58, 4.73)	0			
2.74 (−4.23, 9.62)	0.69 (−6.34, 7.67)	0		
1.75 (−4.78, 8.34)	−0.35 (−7.07, 6.51)	−0.90 (−10.20, 7.98)	0	
−1.97 (−8.47, 4.39)	−4.07 (−10.77, 2.53)	−4.80 (−13.78, 4.35)	−3.71 (−12.55, 5.10)	0

Bold emphasis means that the results are statistically significant

**Fig. 6:**
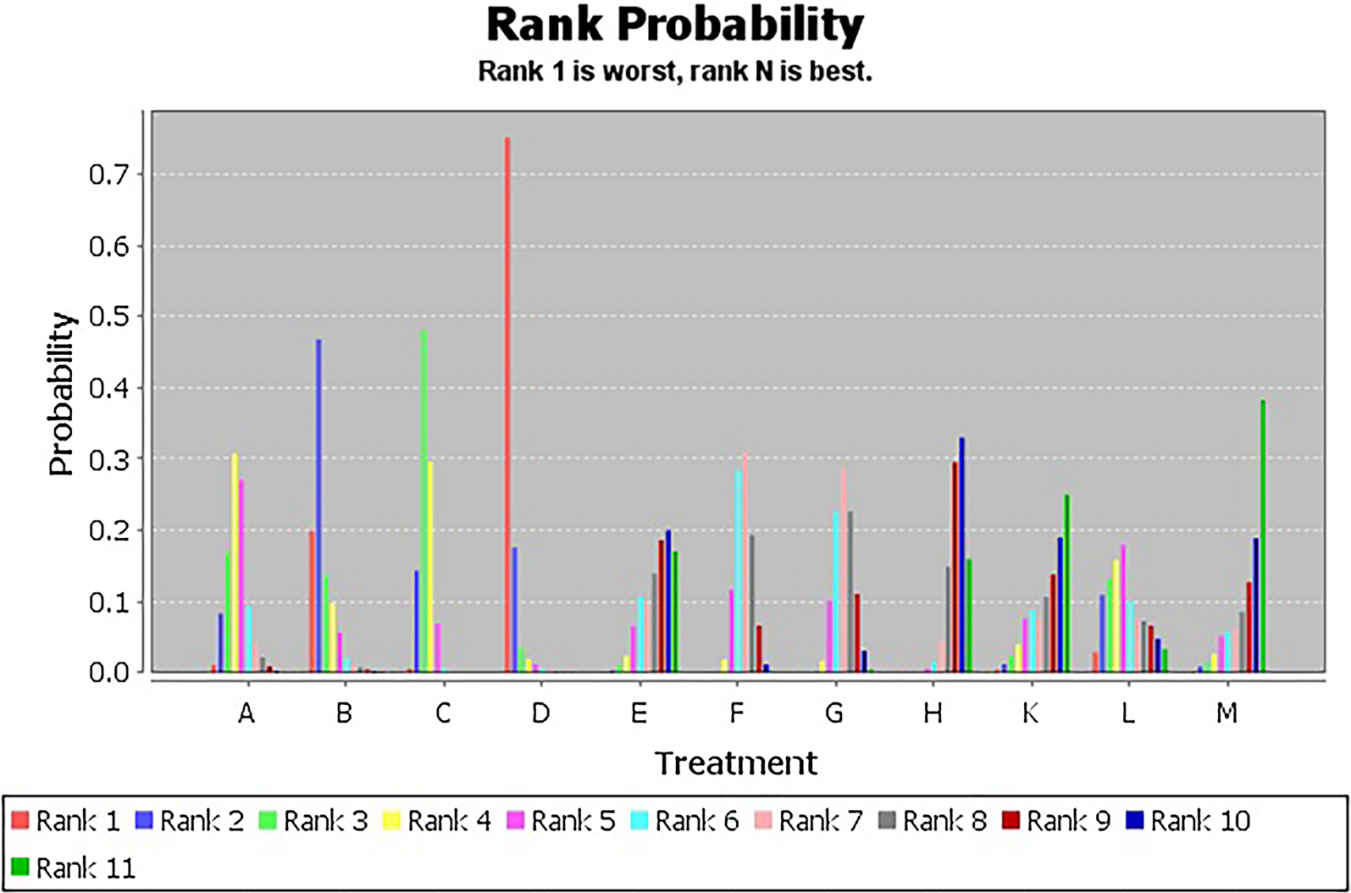
SSA probability diagram

Figure 6 showed the ranking probabilities of different non-pharmacological interventions on SSA. The results showed that the probability ranking was J (39%) > K (25%) > E (17%) > H (15%) > L (3%), suggesting that J was the most likely to be the best intervention.

#### SWAL

**Fig. 7:**
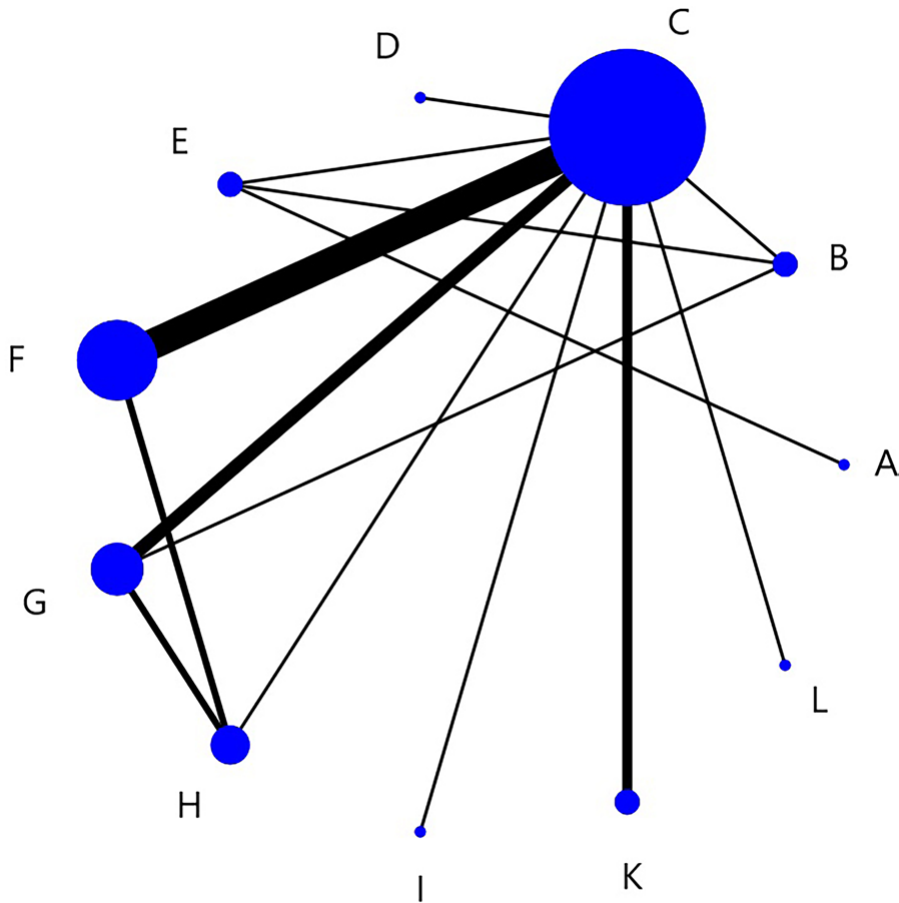
SWAL network structure diagram

Figure 7 showed a network structure diagram of the SWAL, indicating that 11 types of non-pharmacological interventions provided data, with the most common comparison being between C and F. Table 4 showed the relative impact of different interventions on SWAL. The results showed that B > A,C, E,F,G and C,F,H,L > G(Symbol > indicates that the former has more advantages than the latter in improving indicators). There were no significant differences in the comparison of the remaining methods.

**Table 4:** Relative effects of different interventions on the SWAL

	A	B	C	D	E	F
A	0					
B	−**119.42 (**−**233.98, **−**4.82)**	0				
C	−25.18 (−139.50, 89.84)	**94.18 (26.04, 161.61)**	0			
D	−13.92 (−158.95, 131.11)	105.72 (−5.15, 218.21)	11.26 (−75.07, 99.48)	0		
E	−26.08 (−111.41, 63.99)	**93.09 (23.35, 164.65)**	−0.53 (−72.25, 72.91)	−11.70 (−126.09, 102.12)	0	
F	−43.43 (−161.54, 74.41)	**75.66 (2.22, 146.62)**	−18.61 (−47.19, 9.46)	−30.27 (−121.54, 61.74)	−17.47 (−96.03, 58.38)	0
G	21.20 (−95.43, 139.08)	**141.42 (69.34, 211.20)**	**46.81 (8.63, 86.53)**	35.66 (−60.78, 132.60)	47.51 (−31.53, 126.34)	**65.46 (20.75, 111.92)**
H	−43.62 (−165.68, 79.01)	75.72 (−4.90, 152.83)	−18.71 (−63.49, 26.52)	−30.16 (−128.86, 69.43)	−17.57 (−101.35, 65.67)	−0.28 (−44.59, 45.08)
I	−50.95 (−196.56, 94.17)	68.42 (−44.80, 179.80)	−25.73 (−113.54, 62.93)	−37.27 (−161.60, 85.54)	−24.81 (−141.11, 88.61)	−7.12 (−100.62, 85.25)
K	−43.13 (−187.78, 102.70)	76.75 (−37.22, 189.59)	−18.04 (−107.50, 70.78)	−29.95 (−155.67, 95.58)	−17.63 (−135.13, 96.05)	0.59 (−93.62, 95.11)
L	−49.01 (−174.02, 75.53)	70.85 (−15.24, 156.09)	−23.45 (−75.84, 27.18)	−35.19 (−137.01, 65.23)	−22.65 (−113.21, 63.57)	−5.09 (−64.24, 53.49)

G	H	I	K	L
0				
−**65.89 (**−**113.07, **−**18.78)**	0			
−72.45 (−171.04, 21.00)	−7.42 (−106.90, 92.07)	0		
−64.96 (−162.57, 31.34)	0.65 (−98.88, 100.23)	8.09 (−119.68, 132.46)	0	
−**70.51 (**−**135.35, **−**6.86)**	−4.57 (−73.72, 61.94)	2.44 (−100.66, 103.91)	−5.54 (−106.07, 99.56)	0

Bold emphasis means that the results are statistically significant

**Fig. 8:**
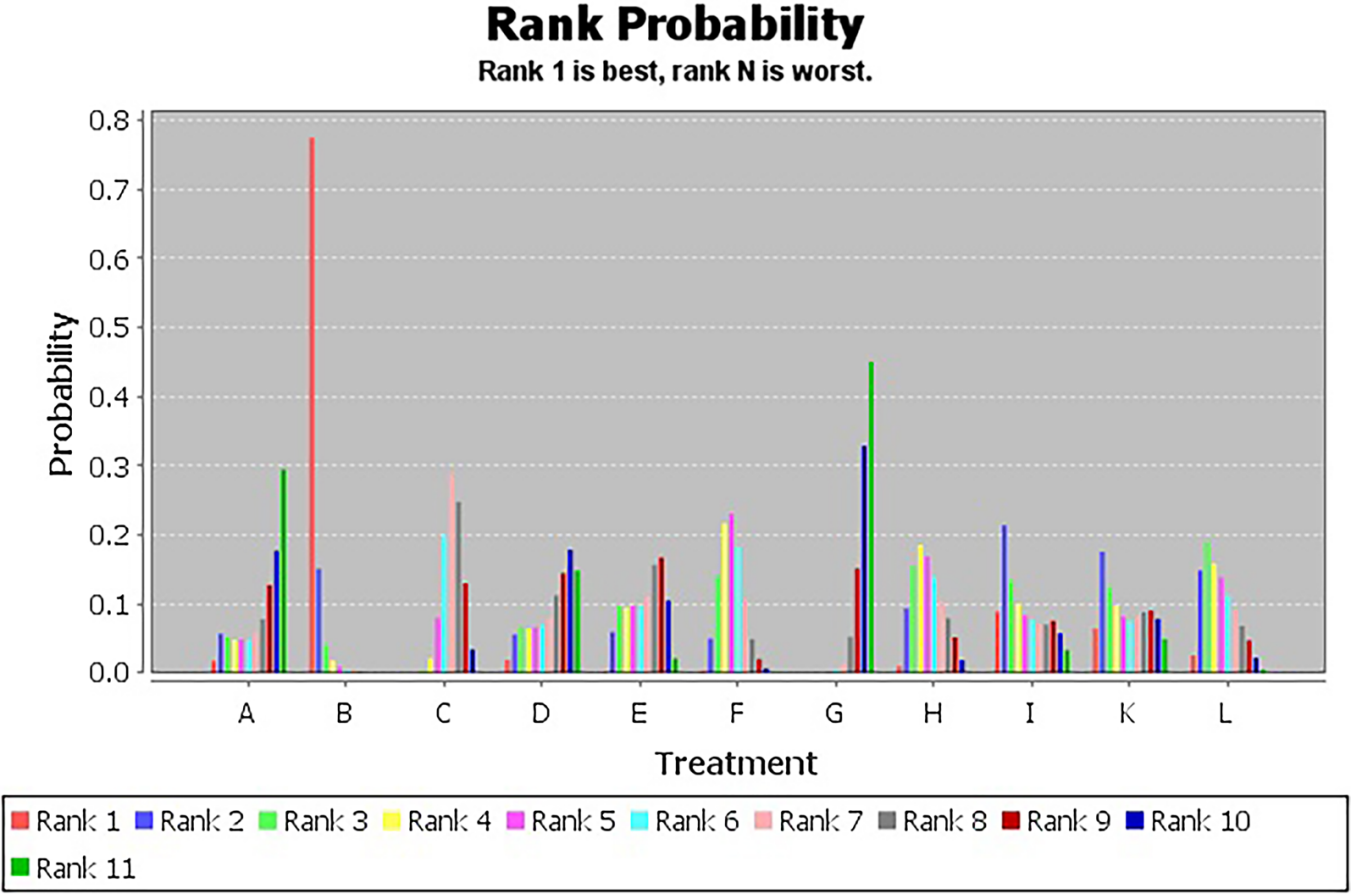
SWAL probability diagram

Figure 8 showed the ranking probabilities of different non-pharmacological interventions on SWAL. The results showed that the probability ranking was B (77%) > I (9%) > K (6%) > A (2%) = D (2%) = L (2%) = H (1%), suggesting that G was the most likely to be the best intervention.

#### WST

**Fig. 9:**
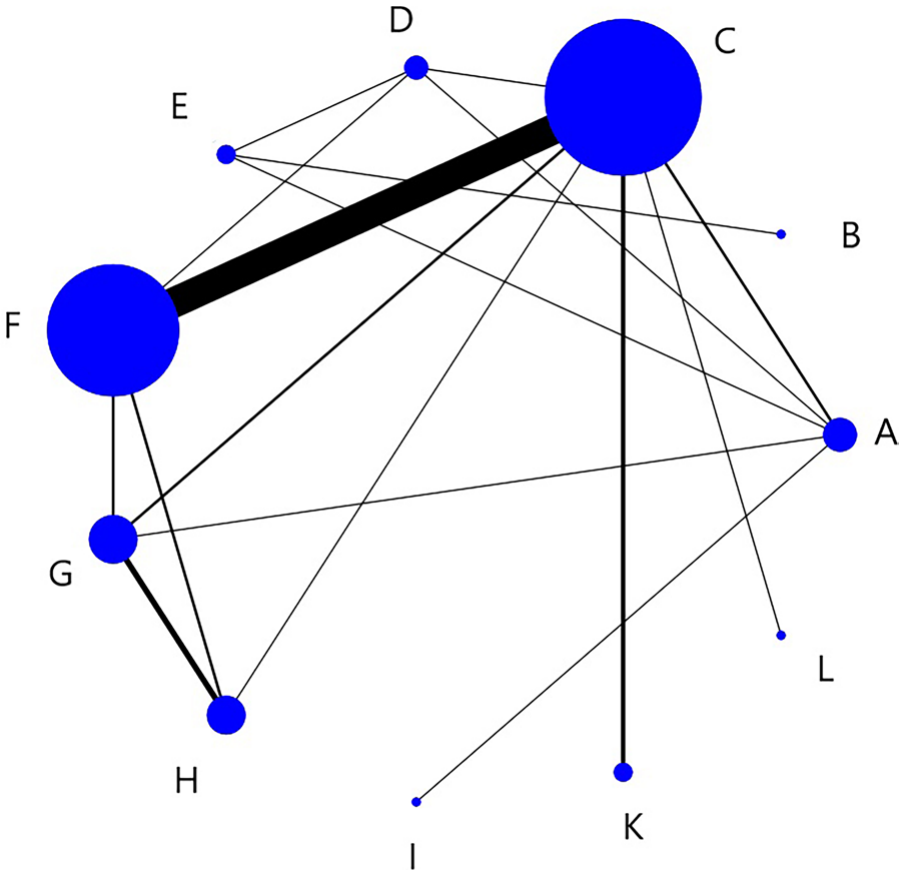
WST network structure diagram

Figure 9 showed a network structure diagram of WST, indicating that 11 types of non-pharmacological interventions provided data, with the most common comparison being between C and F.

Table 5 shows the relative impact of different interventions on WST. The results showed that L > A, B, C, D, F, G, H and F > C (Symbol > indicates that the former has more advantages than the latter in improving indicators). There were no significant differences in the comparison of the remaining methods.

**Table 5:** Relative effects of different interventions on the WST

	A	B	C	D	E	F
A	0					
B	−1.03 (−4.52, 2.48)	0				
C	−0.33 (−1.78, 1.16)	0.70 (−2.92, 4.29)	0			
D	−1.12 (−2.79, 0.60)	−0.08 (−3.55, 3.39)	−0.78 (−2.42, 0.85)	0		
E	0.31 (−1.81, 2.47)	1.35 (−1.40, 4.12)	0.64 (−1.71, 3.05)	1.43 (−0.67, 3.57)	0	
F	0.55 (−0.95, 2.09)	1.57 (−2.05, 5.20)	**0.88 (0.30, 1.46)**	1.67 (−0.02, 3.32)	0.24 (−-2.19, 2.63)	0
G	0.59 (−1.09, 2.26)	1.62 (−2.10, 5.34)	0.91 (−0.29, 2.10)	1.70 (−0.21, 3.62)	0.27 (−2.27, 2.80)	0.03 (−1.19, 1.28)
H	0.48 (−1.41, 2.37)	1.50 (−2.31, 5.30)	0.81 (−0.58, 2.17)	1.58 (−0.48, 3.66)	0.16 (−2.51, 2.81)	−0.07 (−1.46, 1.31)
I	1.00 (−1.82, 3.74)	2.05 (−2.46, 6.44)	1.32 (−1.82, 4.48)	2.10 (−1.14, 5.34)	0.67 (−2.89, 4.16)	0.44 (−2.73, 3.60)
K	0.33 (−2.82, 3.45)	1.36 (−3.28, 5.90)	0.65 (−2.09, 3.41)	1.43 (−1.77, 4.69)	0.01 (−3.65, 3.68)	−0.23 (−3.03, 2.62)
L	**3.02 (0.88, 5.28)**	**4.05 (0.11, 8.01)**	**3.35 (1.75, 5.02)**	**4.13 (1.87, 6.47)**	2.71 (−0.12, 5.60)	**2.47 (0.76, 4.25)**

G	H	I	K	L
0				
−0.11 (−1.37, 1.13)	0			
0.40 (−2.85, 3.67)	0.52 (−2.88, 3.90)	0		
−0.25 (−3.25, 2.72)	−0.13 (−3.23, 2.95)	−0.65 (−4.81, 3.51)	0	
**2.44 (0.43, 4.51)**	**2.54 (0.45, 4.75)**	2.03 (−1.49, 5.63)	2.69 (−0.52, 5.98)	0

Bold emphasis means that the results are statistically significant

**Fig. 10:**
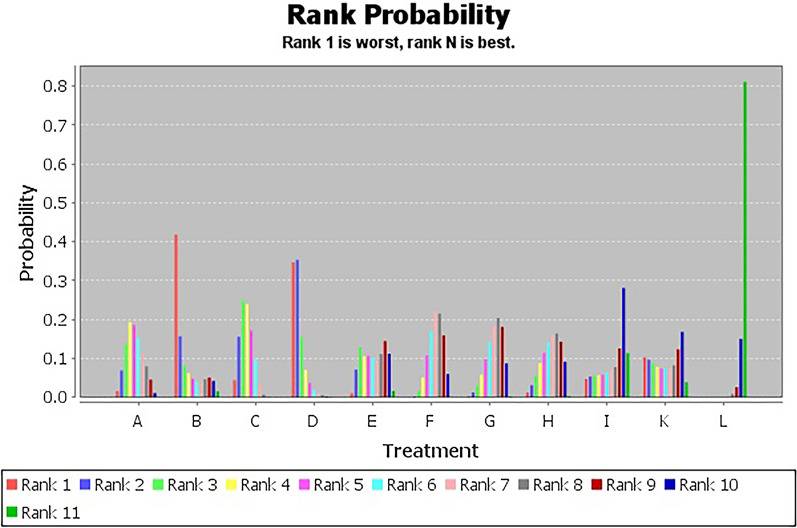
WST probability diagram

Figure 10 shows the ranking probabilities of different non-pharmacological interventions on WST. The results showed that the probability ranking was L (81%) > I (11%) > K (4%) > B (2%) = E (2%), suggesting that L was the most likely to be the best intervention.

### Consistency check

The network meta analysis of VFSS and WST shows that the consistency is > 0.05, which is good. The network meta of SSA is analyzed by node-splitting, and FH nodes have local inconsistency; The network meta of SSA is analyzed by node-splitting, and there is local inconsistency between BC and BG nodes. See the Additional file 5 for details.

### Subgroup analysis and sensitivity analysis

In order to explore the source of inconsistency, SSA and SWAL were analyzed in subgroups according to age, course of treatment and sample size. The results show that age and course of treatment may be the possible reasons for the local inconsistency of SSA network meta. According to sensitivity analysis, excluding the three studies included in FH, the results showed that the probability ranking was J (49%) > K (26%) > E (19%) > l (3%), suggesting that J was the most vital to be the best intervention. The node-splitting analysis showed that the consistency was > 0.05. It is basically consistent with the probability ranking of the original network meta intervention measures. See the Additional file 5 for details.

The results show that age, course of treatment and sample size may be the possible reasons for the local inconsistency of SWAL network meta. According to the difference of average age, the subgroup analysis shows that when the average age is ≤ 65 years old, the results showed that the probability ranking was H (54%) > G (17%) > I (15%), and the node-splitting analysis shows that the consistency is > 0,05, which is good. When the average age > 65 years old, the results shown that the probability ranking was B (68%) > F (16%) > A (6%) > H (5%), suggesting that B was the most vital to be the best intervention. The node-splitting analysis shows that the consistency is > 0.05, and the consistency is good. See the Additional file 5 for details.

## Discussion

Non-pharmacological interventions have been found to be clinically effective in improving post-stroke dysphagia, but the clinical efficacy of different non-pharmacological therapies varies. A total of 96 RCT articles involving 12 different non-pharmacological interventions for post-stroke dysphagia were included in the article, and their efficacy was compared in detail using a network Meta-analysis.

The results of the reticulated meta-analysis showed that in terms of VFSS, the literature involved eight therapies A, B, C, D, F, G, H, and K. H have more advantages in improving VFSS. And H has the highest probability ranking with 72%. Similar results have been reported in previous systematic reviews [28]. Acupuncture modulates the cortex of the brainstem reticular formation and the swallowing center, thereby controlling the swallowing reflex and coordinating the movements of swallowing-related muscles; Acupuncture increases mitochondrial peroxidase, enhances cellular metabolism, promotes neurotransmitter transmission, and repairs damaged brain tissue [29]. Neuromuscular electrical stimulation can directly stimulate pharyngeal muscles with a certain frequency and intensity, induce the swallowing reflex, enhance the muscle strength of the swallowing muscles and re-establish the cortical control function; And improve local tissue blood circulation, enhance the flexibility and coordination of pharyngeal muscles, prevent muscle wasting atrophy, achieve the purpose of improving and restoring swallowing function [30]. Rehabilitation training can improve the flexibility and coordination of oral muscles and promote the recovery of swallowing function [31]. Our results indicated that the combination of rehabilitation training with acupuncture and electrical stimulation was more effective than monotherapy.

The results of the network meta-analysis in terms of SSA showed that the literature involved 11 therapies A, B, C, D, E, F, G, H, J, K, and L. J have a probability ranking of 38%. This showed that acupuncture + rehabilitation training + massage has a significant effect in improving SSA. Tui Na treats post-stroke dysphagia by promoting local muscle function recovery and improving the function of the face, throat and larynx [32]. Our results suggested that the combination of acupuncture + rehabilitation training + massage was the most effective in improving SSA.

The results of the network meta-analysis in terms of SWAL showed that the literature involved 11 therapies A, B, C, D, E, F, G, H, I, K, and L. Compared with other interventions, B have more advantages in improving the WST. And the probability ranking was 77%. When the average age is ≤ 65 years old, H have more advantages. When the average age is > 65 years old, B have more advantages.In summary, B and H were the most clinically effective in improving SWAL, electrotherapy and the combination of acupuncture + electrotherapy + rehabilitation training have more advantages.

In addition, the results of the network meta-analysis showed that in terms of WST, the literature involved 11 therapies A, B, C, D, E, F, G, H, I, K, and L. Compared with other interventions, L have more advantages in improving the WST. The probability ranking was 81%. By continuously stimulating the patient's meridians and acupuncture points, the acupoints sticking stimulates the patient's brainstem network structure, thus stimulating the nerve center and helping the patient to form poor swallowing reflexes and improve the patient's swallowing ability [33]. In general, the acupuncture + rehabilitation training + acupoints sticking combination have more advantages in improving WST.

This study comprehensively analyzed the efficacy of 12 different non-pharmacological interventions used in the treatment of post-stroke dysphagia. Only RCTs were included in our net meta-analysis, involving 96 articles. The results of this network meta-analysis could provide guidance for clinical selection of non-pharmacological interventions for post-stroke dysphagia.

However, this study still had some limitations: (1) The quality of some literatures included in the study were low. There were 28 literatures that mention the word "randomization", but no specific randomization method. Only 6 literatures mentioned the blind methods. (2) The inclusion of literature in English and Chinese languages only may result in the omission of a portion of literature in other languages. (3) The small amount of literature and sample size of some interventions may lead to the reduction of test efficacy.

## Conclusion

This network meta-analysis provided an exhaustive comparison of the efficacy of 12 non-pharmacological interventions for post-stroke dysphagia and concluded that: Non-pharmacological interventions, especially acupuncture + electrotherapy + rehabilitation training, acupuncture + rehabilitation training + massage, electrotherapy + rehabilitation training, acupuncture + electrotherapy + rehabilitation training, electrotherapy, acupuncture + rehabilitation training + acupoints sticking, are highly effective for post-stroke dysphagia. A large number of high-quality randomized clinical trials are still needed in the future to validate the treatment effectiveness of non-pharmacological interventions in post-stroke dysphagia and the results of this network meta-analysis.

### Supplementary Information

**Additional file 1. **Specific search strategies.

**Additional file 2. **Software operation.

**Additional file 3. **Characteristics of each study.

**Additional file 4.** Qualitative and meta-analysis of two-by-two comparisons of interventions.

**Additional file 5. **Consistency analysis and subgroup analysis.

## Data Availability

The datasets generated and analyzed during the current study are publicly available.
