# Supplementary material for: Comparison of efficacy of non-pharmacological intervention for post-stroke dysphagia: a systematic review and Bayesian network meta-analysis
Source: BMC Neurosci. 2023 Oct 16;24:53. doi: 10.1186/s12868-023-00825-0 (PMC10578008; doi:10.1186/s12868-023-00825-0)
Supplement: Supplementary file 1 — Additional file 1. Specific search strategies. [file 12868_2023_825_MOESM1_ESM.docx]

Appendix 1 Specific search strategies

Use the following four groups of title terms (MeSH terms or Emtree terms) and text words to screen the literature independently:

#1 stroke OR poststroke OR post-stroke OR cerebrovasc* OR 'brain vasc*' OR 'cerebral vasc*' OR cva* OR apoplex* OR 'ischemi* attack*' OR 'ischaemi* attack*' OR TIA OR TIAs OR 'neurologic* deficit*' OR SAH OR AVM；#2 Dysphagia OR dysphagia OR swallow OR swallowing OR deglutition OR speech OR voice OR language；#3 'transcranial neurostimulation' OR 'transcranial magnetic stimulation' OR TMS OR 'transcranial direct current stimulation' OR 'tDCS' OR 'sNMES' OR 'cortical stimulation' OR 'non-invasive brain stimulation' OR 'bioelectrical stimulation' OR 'medium pulse repetition frequency' OR rehabilitation OR acupuncture OR massage OR cupping OR 'point sticking' OR 'acupoint catgut embedding' OR 'tongue training' OR 'swallowing training' OR psychological OR 'motor imagery' OR 'blood-letting therapy' OR 'balloon dilation' OR 'speech therapy' OR 'proposal for a modified jaw opening exercise' OR 'hyperbaric oxygen therapy'；#4'Randomised controlled trial' OR 'controlled clinical trial' OR randomised OR placebo OR randomisation OR randomisation OR randomly OR trial OR groups；The title terms AND text wORds in each group are connected by "or", and the other three groups are searched by "and". The language is limited to English and Chinese.
