# Supplementary material for: Comparison of efficacy of non-pharmacological intervention for post-stroke dysphagia: a systematic review and Bayesian network meta-analysis
Source: BMC Neurosci. 2023 Oct 16;24:53. doi: 10.1186/s12868-023-00825-0 (PMC10578008; doi:10.1186/s12868-023-00825-0)

**Appendix 2-Software operation**

**ADDIS software related operations**

1. Double-click the software icon and click the "New Dataset" button in the interface that appears.


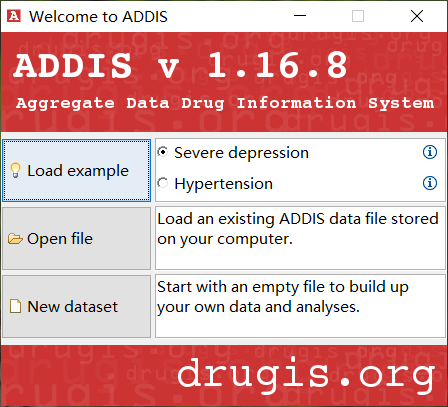
a

2. The "New File" interface appears.


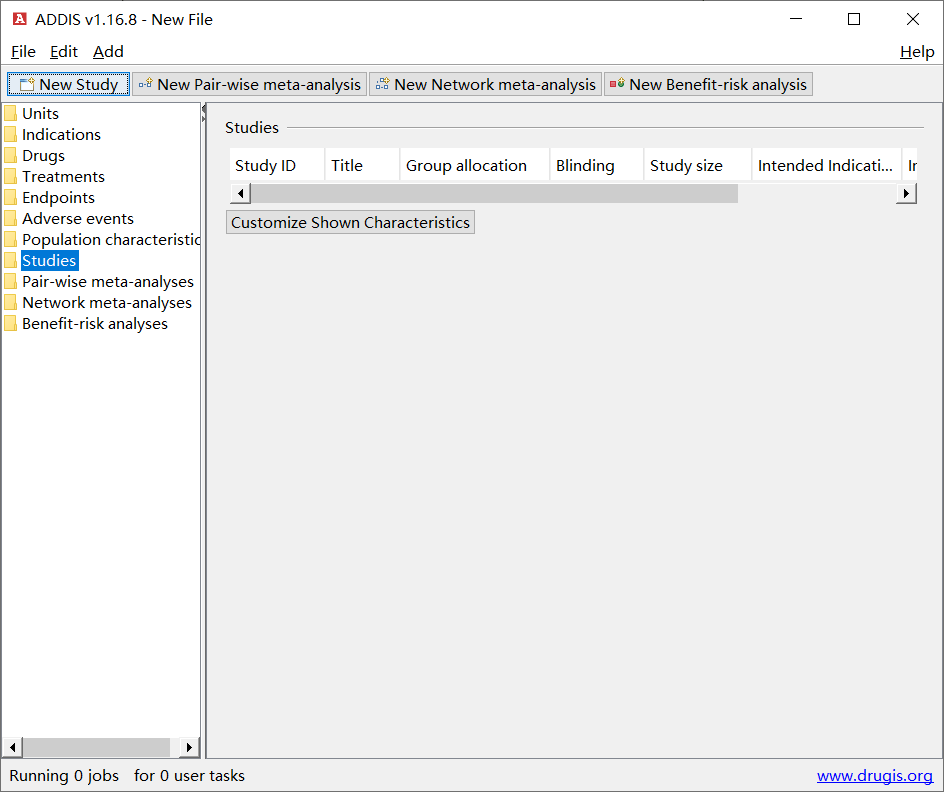


3. Add indications


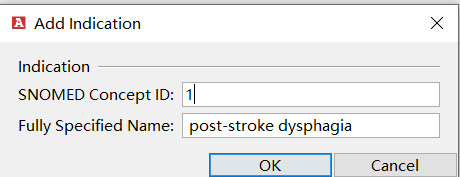


4. Add interventions, and add all 13 interventions in turn.


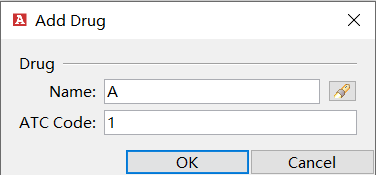


5. Add Endpoint, and add all Endpoints in turn.


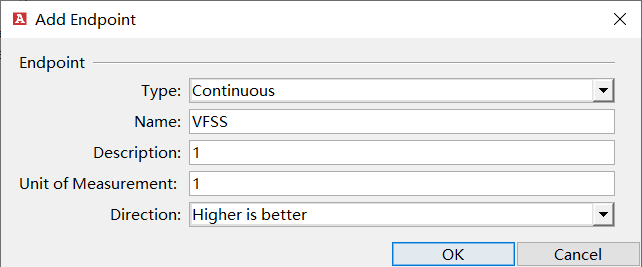


6. Add research and input data (the top left corner of the new research box is Add study, and the screenshot is the input of demonstration data, and some settings and operations are omitted, which are the same as those in the new interface and do not affect the actual operation process).

6.1 Enter the research group name into the ID box and the Title box.


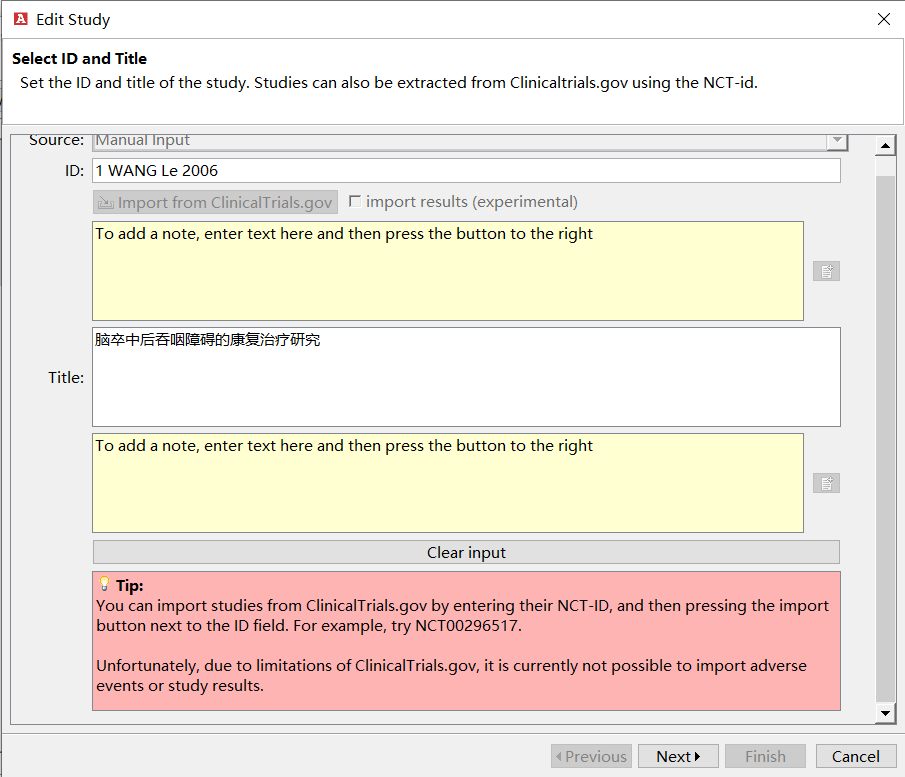


6.2 Select indications


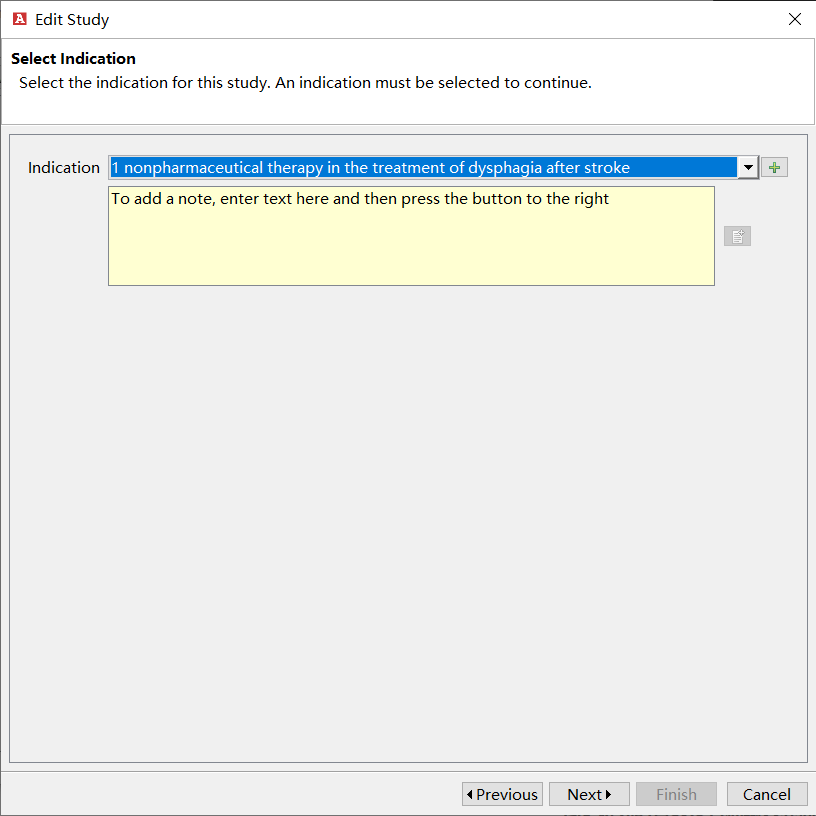


6.3Adds additional information.


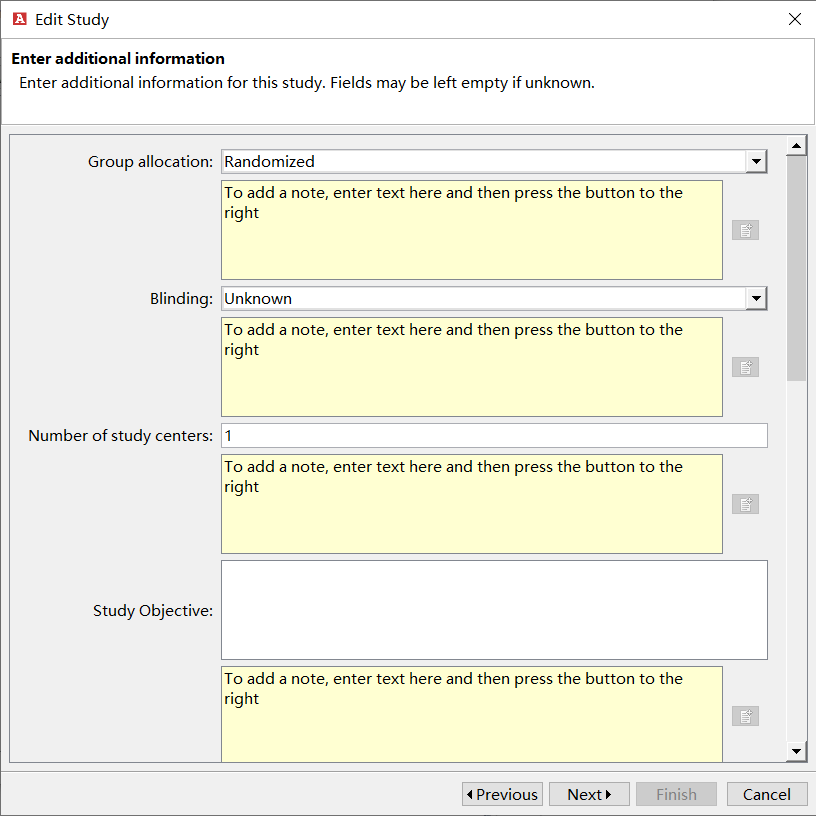


6.4 Add arms


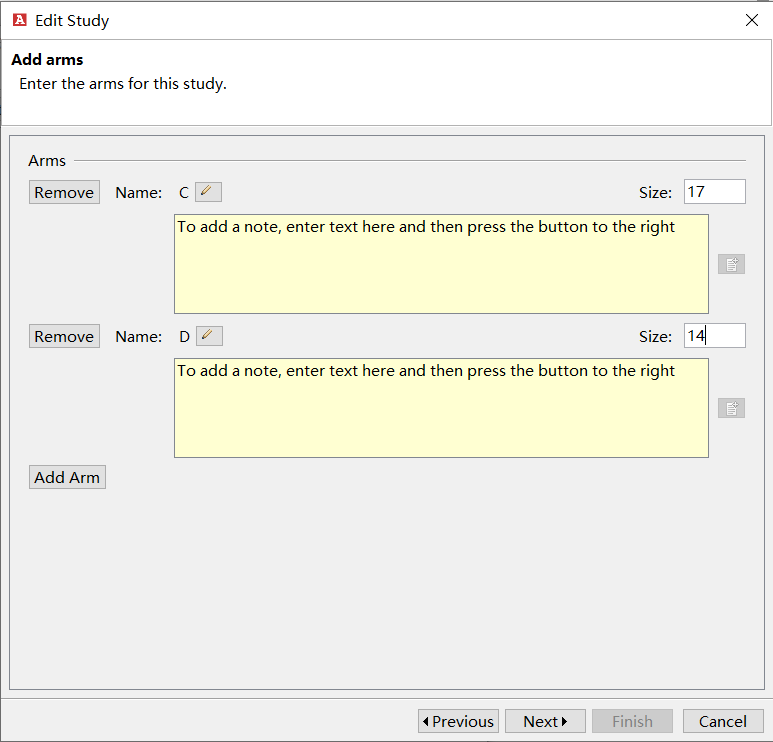


6.5 relevant information


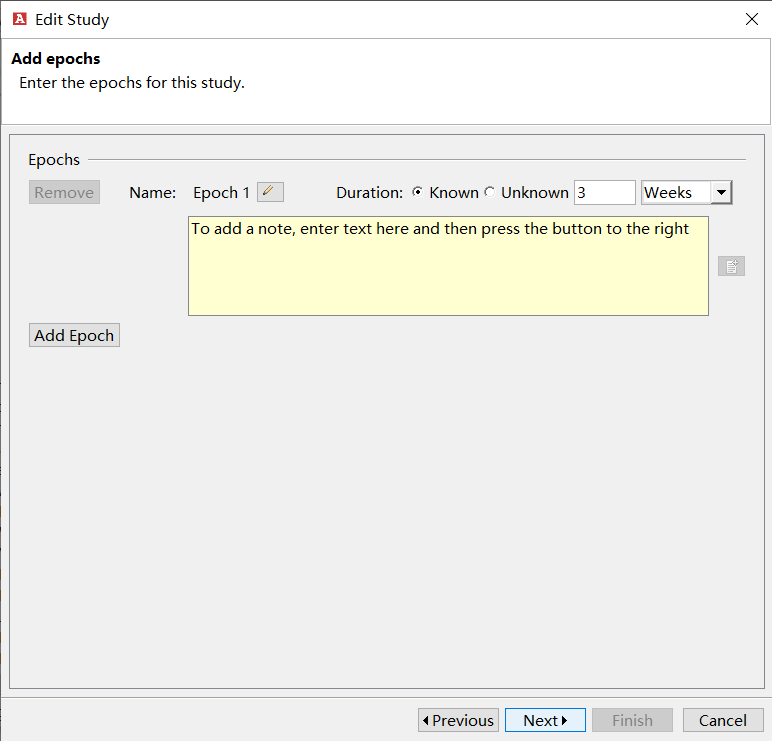


6.6 Outcome indicator data input


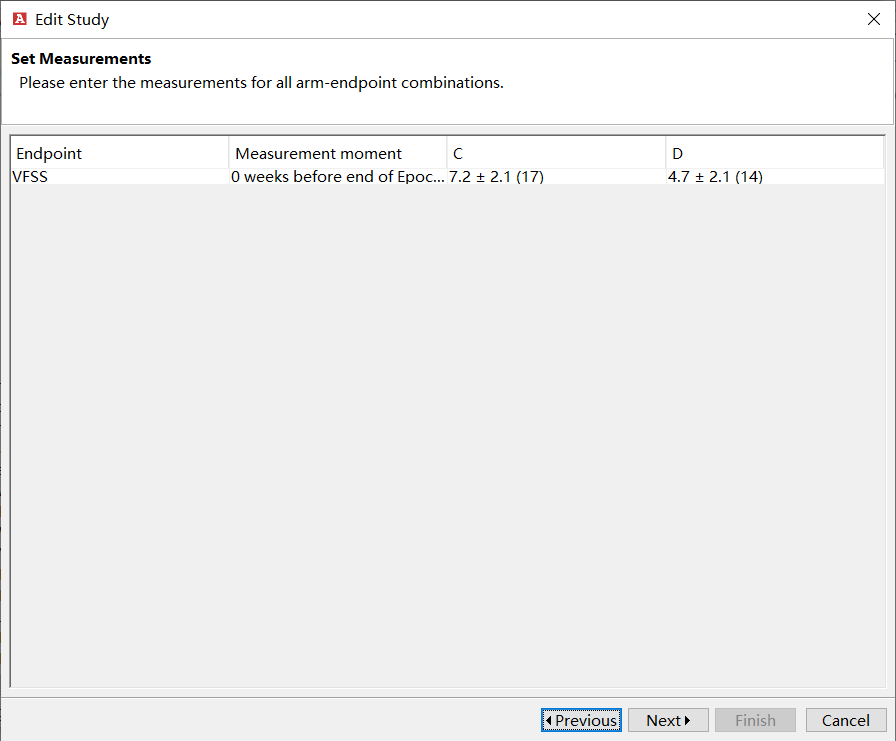


6.7 Click "Next" until the interface shown in Figure 9 appears, and click "Finish" to complete a set of data entry. Record all 98 groups of data in turn.


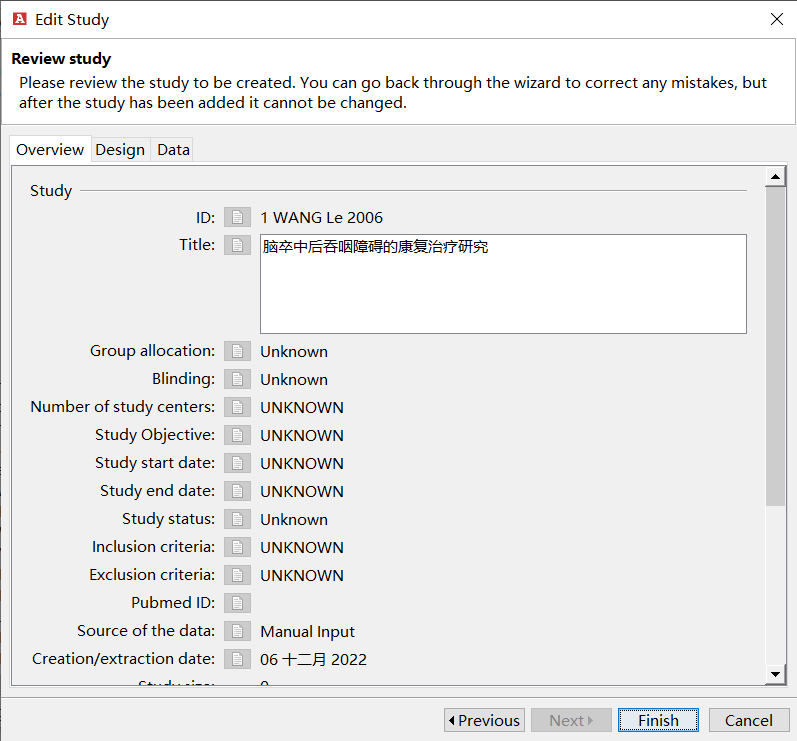


7. Network meta analysis

7.1 New Analysis


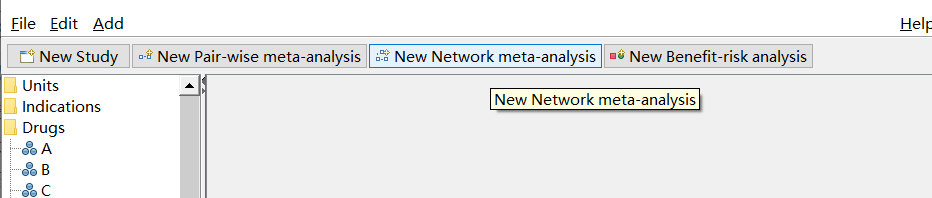


7.2 After selecting the ending indicator, keep clicking Next until the last interface appears and click Finsh to complete the operation.


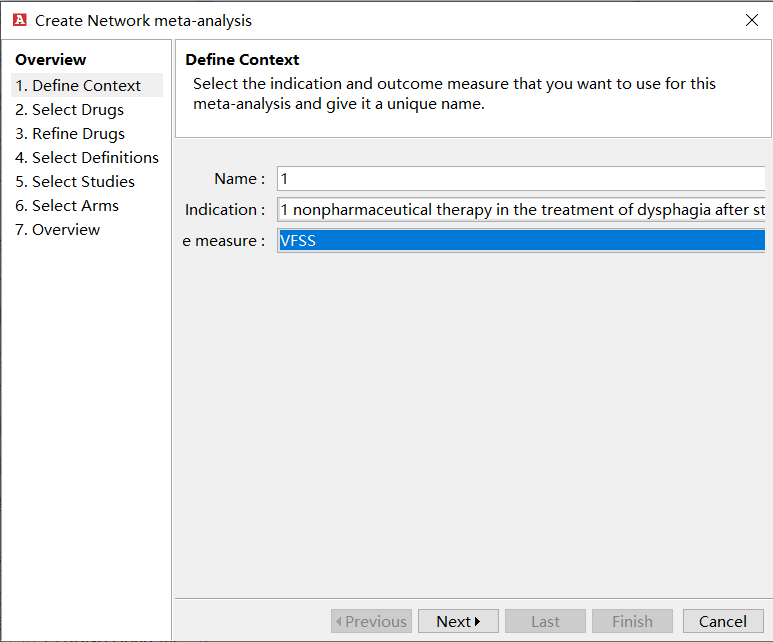


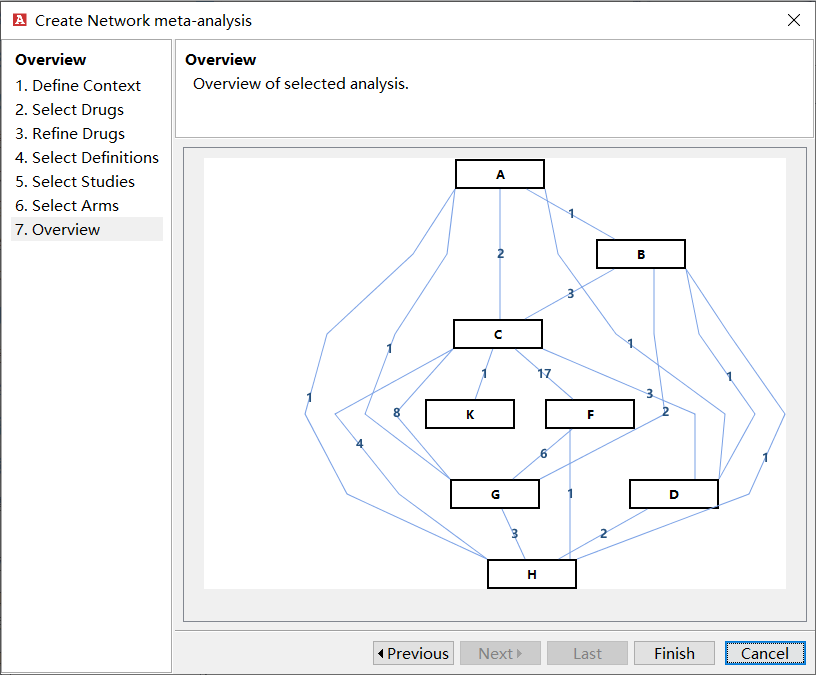


7.3 Consistency model (click the green button to get the result, and click the button to the right of the completion point to view convergence)


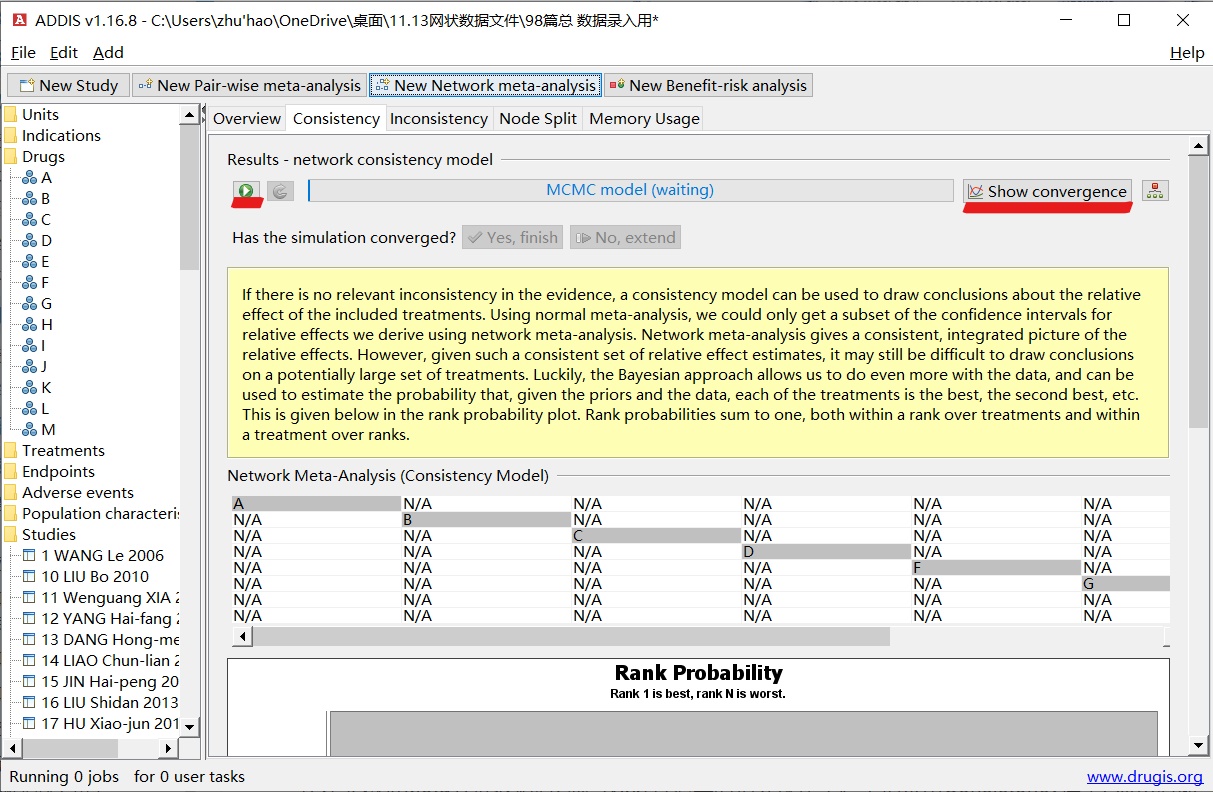


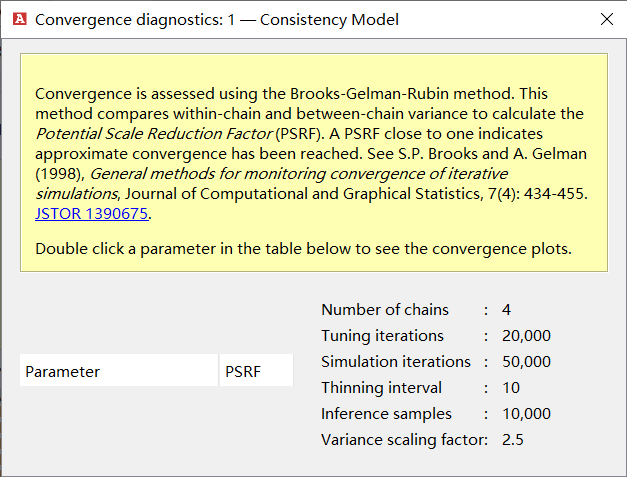


7.4 Node Split analysis (same as above)


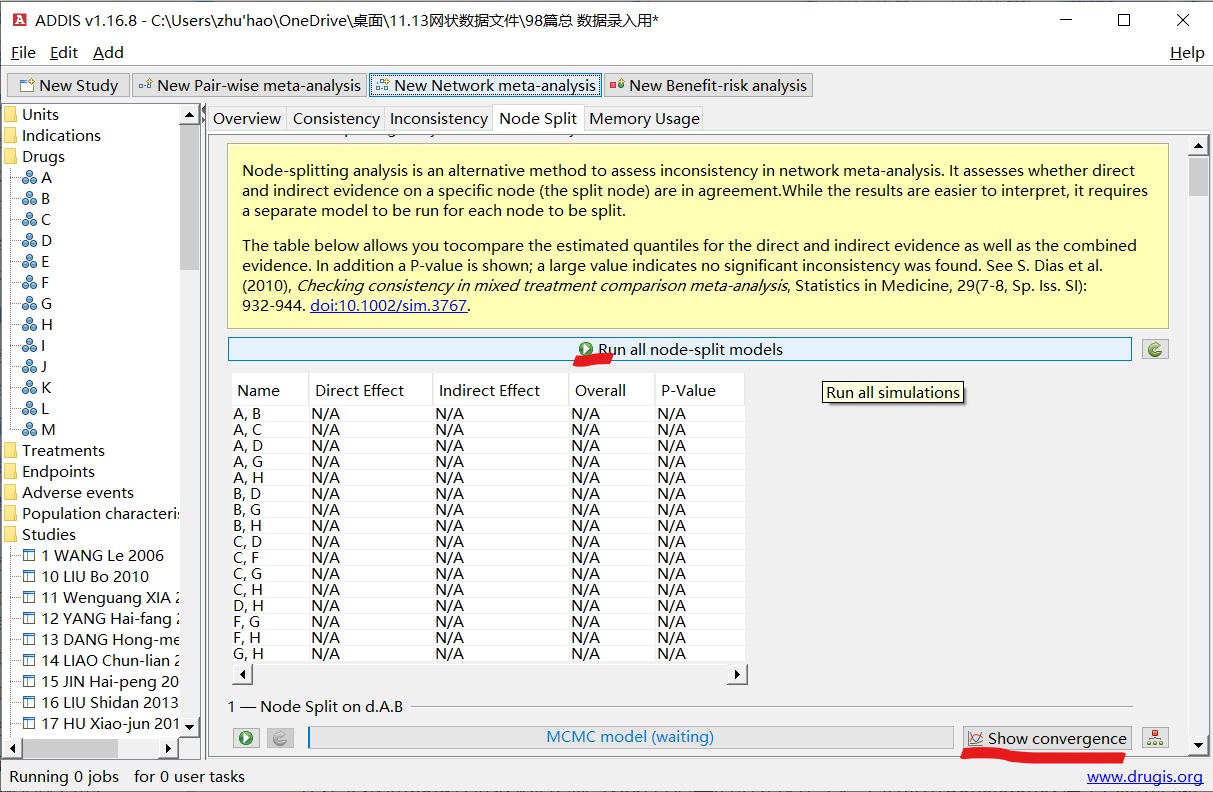


**Drawing figure of risk of bias with revman**


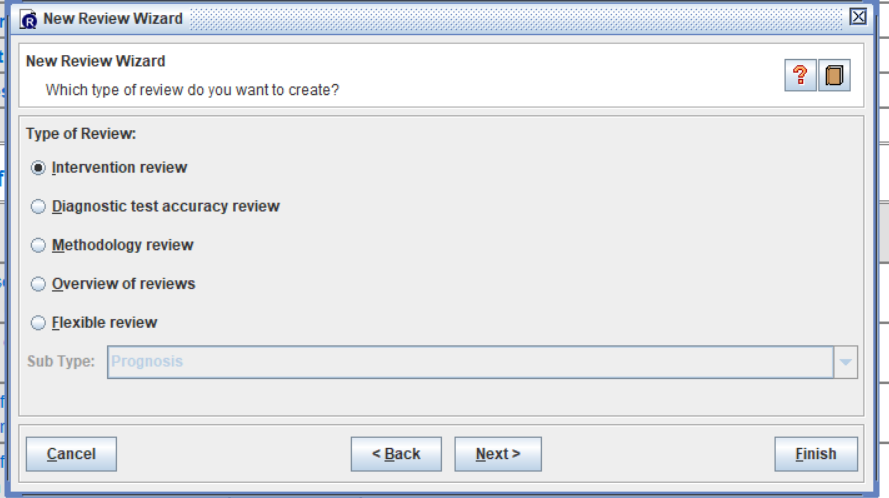
First, click File to create a new study, and select Type of review as intervention review in the pop-up dialog box..

Then click Next and enter the complete title: After non-drug intervention of dysphagia after

stroke, click Next.
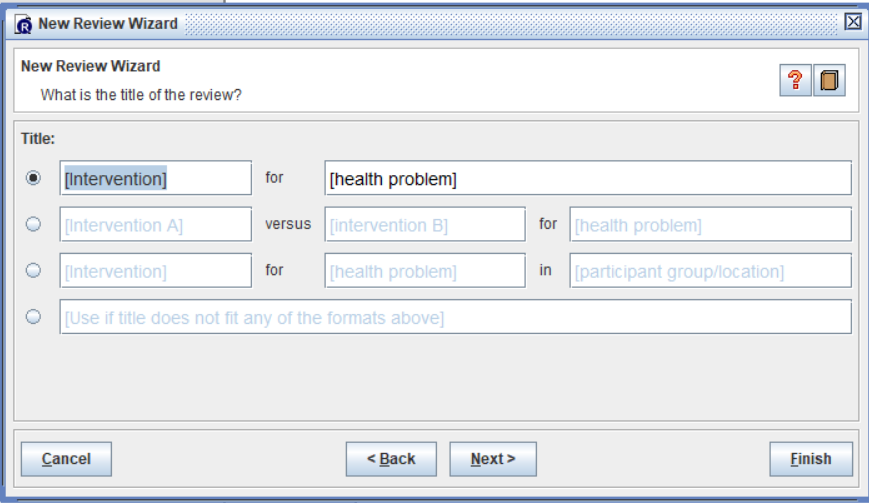


You can add characters of included studies after selecting stage as full review.


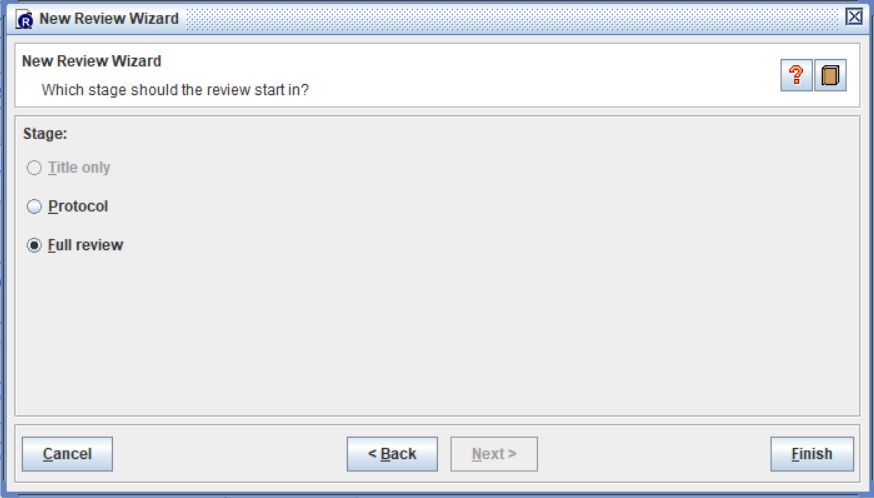


Click tables and characters of studies and characters of included studies in turn, and right-click add study.


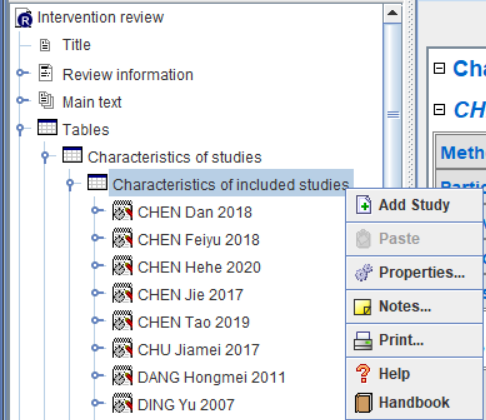


Enter the study id, and we use the first author's name+publication year as the study id. Click

finish.
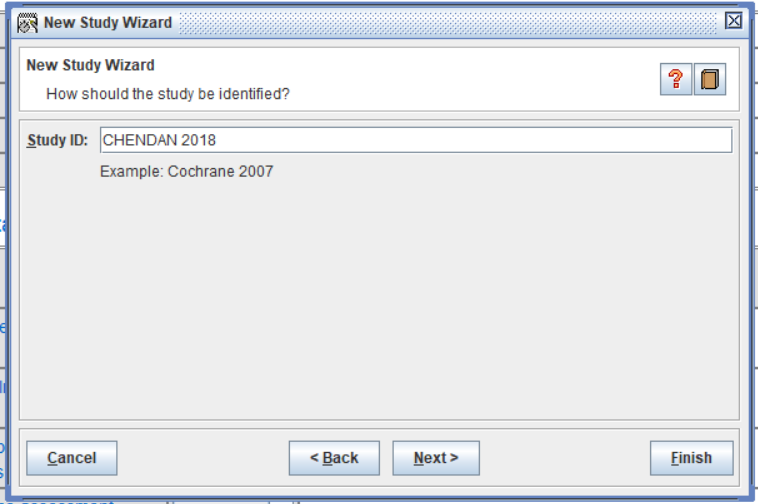


After adding the title of the document, fill in the risk of bias table of the document. Bias includes seven fields, and the risk is divided into low risk and high risk and unclear risk. Fill in all the risk of bias table included in the literature according to the appraiser's results. It should be noted that if the result of bias of risk in a certain field is unclear risk, the support for judgement needs to be filled in.


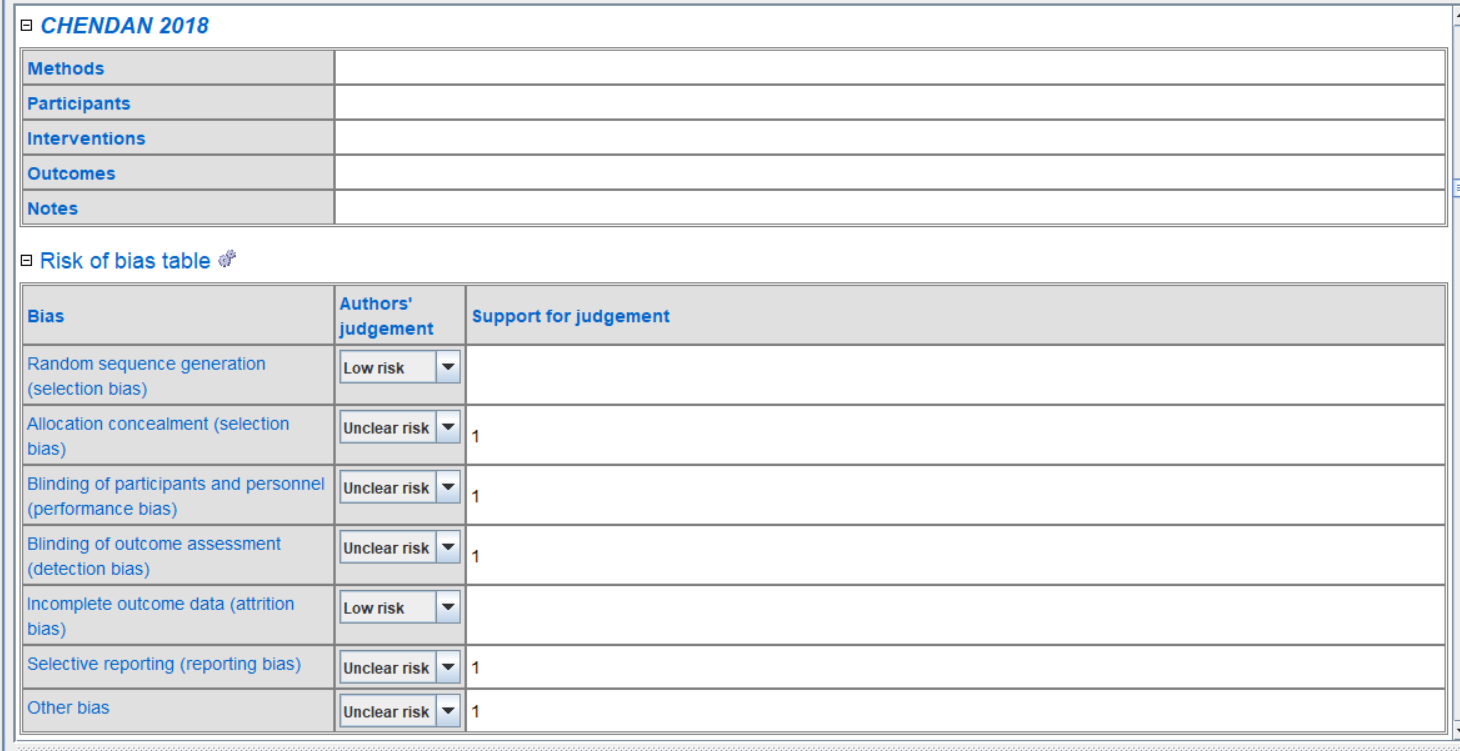


When the risk of bias in all included documents is completed, you can draw figures of risk of bias. Right-click figures and click add figure.


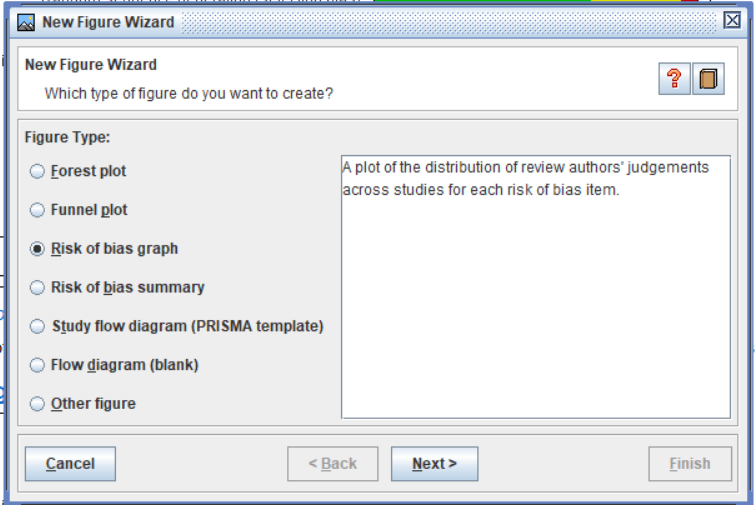
Select figure type as risk of bias graph as needed and click next to get the result.


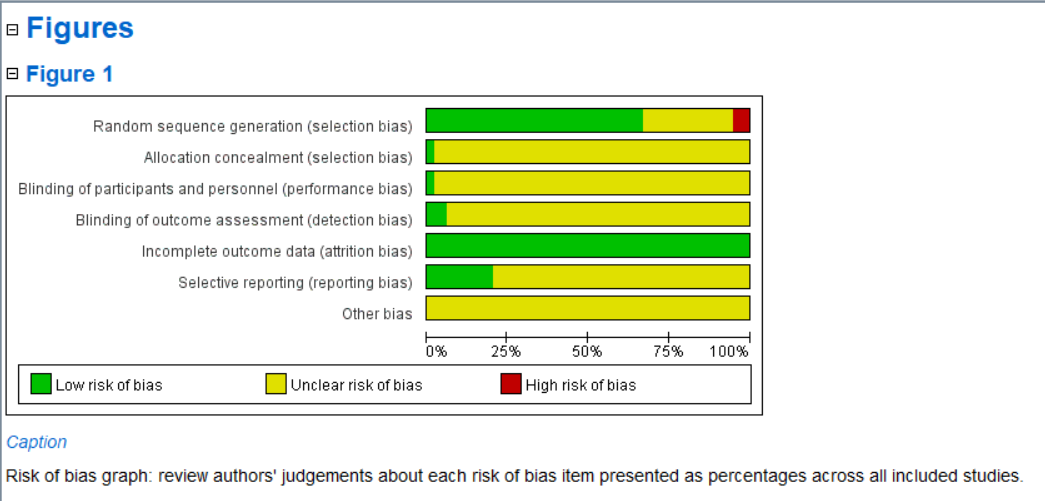


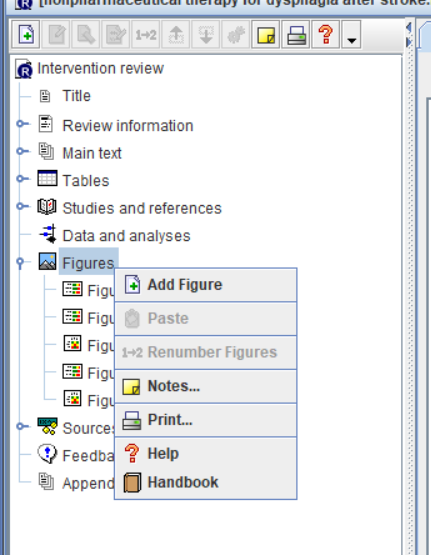

Supplement: Supplementary file 2 — Additional file 2. Software operation. [file 12868_2023_825_MOESM2_ESM.docx]
