# Supplementary material for: Comparison of efficacy of non-pharmacological intervention for post-stroke dysphagia: a systematic review and Bayesian network meta-analysis
Source: BMC Neurosci. 2023 Oct 16;24:53. doi: 10.1186/s12868-023-00825-0 (PMC10578008; doi:10.1186/s12868-023-00825-0)
Supplement: Supplementary file 3 — Additional file 3. Characteristics of each study. [file 12868_2023_825_MOESM3_ESM.docx]

Appendix 3 Characteristics of each study

|  | First author, year | duration | Treatment group | | | control group | | | Adverse events reported | Types of outcomes |
| --- | --- | --- | --- | --- | --- | --- | --- | --- | --- | --- |
|  |  |  | Sample(male/female) | Intervention | Participant age (years) | Sample(male/female) | Intervention | Participant age (years) |  |  |
| 1 | WANG Le 2006 | 3w | 17(7/10) | C | 71.4±5.80 | 14(6/8 ) | D | 69.6±7.60 | NR | ① |
| 2 | YANG ZhenZhong 2006 | 10d | 31 | F | 59 ±16.00 | 31 | C | 59 ±16.00 | NR | ④ |
| 3 | DING Yu 2007 | 30d | 36 | F | 57.5±6.50 | 30 | C | 57.5±6.50 | NR | ④ |
| 4 | KONG Yue-nan 2008 | 2w | 25 | G | 43～75 | 25 | C | 43～75 | NR | ① |
|  |  |  | 25 | F | 43～75 |  |  |  |  |  |
| 5 | YANG Xiao-hui 2008 | 14d | 30(15/15) | A | 65.8 | 30(14/16) | D | 67.3 | NR | ④ |
| 6 | ZHANG Ji-long 2009 | 30d | 65(38/27) | H | 61.6±21.30 | 60(34/26) | D | 58.7±18.50 | NR | ① |
| 7 | MIAO Qing-bo 2009 | 12d | 64(41/23) | F | 42～76 | 62(39/23) | D | 44～75 | NR | ④ |
| 8 | LU Min 2010 | 3w | 15 | F | （60.20±9.55） | 15 | C | （60.20±9.55） | NR | ① |
|  |  |  | 15 | G | （60.20±9.55） |  |  |  |  |  |
| 9 | SU Xuan 2010 | 30d | 30(17/13) | A | 45～80 | 30(20/10) | C | 50～79 | Limb fatigue, sleep, malnutrition, constipation, depressed mood, poor appetite | ①④ |
| 10 | LIU Bo 2010 | 20d | 20 (14/6) | F | 63.25±10.18 | 20(13/7) | C | 65.45±12.57 | NR | ④ |
| 11 | Wenguang XIA 2011 | 4w | 40(23/17) | B | 66.40±15.63 | 40(25/15) | C | 65.32±14.29 | NR | ①②③ |
|  |  |  | 40(28/12) | G | 65.85±14.63 |  |  |  |  |  |
| 12 | YANG Hai-fang 2011 | 3w | 35(24/11) | G | 67.91±10.62 | 35(25/10) | C | 67.37±9.75 | NR | ② |
| 13 | DANG Hong-mei 2011 | 2w | 20(12/8) | B | 55.3 | 20(13/7) | C | 56.1 | NR | ① |
|  |  |  | 20(11/9) | G | 56.6 |  |  |  |  |  |
| 14 | LIAO Chun-lian 2012 | 30d | 56 | G | / | 56 | C | / | NR | ④ |
| 15 | JIN Hai-peng 2013 | 21d | 47 | I | 68.47±7.59 | 43 | A | 67.53±7.27 | NR | ④ |
| 16 | LIU Shidan 2013 | 8w | 30(20//10) | C | 61.3±6.10 | 30(19/11) | D | 62.5±5.50 | NR | ① |
|  |  |  | 30(18/12) | A | 63.1±5.20 |  |  |  |  |  |
|  |  |  | 30(17/13) | B | 60.7±6.80 |  |  |  |  |  |
|  |  |  | 30(16/14) | H | 61.3±5.80 |  |  |  |  |  |
| 17 | HU Xiao-jun 2014 | 4w | 35(31/4) | H | 58.5±6.50 | 35（30/5） | C | 58.0±6.00 | NR | ① |
| 18 | WANG Kai-long 2014 | 4w | 30(16/14) | J | 62.8±9.60 | 30(17/13) | F | 64.5±11.40 | NR | ② |
| 19 | WANG Li-chun 2014 | 14d | 60(36/24) | E | 58.21 | 60(34/26) | A | 59.63 | NR | ④ |
| 20 | ZHANG Shuai 2014 | 60d | 87(58/29) | F | 64.61±9.70 | 87(64/23) | C | 63.86±10.55 | Acute myocardial infarction | ④ |
| 21 | YAN Xiao-jie 2015 | 10d | 32(21/11) | G | 56. 3 ± 5. 40 | 32(19/13) | F | 55. 6 ± 6. 10 | NR | ① |
| 22 | ZHU Zhi-zhong 2015 | 15d | 34(20/14) | G | 61.29 ± 8.45 | 34(22/13) | C | 61.97 ± 9.22 | NR | ①② |
| 23 | WEI Hai-tang 2015 | 2w | 50(27/23) | F | 61.50±4.20 | 50(26/24) | C | 62.50±4.90 | NR | ① |
| 24 | XU Xiang-rong 2015 | 1a | 40(35/5) | F | 55.2 ± 2. 50 | 40(37/3) | C | 57.3 ± 3.00 | NR | ④ |
| 25 | ZHANG Da-bin 2016 | 24d | 45(29/16) | F | 62.4 ± 9.60 | 45(27/18) | C | 61.2 ± 10.10 | NR | ① |
| 26 | ZHANG Yan 2016 | 20d | 20(11/9) | F | 52.8±8.20 | 20(12/8) | G | 54.6±7.10 | NR | ②④ |
|  |  |  | 20(10/10) | H | 55.2±8.70 |  |  |  |  |  |
| 27 | Wenguang Xia 2016 | 4w | 62(35/27) | F | 65.3 ± 14.20 | 62(36/26) | C | 66.1 ± 14.30 | pain, dizziness, bleeding, aggravation, hematoma and infection, and discomfort | ③ |
| 28 | TIAN Tian 2016 | 4w | 31(14/17) | G | 51. 2 ± 2. 30 | 31(17/14) | C | 52. 1 ± 3.10 | NR | ② |
| 29 | NI Rong-fu 2016 | 60d | 25(13/12) | F | 62.15 ± 10.97 | 25(15/10) | C | 61.15±11.82 | NR | ① |
|  |  |  | 25(11/14) | G | 60.35 ± 12.07 |  |  |  |  |  |
|  |  |  | 25(9/16) | H | 63.02 ± 11.72 |  |  |  |  |  |
| 30 | XIAO Yansong 2016 | / | 30(23/7) | F | 80.90±2.41 | 30(22/8) | C | 83.12±3.26 | NR | ②③④ |
| 31 | GAO Sheng 2016 | 4w | 40(20/16) | F | 58.2±5.10 | 40(22/18) | C | 57.8±4.90 | NR | ④ |
| 32 | LIU Yu-mei 2016 | 20d | 31(19/12) | G | 63.4±10.10 | 31(21/10) | F | 58.2±9.20 | NR | ④ |
|  |  |  | 31(22/9) | H | 61.3±10.50 |  |  |  |  |  |
| 33 | ZHANG Qian 2016 | 20d | 20(10/10) | H | 58.30±10.10 | 20(12/8) | G | 58.20±10.10 | NR | ① |
| 34 | FENG Sheng-wang 2016 | 18d | 30(18/12) | F | 60±12.00 | 30(19/11) | C | 58±12.00 | Severe pain | ① |
| 35 | DUAN Xue-fang 2016 | 24d | 25(14/11) | A | 64.40 ± 7.28 | 25(17/8) | C | 64. 96 ± 7.52 | NR | ②④ |
| 36 | CHU Jiamei 2017 | 8w | 48(29/19) | F | 67±11.00 | 49(20/19) | C | 67±10.00 | NR | ②③ |
| 37 | CHEN Jie 2017 | 4w | 42(25/17) | F | 64 ±11.00 | 42(28/14) | C | 61±12 | acupuncture syncope | ④ |
| 38 | MAO Liya 2017 | 20d | 35(20/15) | F | 62.31 ±10.34 | 35(19/16) | G | 61.34±9.63 | NR | ① |
| 39 | NIU Xuekang 2017 | 2w | 30(17/13) | H | 63.28± 11.49 | 30(16/14) | C | 61.42± 12.54 | NR | ②④ |
| 40 | LUO Wan-ying 2017 | 4w | 43(22/21) | G | 66±7.40 | 43(23/20) | C | 67±8.20 | NR | ③ |
| 41 | XIANG Lian 2017 | 4w | 49(25/24) | E | 65.8±6.40 | 49(29/22) | B | 666.2±7.10 | NR | ③ |
| 42 | Zhang Yi 2017 | 28d | 46(28/18) | H | 66.2±7.40 | 46(30/16) | G | 67.5±6.70 | Laryngeal spasm, skin burns, changes in pulse, blood pressure, or heart rate | ① |
| 43 | WANG  Yan-hua 2017 | 15d | 45(20/25) | F | 65±4.00 | 45(22/23) | C | 66±4.00 | NR | ① |
| 44 | ZHU Tao 2017 | 4w | 60(34/26) | H | 52.16 ± 0. 88 | 60(36/24) | G | 51. 95 ± 0.93 | NR | ② |
| 45 | FU Kai-li 2018 | 8w | 25(14/11) | F | 53.5 ± 2. 8 | 25(10/15) | C | 52. 7 ± 4. 8 | NR | ② |
| 46 | CHEN Fei-yu 2018 | 8w | 48(29/19) | F | 67±11.00 | 49(39/10) | C | 67±10.00 | NR | ② |
| 47 | Lise Sproson† 2018 | 4w | 15(10/5) | G | 73±15.30 | 15(9/6) | C | 81±11.00 | NR | ③ |
| 48 | WANG Hai-bo 2018 | 63d | 45(22/20) | G | 62.87±3.57 | 45(23/22) | C | 63.18±3.92 | NR | ①④ |
| 49 | SONG Yuan-yuan 2018 | 4w | 50(30/20) | G | 59.41±16.51 | 50(32/18) | A | 60.25±14.40 | NR | ①②④ |
| 50 | LUO Guo-Liang 2018 | 14d | 35(18/17) | K | 59.60 ± 3.82 | 35(16/19) | C | 60.23 ± 3.97 | NR | ① |
| 51 | YU Guo-yao 2018 | 3w | 55(30/25) | F | 60.5± 10.30 | 55(32/23) | C | 61.7± 9.80 | NR | ②④ |
| 52 | CHEN Dan 2018 | 1m | 30(19/11) | F | 64±5.00 | 30(22/8) | G | 65±5.00 | NR | ①② |
| 53 | WANG Qi 2019 | 2weeks | 46(25/21) | L | 60.89±9.59 | 45(21/24) | C | 64.00±9.81 | No adverse events | ②③④ |
| 54 | LIU Hai-fei 2019 | 8weeks | 74(50/24) | F | 66.64 ± 8.34 | 74(51/23) | C | 65.52 ± 8.55 | palindromia,death | ①②③ |
| 55 | YIN Ya-mei 2019 | 6months | 45(26/19) | C | 66.72 ± 7.56 | 45(27/18) | D | 67.13 ± 7.57 | NR | ① |
| 56 | LAN Gui-chang 2019 | 4weeks | 46(29/17) | E | 66.70±12.40 | 46(28/18) | D | 67.30±10.80 | NR | ④ |
| 57 | WANG Qiao-ling 2019 | 14days | 32(18/14) | E | 64.00±8.00 | 30(17/13) | C | 65.00±9.00 | NR | ②③ |
| 58 | ZHI Jian-mei 2019 | 12weeks | 39(21/18) | F | 63.16 ± 6.92 | 39(19/20) | C | 62.78 ± 6.78 | NR | ④ |
| 59 | LIU Xiao-qing 2019 | 3weeks | 40(28/12) | H | 68.20±9.43 | 40(27/13) | F | 66.88±13.09 | NR | ②③ |
| 60 | WANG Jia 2019 | 6weeks | 30(21/9) | F | 55.86±8.93 | 30(17/13) | C | 56.12±9.04 | NR | ③④ |
| 61 | QIN He-wei 2019 | 8weeks | 50(25/25) | F | 47.10±6.30 | 50(26/24) | C | 46.10±6.90 | NR | ①④ |
| 62 | XU Ying 2019 | 6weeks | 48(27/21) | H | 67.20±11.50 | 48(28/20) | G | 66.90±12.60 | NR | ③④ |
| 63 | CHEN Tao 2019 | 30days | 89(54/35) | F | 62.30±5.50 | 88(52/36) | C | 62.50±5.60 | NR | ①② |
| 64 | HU Yan-hua 2019 | 3months | 49(33/16) | F | 65.84±5.25 | 49(31/18) | C | 64.87±7.35 | NR | ④ |
| 65 | HUANG Jian-ting 2019 | 8weeks | 34(23/11) | F | 66.10±13.80 | 35(20/15) | C | 64.40±13.70 | NR | ② |
| 66 | ZHONG Yin-yan 2019 | 3weeks | 40(24/16) | F | 64.50±8.40 | 40(25/15) | C | 62.30±8.10 | NR | ④ |
| 67 | HE Qing-song 2019 | 8weeks | 60(43/17) | F | 62.16 ± 7.04 | 60(40/20) | C | 61.83 ± 6.81 | NR | ②④ |
| 68 | ZHANG Xiao-xia 2020 | 4weeks | 30(17/13) | F | 56.53±10.55 | 30(14/16) | C | 58.30±10.66 | NR | ①④ |
| 69 | YUAN Ying 2020 | 4weeks | 38(28/10) | I | 63.00±9.00 | 37(27/10) | C | 63.00±8.00 | NR | ③ |
| 70 | HUANG Jian-fu 2020 | 4weeks | 45(28/17) | E | 66.27±7.63 | 45(26/19) | A | 65.83±7.21 | Aspiration | ②③ |
| 71 | GAO Jiaxiu 2020 | 4weeks | 30(20/10) | F | 65.00±5.00 | 30(23/7) | C | 64.00±5.00 | NR | ①② |
| 72 | ZHANG Ling 2020 | 4weeks | 62(37/25) | F | 67.03 ± 6.25 | 61(34/27) | C | 66.81±6.03 | NR | ①②④ |
| 73 | LI Hong-rui 2020 | 8weeks | 32(19/13) | G | 56.50± 7.68 | 32(20/12) | H | 56.69 ± 8.01 | NR | ② |
| 74 | LI Yang 2020 | 2weeks | 33(11/12) | L | 54.00±9.10 | 33(13/10) | C | 53.00±8.10 | Dizziness,naupathia | ③④ |
| 75 | GAO Jia-xiu 2020 | 4weeks | 42(30/12) | F | 62.95 ± 8.99 | 42(28/14) | C | 62.43 ± 10.12 | NR | ② |
| 76 | CHEN He-he 2020 | 4weeks | 58(34/24) | F | 59.84±11.37 | 58(35/23) | C | 58.94±11.83 | NR | ①③ |
| 77 | DONG Yan 2021 | 1month | 53(36/17) | F | 65.02±6.58 | 53(39/14) | C | 63.13±5.57 | NR | ④ |
| 78 | WANG Yanan 2021 | 4weeks | 40(25/15) | L | 59.62±4.89 | 40(22/18) | C | 60.33±5.27 | NR | ③④ |
| 79 | ZHAO Shang 2021 | 4weeks | 65(41/24) | H | 62.17±2.42 | 65(38/27) | F | 61.28±2.45 | Pulmonary infection | ②③ |
| 80 | WANG Ling-shu 2021 | 3weeks | 38(23/15) | F | 57.41±5.45 | 38(21/17) | C | 56.84±3.36 | NR | ①③ |
| 81 | LI Yan-jiao 2021 | 4weeks | 20(15/5) | F | 53.00 ± 6.09 | 20(13/7) | C | 53.80±7.04 | No adverse events | ②④ |
| 82 | LI Xiao-yan 2021 | 20days | 42(25/17) | F | 56.52±4.93 | 42(23/19) | C | 57.21±3.84 | NR | ③ |
| 83 | HE Zi-long 2021 | 8weeks | 35(20/15) | G | 58.11±5.24 | 35(21/14) | C | 58.31±4.58 | NR | ③ |
| 84 | LV Xue-hai 2021 | 3months | 45(19/26) | H | 60.11±8.87 | 45(17/28) | C | 61.62±7.55 | NR | ①②③ |
| 85 | JIN Bei 2021 | 4weeks | 45(24/21) | F | 57.38±12.65 | 45(26/19) | C | 57.75±12.76 | Insomnia,fever | ①③④ |
| 86 | QI Yu-jun 2021 | 2weeks | 60(36/24) | F | 63.00±10.00 | 60(33/27) | C | 63.00±11.00 | Subcutaneous congestion | ①②④ |
| 87 | HAN Rong 2021 | 2months | 50(25/25) | C | 63.06±11.50 | 50(27/23) | D | 62.94±11.32 | NR | ②③④ |
| 88 | MAO Lan-ping 2021 | 4weeks | 46(29/17) | F | 50.97±7.13 | 46(31/15) | C | 51.36±6.72 | No adverse events | ② |
| 89 | WANG Ya-nan 2021 | 15days | 41(23/18) | E | 55.35±3.25 | 41(24/17) | B | 55.29±3.42 | NR | ④ |
| 90 | LIANG2022 | 4weeks | 32(22/10) | A | 57.00±8.00 | 32(20/12) | C | 58.00±7.00 | NR | ② |
| 91 | LIN Qian 2022 | 4weeks | 30(18/12) | H | 61.00±14.00 | 30(21/9) | G | 60.00±13.00 | No adverse events | ④ |
| 92 | RAO Jin-zhu 2022 | 2weeks | 33(22/11) | G | 63.42±10.35 | 31(24/7) | C | 65.9±11.42 | Dizziness | ② |
| 93 | FENG Ya 2022 | 1month | 57(33/24) | G | 63.31±13.4 | 57(30/26) | C | 64.75±12.7 | NR | ①② |
| 94 | ZHANG Xue-ling 2022 | 3months | 45(31/14) | K | 61.30±3.18 | 45(30/15) | C | 61.47±3.22 | No adverse events | ②③④ |
| 95 | YU Jing 2022 | 3weeks | 21(12/9) | H | 71.00±7.00 | 21(11/10) | G | 71.00±6.00 | No adverse events | ③ |
| 96 | YANG Jing 2022 | 4weeks | 35(21/14) | F | 57.00±9.00 | 36(24/12) | C | 59.00±6.00 | No adverse events | ② |
