# Supplementary material for: Comparison of efficacy of non-pharmacological intervention for post-stroke dysphagia: a systematic review and Bayesian network meta-analysis
Source: BMC Neurosci. 2023 Oct 16;24:53. doi: 10.1186/s12868-023-00825-0 (PMC10578008; doi:10.1186/s12868-023-00825-0)
Supplement: Supplementary file 4 — Additional file 4. Qualitative and meta-analysis of two-by-two comparisons of interventions. [file 12868_2023_825_MOESM4_ESM.docx]

**Appendix 4 Qualitative and meta-analysis of two-by-two comparisons of interventions**

**A compared with B**

(1) VFSS: The results of one qualitative RCT analysis showed no significant difference in the efficacy of VFSS between A and B [MD=0.01，95%CI(－0.65, 0.67)，P=0.98].


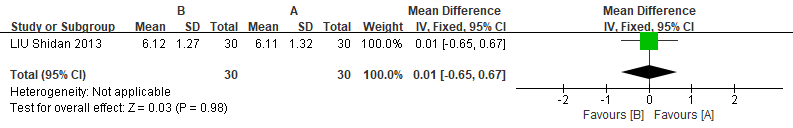


Appendix 4-figure 1 Forest plot of VFSS of A compared with B
The letters in the figure represent the 12 non-pharmacological interventions. A means acupuncture; B means electrotherapy; C means rehabilitation training; D means conventional treatment; E means acupuncture+electrotherapy; F means acupuncture+rehabilitation training; G means electrotherapy+rehabilitation training; H means acupuncture+electrotherapy+rehabilitation training; I means acupoints sticking; J means acupuncture+rehabilitation training+massage; K means rehabilitation training+acupoints sticking; L means acupuncture+rehabilitation training+acupoints sticking. The following are the same.

(2) SSA: There was no direct comparison.

(3) SWAL: There was no direct comparison.

(4) WST: There was no direct comparison.

**A compared with C**

(1) VFSS: Two studies reported on VFSS, including 120 patients, 60 in group A and 60 in group C. The homogeneity (P=0.39, I^2^=0%) was great and analyzed by fixed effects model. The results showed that A could improved VFSS significantly than C [MD=0.64, 95%CI (0.05, 1.23), P=0.03].


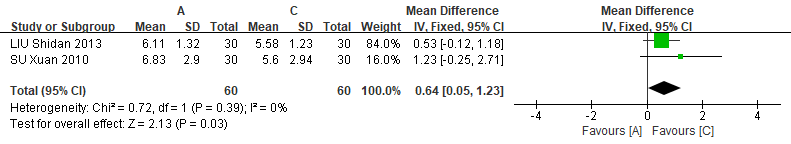


Appendix 4-figure 2 Forest plot of VFSS of A compared with C

Legend of the figure is same as Appendix 4-figure 1.

(2) SSA: Two studies reported on SSA, including 114 patients, 57 in group A and 57 in group C. Great interstudy homogeneity (P=0.88, I^2^=0%) was analyzed by a fixed-effects model. The results showed that A decreased SSA better than C, which was statistically significant [MD=-1.48, 95%CI(-2.36,-0.59), P=0.001].


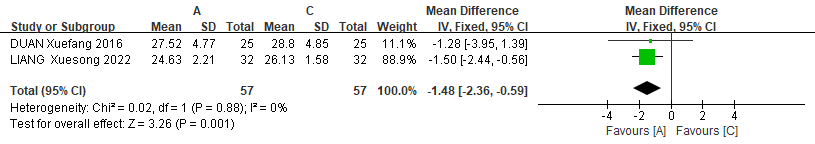


Appendix 4-figure 3 Forest plot of SSA of A compared with C

Legend of the figure is same as Appendix 4-figure 1.

(3) SWAL: There was no direct comparison.

(4) WST: Two studies reported on WST, including 110 patients, 55 in group A and 55 in group C. Great interstudy homogeneity (P=0.39, I^2^=0%) was analyzed by a fixed-effects model. The results showed that C reduced WST better than A, which was statistically significant [MD=-0.64, 95%CI (0.05,1.23), P=0.03].


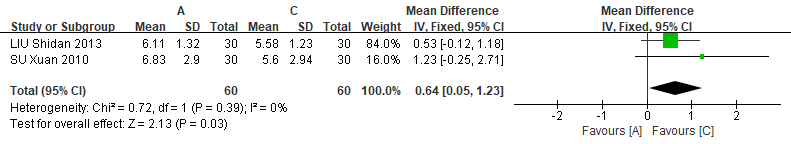


Appendix 4-figure 4 Forest plot of WST of A compared with C

Legend of the figure is same as Appendix 4-figure 1.

**A compared with D**

(1) VFSS: The results of one qualitative RCT analysis showed that A was better at improving VFSS thanD, and there was a statistically significant difference [MD=-1.04, 95%CI(-1.51, -0.57), P＜0.0001].


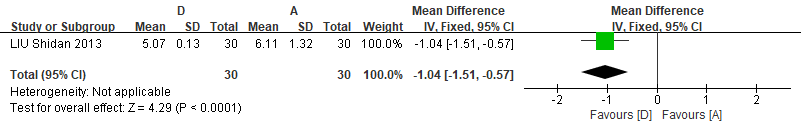


Appendix 4-figure 5 Forest plot of VFSS of A compared with D

Legend of the figure is same as Appendix 4-figure 1.

(2) SSA: There was no direct comparison.

(3) SWAL: There was no direct comparison.

(4) WST: The results of one qualitative analysis of RCT showed that A had a better effect in reducing WST than D, with a significant difference [MD=2.19, 95%CI(1.60,2.78), P＜0.00001].


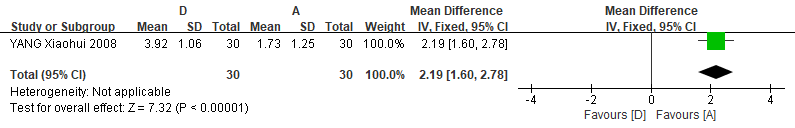


Appendix 4-figure 6 Forest plot of WST of A compared with D

Legend of the figure is same as Appendix 4-figure 1.

**A compared with E**

(1) VFSS: There was no direct comparison.

(2) SSA: The results of one qualitative analysis of RCT showed that E was better in reducing SSA than A, with a statistically significant difference [MD=-4.57, 95%CI(-6.40,-2.74), P＜0.00001].


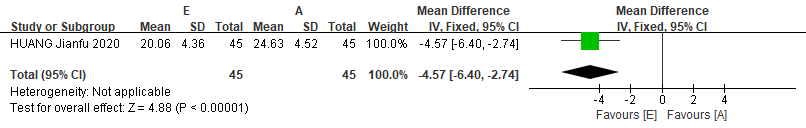


Appendix 4-figure 7 Forest plot of SSA of A compared with E

Legend of the figure is same as Appendix 4-figure 1.

(3) SWAL: The results of one qualitative RCT analysis showed that E improved SWAL better than A, which was statistically significant [MD=25.12, 95%CI(18.96,31.28), P＜0.00001].


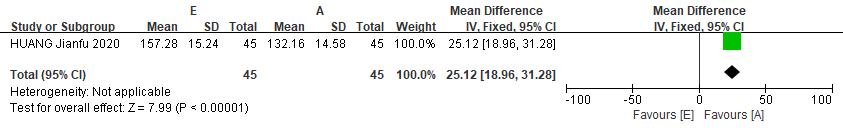


Appendix 4-figure 8 Forest plot of SWAL of A compared with E

Legend of the figure is same as Appendix 4-figure 1.

(4) WST: The results of one qualitative RCT analysis showed that E decreased WST better than A, and the difference was statistically significant [MD=-1.06, 95%CI(-1.66,-0.46), P=0.0005].


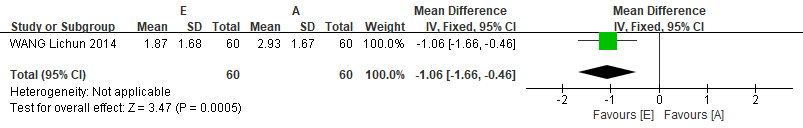


Appendix 4-figure 9 Forest plot of WST of A compared with E

Legend of the figure is same as Appendix 4-figure 1.

**A compared with F**

(1) VFSS: There was no direct comparison.

(2) SSA: There was no direct comparison.

(3) SWAL: There was no direct comparison.

(4) WST: There was no direct comparison.

**A compared with G**

(1) VFSS: The results of one RCT qualitative analysis showed that G had better effects in improving VFSS than A, and the difference was statistically significant [MD=2.59, 95%CI(2.12, 3.06), P＜0.00001].


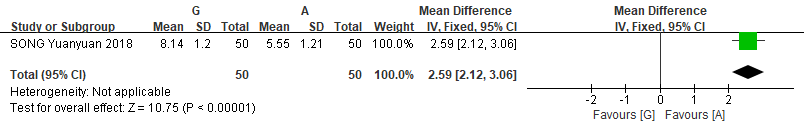


Appendix 4-figure 10 Forest plot of VFSS of A compared with G

Legend of the figure is same as Appendix 4-figure 1.

(2) SSA: The results of one qualitative analysis of RCT showed that G had better effects in reducing SSA than A, with a statistically significant difference [MD=-2.70, 95%CI(-3.82,-1.58), P＜0.00001].


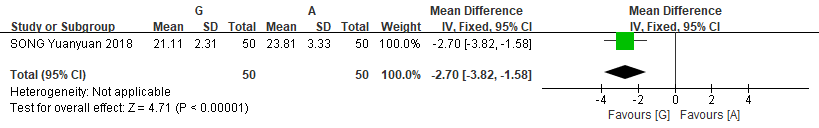


Appendix 4-figure 11 Forest plot of SSA of A compared with G

Legend of the figure is same as Appendix 4-figure 1.

(3) SWAL: There was no direct comparison.

(4) WST: The results of one qualitative analysis of RCT showed that G had a better effect in reducing WST than A, which was statistically significant [MD=-0.96, 95%CI(-1.20,-0.72), P＜0.00001].


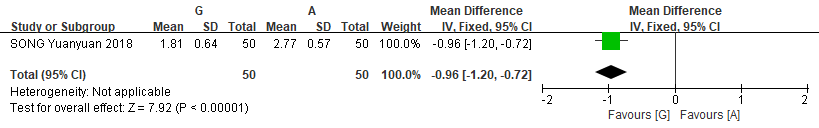


Appendix 4-figure 12 Forest plot of WST of A compared with G

Legend of the figure is same as Appendix 4-figure 1.

**A compared with H**

(1) VFSS: The results of one qualitative RCT analysis showed that the H had a better effect in improving VFSS than A, and the difference was statistically significant [MD=2.12, 95%CI(1.45, 2.79), P＜0.00001].

**
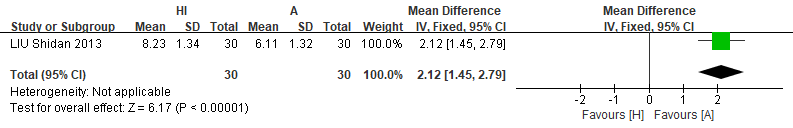
**

Appendix 4-figure 13 Forest plot of VFSS of A compared with H

Legend of the figure is same as Appendix 4-figure 1.

(2) SSA: There was no direct comparison.

(3) SWAL: There was no direct comparison.

(4) WST: There was no direct comparison.

**A compared with I**

(1) VFSS: There was no direct comparison.

(2) SSA: There was no direct comparison.

(3) SWAL: There was no direct comparison.

(4) WST: The results of one qualitative analysis of RCT showed that I could reduce WST better than A, with a statistically significant difference [MD=-0.98, 95%CI(-1.37,-0.59), P＜0.00001].


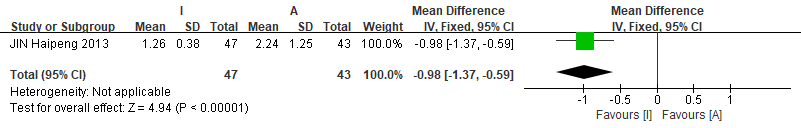


Appendix 4-figure 14 Forest plot of WST of A compared with I

Legend of the figure is same as Appendix 4-figure 1.

**A compared with J**

(1) VFSS: There was no direct comparison.

(2) SSA: There was no direct comparison.

(3) SWAL: There was no direct comparison.

(4) WST: There was no direct comparison.

**A compared with K**

(1) VFSS: There was no direct comparison.

(2) SSA: There was no direct comparison.

(3) SWAL: There was no direct comparison.

(4) WST: There was no direct comparison.

**A compared with L**

(1) VFSS: There was no direct comparison.

(2) SSA: There was no direct comparison.

(3) SWAL: There was no direct comparison.

(4) WST: There was no direct comparison.

**B compared with C**

(1) VFSS: Three studies reported VFSS, including 180 patients, 90 in group B and 90 in group C. The homogeneity (P=0.69, I^2^=0%) was great and analyzed by fixed effects model. The results showed that B was better in improving VFSS than group C, which was statistically significant [MD=0.31, 95%CI(-0.01, 0.63), P=0.05].

**
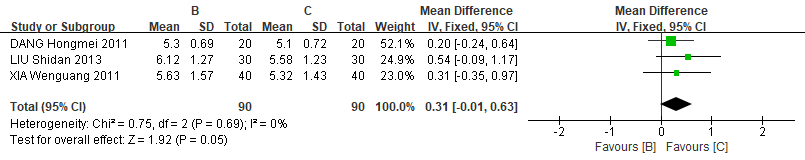
** Appendix 4-figure 15 Forest plot of VFSS of B compared with C

Legend of the figure is same as Appendix 4-figure 1.

(2) SSA: The results of one qualitative RCT analysis showed no significant difference between B and C on SSA [MD= -0.50, 95%CI (-2.26,1.26), P=0.58].

**
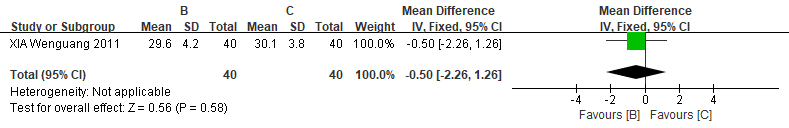
**

Appendix 4-figure 16 Forest plot of SSA of B compared with C

Legend of the figure is same as Appendix 4-figure 1.

(3) SWAL: The results of one qualitative RCT analysis showed that C improved SWAL better than B, which was statistically significant [MD=-166.00, 95%CI(-183.67,-148.33), P＜0.00001].

**
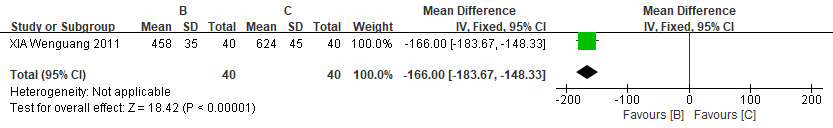
**

Appendix 4-figure 17 Forest plot of SWAL of B compared with C

Legend of the figure is same as Appendix 4-figure 1.

(4) WST: There was no direct comparison.

**B compared with D**

(1) VFSS: The results of one RCT qualitative analysis showed that B had better effects in improving VFSS than D, and the difference was statistically significant [MD=-1.05, 95%CI(-1.51, -0.59), P＜0.00001].


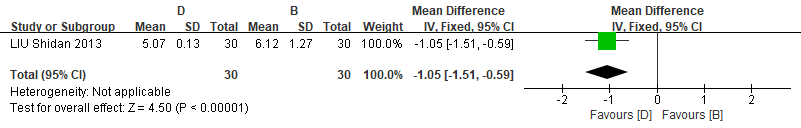


Appendix 4-figure 18 Forest plot of VFSS of B compared with D

Legend of the figure is same as Appendix 4-figure 1.

(2) SSA: There was no direct comparison.

(3) SWAL: There was no direct comparison.

(4) WST: There was no direct comparison.

**B compared with E**

(1) VFSS: There was no direct comparison.

(2) SSA: There was no direct comparison.

(3) SWAL: The results of one qualitative RCT analysis showed that B was better at improving SWAL than E, which was statistically significant [MD=-108.79, 95%CI(-119.22,-98.36), P＜0.00001].


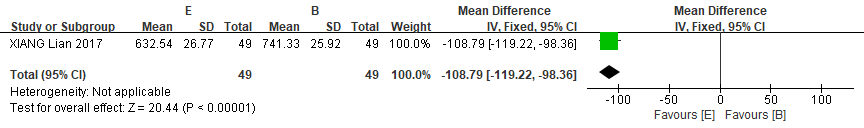


Appendix 4-figure 19 Forest plot of SWAL of B compared with E

Legend of the figure is same as Appendix 4-figure 1.

(4) WST: The results of one qualitative RCT analysis showed that E was more significant in reducing WST than B [MD=-1.35, 95%CI(-1.54,-1.16), P＜0.00001].


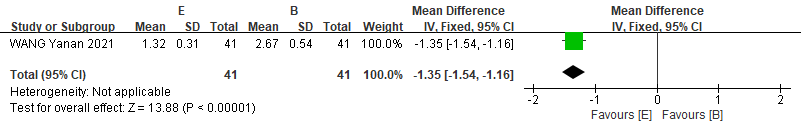


Appendix 4-figure 20 Forest plot of WST of B compared with E

Legend of the figure is same as Appendix 4-figure 1.

**B compared with F**

(1) VFSS: There was no direct comparison.

(2) SSA: There was no direct comparison.

(3) SWAL: There was no direct comparison.

(4) WST: There was no direct comparison.

**B compared with G**

(1) VFSS: The results of qualitative RCT showed that G had a better effect in improving VFSS than B, with a statistically significant difference [MD=1.25, 95%CI(0.56, 1.94), P=0.0004].

**
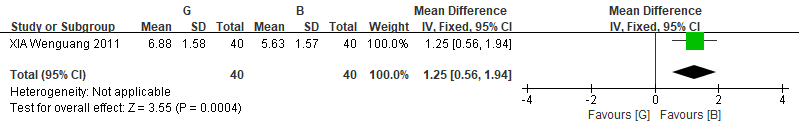
** Appendix 4-figure 21 Forest plot of VFSS of B compared with G

Legend of the figure is same as Appendix 4-figure 1.

(2) SSA: The results of one RCT qualitative analysis showed that G had a better effect in reducing SSA than B, and the difference was statistically significant [MD=8.20, 95%CI(6.51,9.89), P＜0.00001].

**
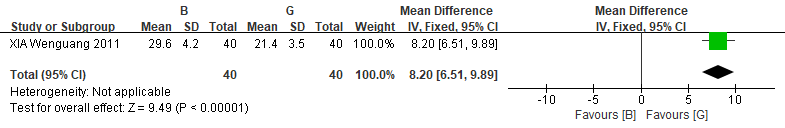
**

Appendix 4-figure 22 Forest plot of SSA of B compared with G

Legend of the figure is same as Appendix 4-figure 1.

(3) SWAL: The results of one qualitative RCT showed that G improved improved SWAL than B, and the difference was statistically significant [MD=-166.00, 95%CI(-183.67,-148.33), P＜0.00001].

**
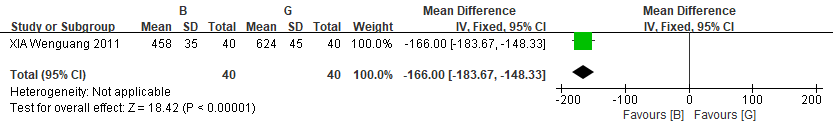
**

Appendix 4-figure 23 Forest plot of SWAL of B compared with G

Legend of the figure is same as Appendix 4-figure 1.

(4) WST: There was no direct comparison.

**B compared with H**

(1) VFSS: The results of one qualitative RCT analysis showed that H had better effects in improving VFSS than B, and the difference was statistically significant [MD=2.11, 95%CI(1.45, 2.77), P＜0.00001].

**
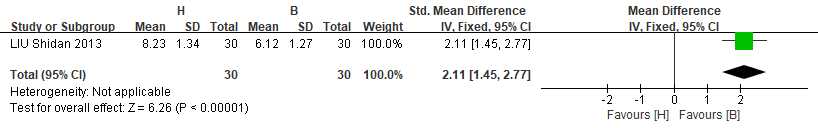
**

Appendix 4-figure 24 Forest plot of VFSS of B compared with H

Legend of the figure is same as Appendix 4-figure 1.

(2) SSA: There was no direct comparison.

(3) SWAL: There was no direct comparison.

(4) WST: There was no direct comparison.

**B compared with I**

(1) VFSS: There was no direct comparison.

(2) SSA: There was no direct comparison.

(3) SWAL: There was no direct comparison.

(4) WST: There was no direct comparison.

**B compared with J**

(1) VFSS: There was no direct comparison.

(2) SSA: There was no direct comparison.

(3) SWAL: There was no direct comparison.

(4) WST: There was no direct comparison.

**B compared with K**

(1) VFSS: There was no direct comparison.

(2) SSA: There was no direct comparison.

(3) SWAL: There was no direct comparison.

(4) WST: There was no direct comparison.

**B compared with L**

(1) VFSS: There was no direct comparison.

(2) SSA: There was no direct comparison.

(3) SWAL: There was no direct comparison.

(4) WST: There was no direct comparison.

**C compared with D**

(1) VFSS: Two studies reported VFSS and included 91 patients, 44 in Group D and 47 in Group C. High heterogeneity among studies (P=0.01, I^2^=84%), using random effects model analysis. The results showed that C had better in improving VFSS than D, and the difference was statistically significant [MD=-0.67, 95%CI(-1.10, -0.25), P=0.002].

**
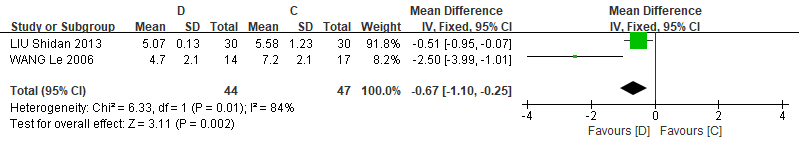
** Appendix 4-figure 25 Forest plot of VFSS of C compared with D

Legend of the figure is same as Appendix 4-figure 1.

(2) SSA: The results of one qualitative analysis of RCT showed that C was more effective in reducing SSA than D, which was statistically significant [MD=-5.12, 95%CI(-6.32,-3.92), P＜0.00001].

**
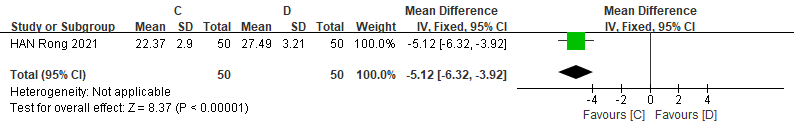
**

Appendix 4-figure 26 Forest plot of SSA of C compared with D

Legend of the figure is same as Appendix 4-figure 1.

(3) SWAL: The results of one qualitative analysis of RCT showed that C improved SWAL better than D, and the difference was statistically significant [MD=169.45, 95%CI(124.60,214.30), P＜0.00001].

**
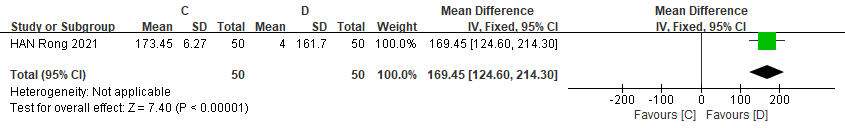
**

Appendix 4-figure 27 Forest plot of SWAL of C compared with D

Legend of the figure is same as Appendix 4-figure 1.

(4) WST: There was no direct comparison.

**C compared with E**

(1) VFSS: There was no direct comparison.

(2) SSA: The results of one qualitative analysis of RCT showed that E decreased SSA better than C, which was statistically significant [MD=-4.90, 95%CI(-7.44,-2.36), P=0.0002].


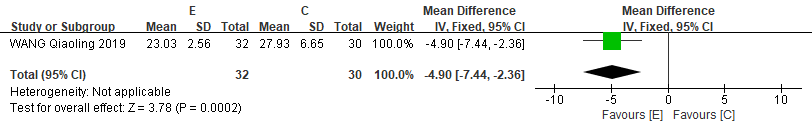


Appendix 4-figure 28 Forest plot of SSA of C compared with E

Legend of the figure is same as Appendix 4-figure 1.

(3) SWAL: The results of one qualitative RCT analysis showed that E improved SWAL than C, and the difference was significant [MD=15.50, 95%CI(9.63,21.37), P＜0.00001].


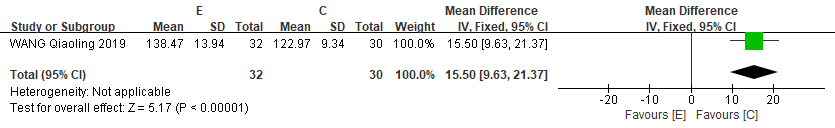


Appendix 4-figure 29 Forest plot of SWAL of C compared with E

Legend of the figure is same as Appendix 4-figure 1.

(4) WST: There was no direct comparison.

**C compared with F**

(1) VFSS: 17 studies reported VFSS, and a total of 1480 patients were enrolled, 741 in group D and 739 in group C. The results of these studies were summarized as follows. Heterogeneity among studies was large (P<0.00001, I^2^=89%) and was analyzed by random effects model. The results showed that F improved VFSS better than C, and the difference was statistically significant [MD=1.85, 95% CI (1.48, 2.22), P<0.00001].

To explore the source of heterogeneity, subgroup analyses were performed based on sample size and duration of treatment.


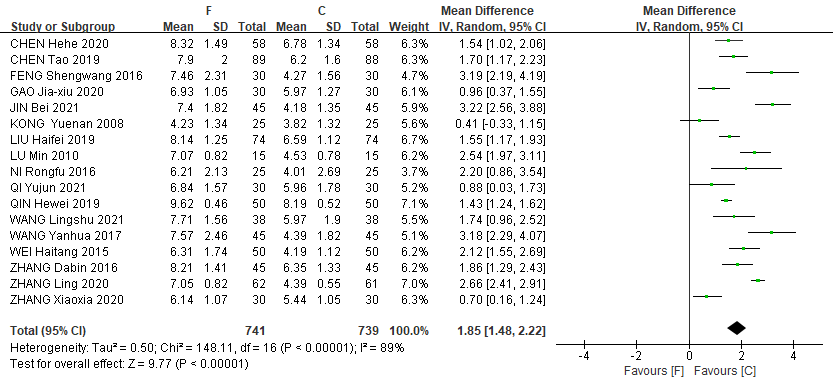


Appendix 4-figure 30 Forest plot of VFSS of C compared with F

Legend of the figure is same as Appendix 4-figure 1.

To explore the source of heterogeneity, subgroup analyses were performed based on differences in duration of treatment. The results showed that differences in duration of treatment may account for the heterogeneity among the included studies of VFSS. ① Thirteen studies with a duration of treatment <4 weeks were included, and the heterogeneity among studies was large (P<0.00001, I^2^=90%). The results showed thatF improved VFSS better than C, and the difference was statistically significant [MD=1.91, 95% CI (1.41,-2.41), P<0.000 01]. ②Four studies with a duration of treatment >4 weeks were included, and heterogeneity among studies was reduced (P=0.55, I^2^=0%). The results showed that F improved VFSS better than C. The difference was statistically significant [MD=1.49, 95%CI(1.33,1.65), P<0.00001].


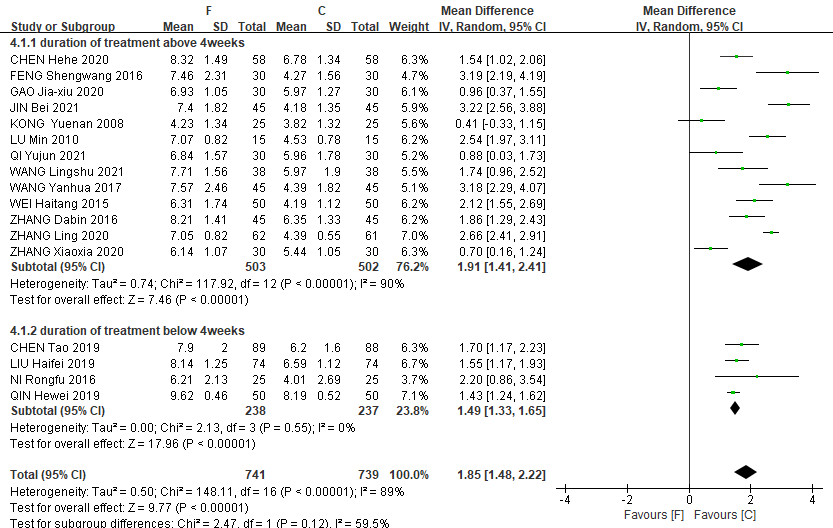


Appendix 4-figure 31 Subgroup’ s forest plot of VFSS of C compared with F

Legend of the figure is same as Appendix 4-figure 1.

(2) SSA: 13 studies reported a total of 1458 patients enrolled in SSA, 728 in group F and 730 in Group C.

Heterogeneity among studies was large (P<0.00001, I^2^=89%) and was analyzed using a random effects model. The results showed that F reduced SSA better than C, and the difference was statistically significant [MD=-3.88, 95% CI (-4.85,-2.91), P<0.00001].

To explore the source of heterogeneity, subgroup analyses were performed based on differences in disease duration. The results showed that differences in disease duration may account for the heterogeneity among the included studies of SSA. ①Nine studies with disease duration <3 months were included, and the heterogeneity among studies was reduced (P=0.09, I^2^=41%). The results showed that F reduced SSA better than C. The difference was statistically significant [MD=-3.99, 95%CI (-4.60,-3.37), P<0.00001]. ② Five studies with a disease duration of >3 months were included, with greater heterogeneity among studies (P<0.00001, I^2^=95%). The results showed that the F reduced SSA better than C, and the difference was statistically significant [MD=-3.97, 95%CI (-6.36,-1.57), P=0.001]. ③Two studies with a disease duration of <3 weeks were included, with reduced heterogeneity between studies (P<0.00001, I^2^=97%). The difference in SSA-lowering effect between the two groups was not statistically significant [MD=-4.09, 95% CI (-8.51,0.33), P=0.07].


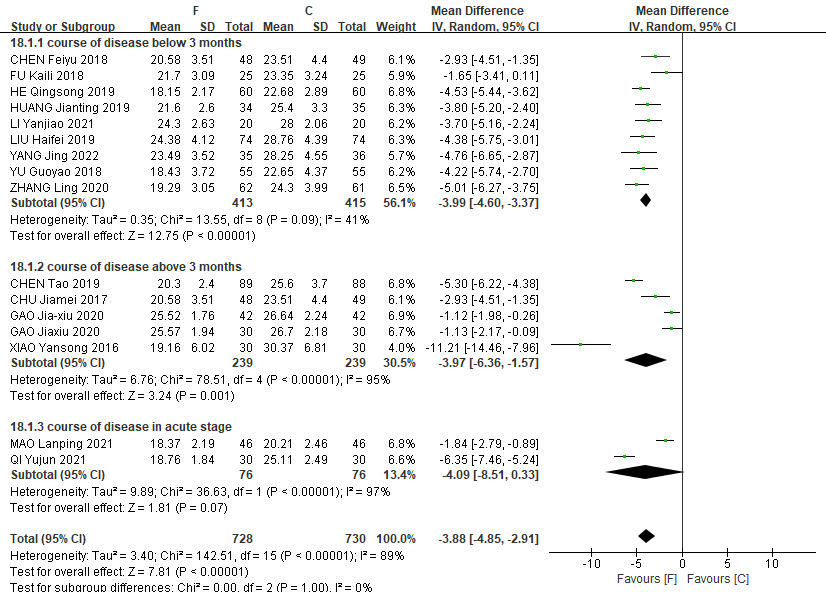


Appendix 4-figure 32 Forest plot of SSA of C compared with F

Legend of the figure is same as Appendix 4-figure 1.

(3) SWAL: 9 studies reported a total of 855 patients enrolled in SWAL, 427 in group F and 428 in group C. The results of these studies were summarized as follows.

Heterogeneity among studies was large (P<0.0001, I^2^=92%) and was analyzed using a random effects model. The results showed that F improved SWAL better than C, and the difference was statistically significant [MD=23.45, 95% CI (17.94,28.97), P<0.00001].

To explore the source of heterogeneity, subgroup analysis was performed based on the difference in duration of treatment. The results showed that differences of duration of treatment may account for the heterogeneity among the included studies of SWAL. ① Six studies with a duration of treatment >4 weeks were included, and the heterogeneity among studies was reduced (P=0.01, I^2^=65%). The results showed that F improved SWAL better than C, and the difference was statistically significant [MD=-24.97, 95% CI (19.80,30.13), P<0.00001]. ② Three studies with a duration of treatment <4 weeks were included, and the heterogeneity among the studies was greater (P<0.00001, I^2^=97%). The results showed that F improved the effect of SWAL better than C. The difference was statistically significant [MD=-20.99, 95% CI (10.95,31.03), P<0.00001].


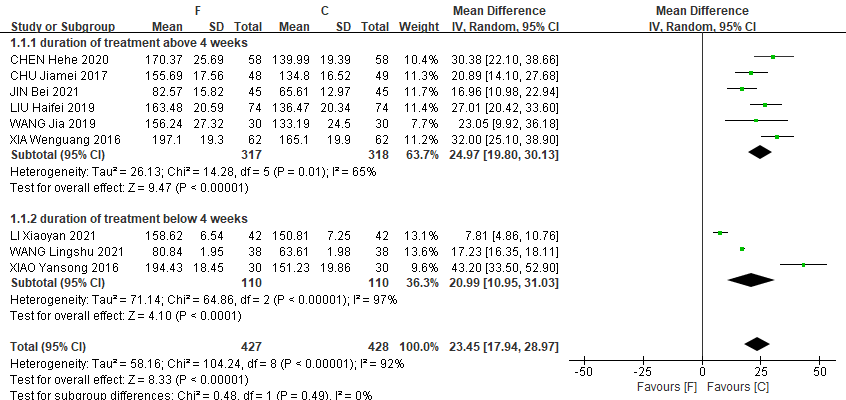


Appendix 4-figure 33 Forest plot of SWAL of C compared with F

Legend of the figure is same as Appendix 4-figure 1.

(4) WST: 21 studies reported WST, including 1771 patients, 889 in group F and 882 in Group C. High heterogeneity among studies (P <0.00001, I^2^=98%), and a random effects model analysis was used. The results showed that F had better effects in reducing WST than C. The difference was statistically significant [MD=-0.91, 95%CI(-1.21,-0.62), P＜0.00001].


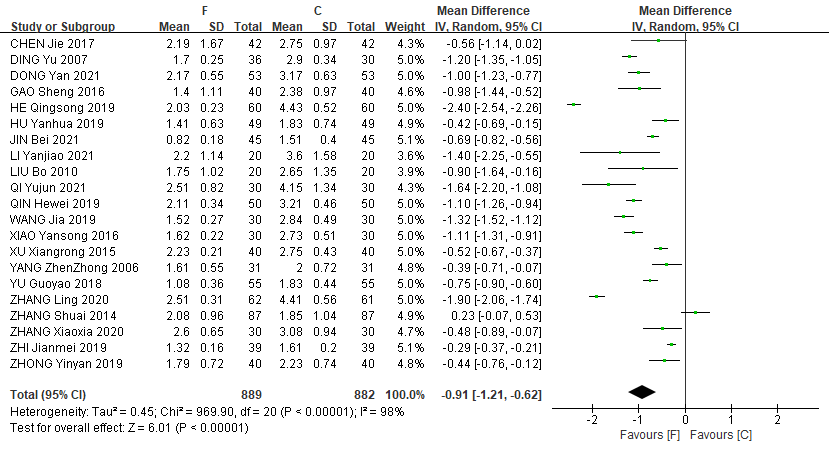


Appendix 4-figure 34 Forest plot of WST of C compared with F

Legend of the figure is same as Appendix 4-figure 1.

To explore the source of heterogeneity, subgroup analyses were performed based on different sample sizes. The results showed that different sample sizes might be the reason for the heterogeneity among the included studies of WST. ① Fourteen studies with a sample size >60 were included, and the heterogeneity among studies was large (P<0.00001, I^2^=99%). The results showed that F reduced WST better than C, and the difference was statistically significant [MD=-0.84, 95%CI(-1.23,-0.45), P<0.00001]. ② Seven studies with a sample size <60 were included, and heterogeneity among studies was reduced (P=0.006, I^2^=65%). The results showed that F reduced WST better than C, and the difference was statistically significant [MD=-1.14, 95% CI (-1.34,-0.94), P<0.00001].


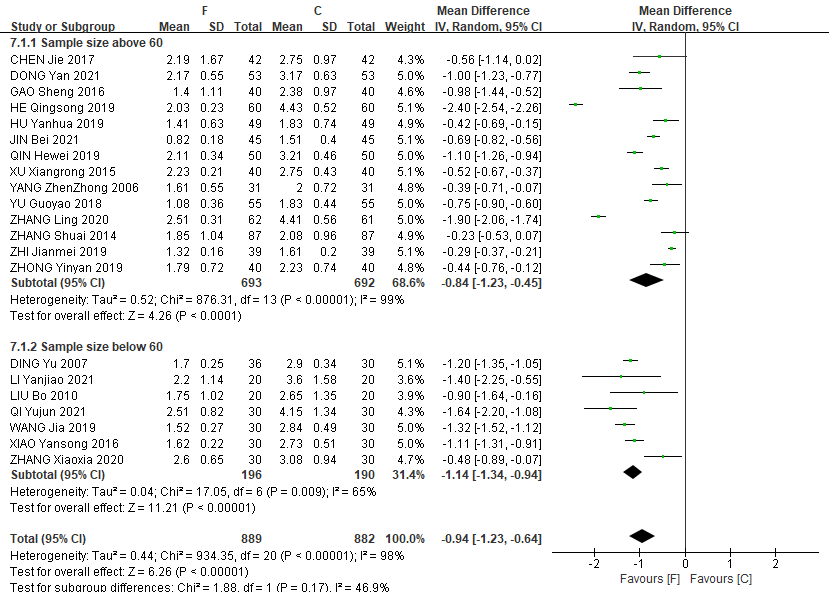


Appendix 4-figure 35 Subgroup’ s forest plot of WST of C compared with F

Legend of the figure is same as Appendix 4-figure 1.

**C compared with G**

(1) VFSS: 8 studies reported VFSS, including 522 patients, 261 in group G and 261 in Group C. High heterogeneity among studies (P <0.00001, I^2^=99%), and a random effects model analysis was used. The results showed that G had better in improving VFSS than C, and the difference was statistically significant [MD=1.83, 95%CI(-0.09, 3.74), P=0.06].


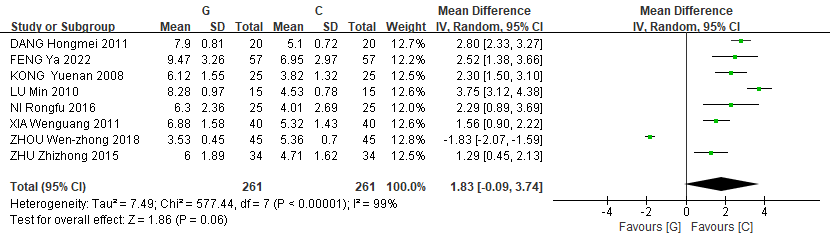
Appendix 4-figure 36 Forest plot of VFSS of C compared with G

Legend of the figure is same as Appendix 4-figure 1.

Read the original article found the study ZHOU Wen-zhong2018 its graphical results contradicted the conclusions and were excluded. To explore further its source of heterogeneity, subgroup analysis was performed based on the difference in sample sizes. The results showed that different sample sizes may be the reason for the heterogeneity among the included studies in the VFSS. ① Four studies with a sample size >60 were included, and the heterogeneity among studies was large (P=0.02, I^2^=70%). The results showed that G improved VFSS better than C, and the difference was statistically significant [MD=2.87, 95% CI (2.20,3.55), P<0.00001]. ② Three studies with a sample size <60 were included, and heterogeneity among studies was reduced (P=0.22, I^2^=33%). The results showed that G improved VFSS better than C, and the difference was statistically significant [MD=1.67, 95% CI (1.08,2.27), P<0.00001].


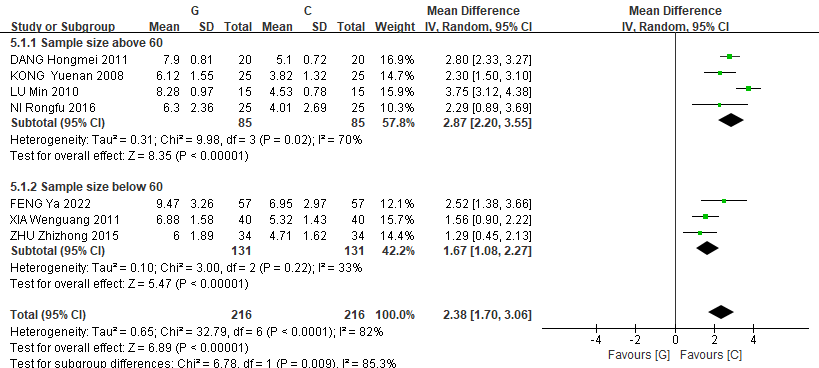


Appendix 4-figure 37 Subgroup’ s forest plot of VFSS of C compared with G

Legend of the figure is same as Appendix 4-figure 1.

(2) SSA: 6 studies reported a total of 458 patients in SSA, 230 in group G and 228 in group C.

High heterogeneity across studies (P <0.00001, I^2^=95%) was analyzed using a random effect model. The results showed that G reduced SSA better than C, with statistically significant differences [MD= -3.84, 95%CI (-6.97, -0.71), P=0.02].

To explore the source of heterogeneity, subgroup analyses were performed based on differences in disease duration. The results showed that differences in disease duration may account for the heterogeneity among the included studies of SSA. ① Three studies with disease duration >3 weeks were included, and the heterogeneity among studies was reduced (P=0.11, I^2^=55%). The results showed that G reduced SSA better than C, and the difference was statistically significant [MD=-5.06, 95% CI (-6.55,-3.57), P<0.00001]. ② Three studies with a disease duration of less than 3 weeks were included, and heterogeneity among studies was large (P<0.00001, I^2^=98%). The results showed that the difference in SSA lowering effect between the two groups was not statistically significant [MD=-2.94, 95% CI (-9.78,3.91), P=0.40].


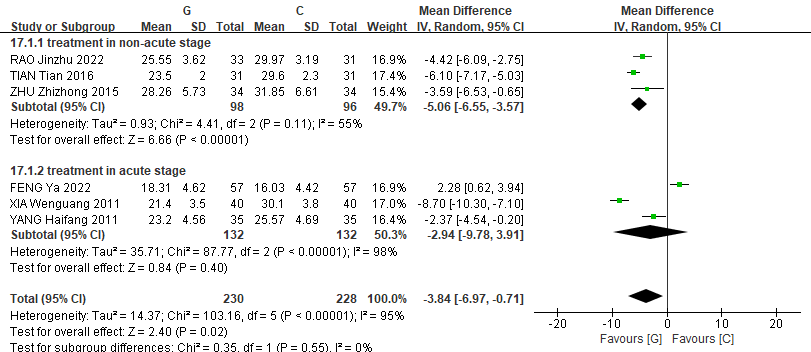


Appendix 4-figure 38 Forest plot of SSA of C compared with G

Legend of the figure is same as Appendix 4-figure 1.

(3) SWAL: Four studies reported a total of 266 patients enrolled in SWAL, 133 in group G and 133 in Group C.

High heterogeneity across studies (P <0.00001, I^2^=99%), which was analyzed using a random effect model. The results showed that there was no significant difference in the improvement of SWAL between the two groups [MD= -64.34, 95%CI (-150.50,21.83), P=0.14].

To explore the source of heterogeneity, subgroup analyses were performed based on disease duration. The results showed that disease duration was not the cause of heterogeneity among the included studies of SWAL, and the source of heterogeneity was unknown. ① Two studies with a disease duration of <3 weeks were included, and no significant reduction in heterogeneity was seen between studies (P<0.00001, I^2^=96%). The results showed that at a disease duration of <3 weeks, C improved SWAL better than G, and the difference was statistically significant [MD=-139.80, 95% CI (-190.32,-89.28), P<0.00001]. ② Two studies with a disease duration of >3 weeks were included, and the heterogeneity between studies was not significantly reduced (P<0.00001, I^2^=91%). The results showed that the difference between the two groups in improving the effect of SWAL was not statistically significant [MD=11.61, 95% CI (-18.34,41.56), P=0.45].


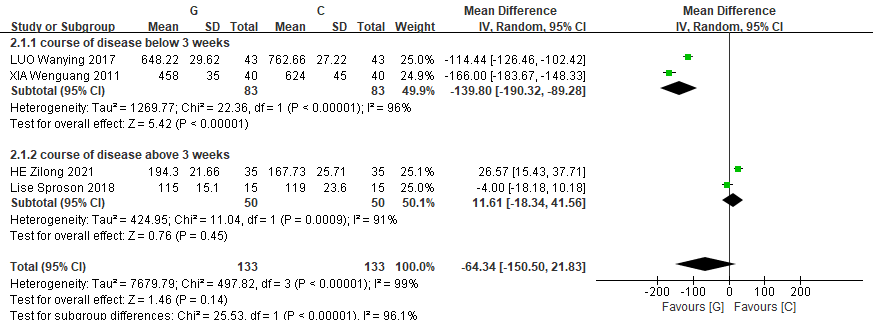


Appendix 4-figure 39 Forest plot of SWAL of C compared with G

Legend of the figure is same as Appendix 4-figure 1.

(4) WST: Two studies reported WST and included 202 patients, 101 in group G and 101 in group C. The homogeneity (P=0.89, I^2^=0%) was great among the studies and analyzed by fixed effect model. The results showed that G had better reduced WST than C with a statistically significant difference [MD=-0.74, 95%CI(-0.88,-0.59) ,P＜0.00001].


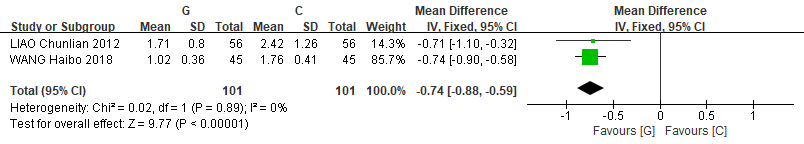


Appendix 4-figure 40 Forest plot of WST of C compared with G

Legend of the figure is same as Appendix 4-figure 1.

**C compared with H**

(1) VFSS: 4 studies reported VFSS, including 270 patients, 135 in group G and 135 in Group C. High heterogeneity among studies (P<0.0001, I^2^=87%), using random effects model analysis. The results showed that H had better in improving VFSS than C. The difference was statistically significant [MD=2.64, 95%CI(1.97,3.31), P＜0.00001].


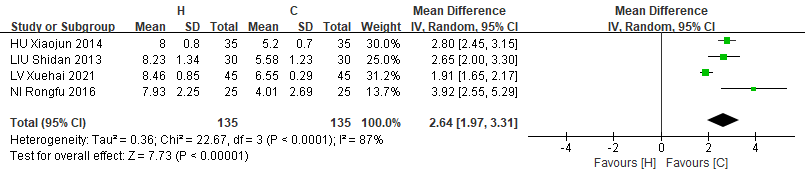
 Appendix 4-figure 41 Forest plot of VFSS of C compared with H

Legend of the figure is same as Appendix 4-figure 1.

(2) SSA: 2 studies reported a total of 150 patients enrolled in SSA, 75 in Group H and 75 in Group C. High heterogeneity across studies (P <0.0001, I^2^=94%), which was analyzed using a random effects model. The results showed that the effect of reducing SSA in H was better than C, which was statistically significant [MD=-3.74, 95%CI(-6.86,-0.63), P=0.02].


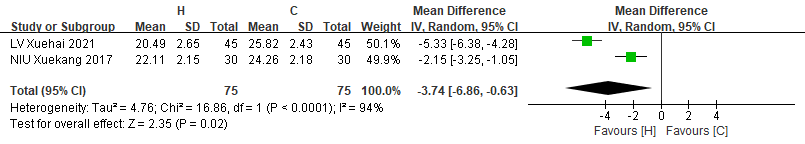
 Appendix 4-figure 42 Forest plot of SSA of C compared with H

Legend of the figure is same as Appendix 4-figure 1.

(3) SWAL: The results of one qualitative analysis of RCT showed that H improved SWAL better than C, and the difference was statistically significant [MD=30.00, 95%CI(26.81,33.19), P＜0.00001].


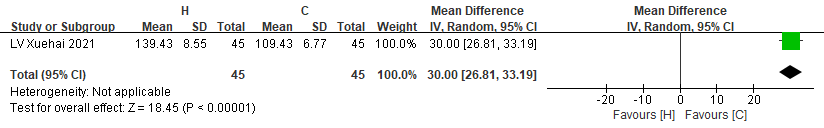


Appendix 4-figure 43 Forest plot of SWAL of C compared with H

Legend of the figure is same as Appendix 4-figure 1.

(4) WST: There was no direct comparison.

**C compared with I**

(1) VFSS: There was no direct comparison.

(2) SSA: There was no direct comparison.

(3) SWAL: The results of one qualitative analysis of RCT showed that I was better at improving SWAL than C, which was statistically significant [MD=25.59, 95%CI(14.80,36.38), P＜0.00001].


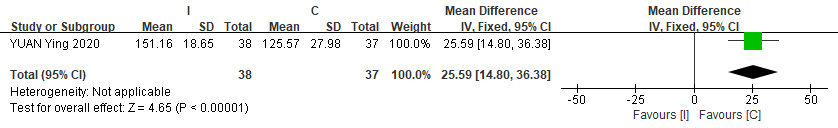


Appendix 4-figure 44 Forest plot of SWAL of C compared with I

Legend of the figure is same as Appendix 4-figure 1.

(4) WST: There was no direct comparison.

**C compared with J**

(1) VFSS: There was no direct comparison.

(2) SSA: There was no direct comparison.

(3) SWAL: There was no direct comparison.

(4) WST: There was no direct comparison.

**C compared with K**

(1) VFSS: The results of qualitative RCT showed that the K had a better effect in improving VFSS than the C, and the difference was statistically significant [MD=1.48, 95%CI(0.80, 2.16), P＜0.0001].


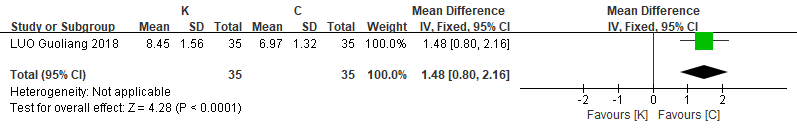
 Appendix 4-figure 45 Forest plot of VFSS of C compared with K

Legend of the figure is same as Appendix 4-figure 1.

(2) SSA: The results of one RCT showed that K had a better effect in reducing SSA than C, which was statistically significant [MD=-5.63, 95%CI(-7.19,-4.07), P＜0.00001].


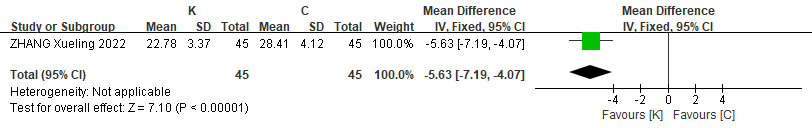


Appendix 4-figure 46 Forest plot of SSA of C compared with K

Legend of the figure is same as Appendix 4-figure 1.

(3) SWAL: The results of one qualitative RCT analysis showed that K improved SWAL better than C, and the difference was statistically significant [MD=17.57, 95%CI(4.96,30.18), P=0.006].


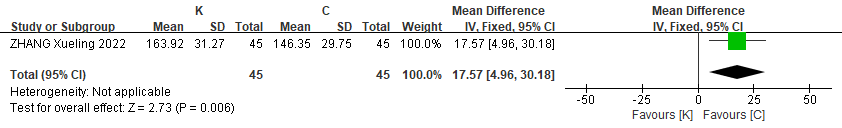


Appendix 4-figure 47 Forest plot of SWAL of C compared with K

Legend of the figure is same as Appendix 4-figure 1.

(4) WST: There was no direct comparison.

**C compared with L**

(1) VFSS: There was no direct comparison.

(2) SSA: The results of one qualitative analysis of RCT showed that L was better in reducing SSA than C, and the difference was statistically significant [MD=-1.92, 95%CI(-3.17,-0.67), P=0.003].

**
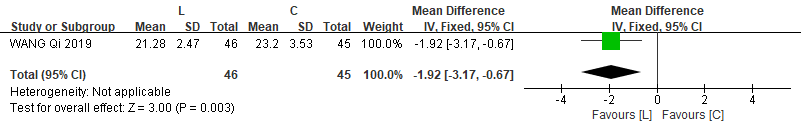
**

Appendix 4-figure 48 Forest plot of SSA of C compared with L

Legend of the figure is same as Appendix 4-figure 1.

(3) SWAL: 3 studies reported a total of 237 patients enrolled in SWAL, 119 in group L and 118 in group C. High heterogeneity across studies (P <0.0001, I^2^=90%), which was analyzed using a random effects model. The results showed that L improved SWAL better than C, with a statistically significant difference [MD=24.17, 95%CI(8.37,39.97), P=0.003].


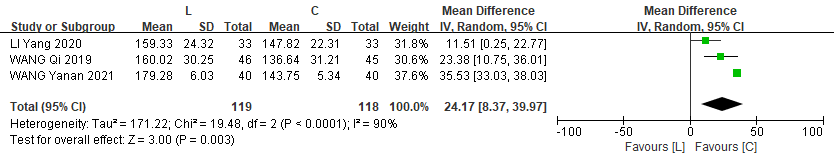


Appendix 4-figure 49 Forest plot of SWAL of C compared with L

Legend of the figure is same as Appendix 4-figure 1.

To explore the source of heterogeneity, subgroup analyses were performed based on duration of treatment. The results showed that duration of treatment may be the reason for the heterogeneity among the included studies of SWAL. ① Two studies with a duration of treatment below 2 weeks were included, and the heterogeneity among the studies decreased (P=0.17, I^2^=47%). The results showed that L increased SWAL better than C at a duration of treatment below 2 weeks, and the difference was statistically significant [MD=16.77, 95%CI (8.36,25.17), P<0.0001]. ② One study with a duration of treatment above 2 weeks was included, and the results of the qualitative analysis of the RCT showed that L increased SWAL better than C, and the difference was statistically significant [MD= 35.53, 95%CI(31.62,36.40), p<0.0001].

**
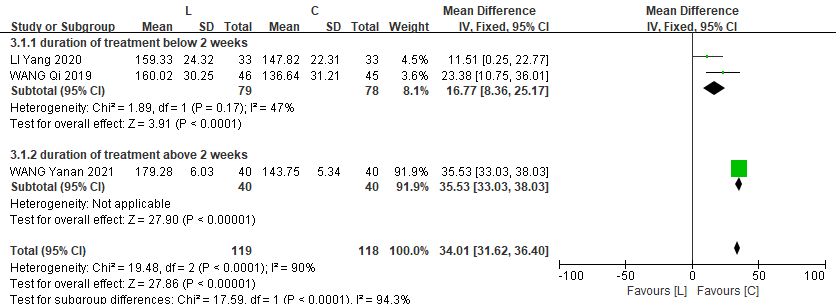
**

Appendix 4-figure 50 Subgroup’ s forest plot of SWAL of C compared with L

Legend of the figure is same as Appendix 4-figure 1.

(4) WST: 3 studies reported WST,and a total of 237 patients were enrolled,119 in group L and 118 in group C. The results of these studies were summarized as follows. Heterogeneity among the studies was large (P<0.00001, I^2^=99%) and was analyzed using a random-effects model. The results showed that L reduced WST better than C, and the difference was statistically significant [MD=-3.58, 95%CI(-6.77,-0.40), P=0.03].


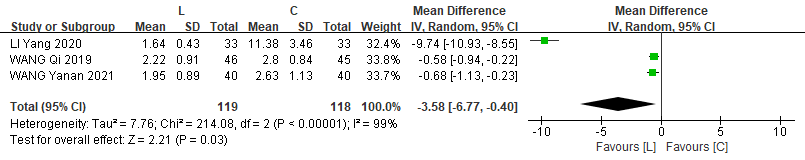


Appendix 4-figure 51 Forest plot of WST of C compared with L

Legend of the figure is same as Appendix 4-figure 1.

**D compared with E**

(1) VFSS: There was no direct comparison.

(2) SSA: There was no direct comparison.

(3) SWAL: There was no direct comparison.

(4) WST: The results of one qualitative RCT analysis and RCT showed that E was better in reducing WST than D, and the difference was statistically significant [MD=-0.74, 95%CI(-0.84,-0.64), P＜0.00001].


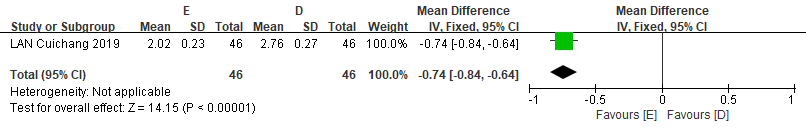


Appendix 4-figure 52 Forest plot of WST of D compared with E

Legend of the figure is same as Appendix 4-figure 1.

**D compared with F**

(1) VFSS: There was no direct comparison.

(2) SSA: There was no direct comparison.

(3) SWAL: There was no direct comparison.

(4) WST: The results of one qualitative analysis of RCT showed that F had a better effect in reducing WST than D, with a significant difference [MD=-0.94, 95%CI(-1.15,-0.73), P＜0.00001].


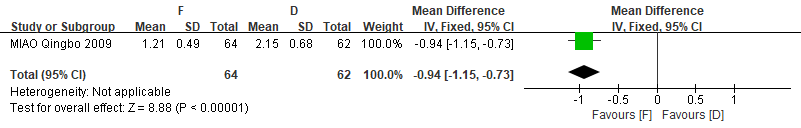


Appendix 4-figure 53 Forest plot of WST of D compared with F

Legend of the figure is same as Appendix 4-figure 1.

**D compared with G**

(1) VFSS: There was no direct comparison.

(2) SSA: There was no direct comparison.

(3) SWAL: There was no direct comparison.

(4) WST: There was no direct comparison.

**D compared with H**

(1) VFSS: 2 studies reported on VFSS, including 185 patients, 95 patients in group H and 90 patients in group D. Study homogeneity was great (P=0.20, I^2^=40%), and a fixed-effects model analysis was used. The results showed that H was better in improving VFSS than D, and the difference was statistically significant [MD=2.98, 95%CI(2.58,3.38), P＜0.00001].


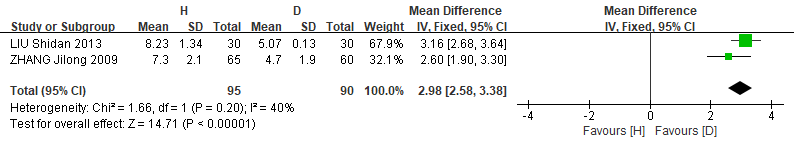


Appendix 4-figure 54 Forest plot of VFSS of D compared with H

Legend of the figure is same as Appendix 4-figure 1.

(2) SSA: There was no direct comparison.

(3) SWAL: There was no direct comparison.

(4) WST: There was no direct comparison.

**D compared with I**

None of them were directly comparable.

**D compared with J**

None of them were directly comparable.

**D compared with K**

None of them were directly comparable.

**D compared with L**

None of them were directly comparable.

**E compared with F**

None of them were directly comparable.

**E compared with G**

None of them were directly comparable.

**E compared with H**

None of them were directly comparable.

**E compared with I**

None of them were directly comparable.

**E compared with J**

None of them were directly comparable.

**E compared with K**

None of them were directly comparable.

**E compared with L**

None of them were directly comparable.

**F compared with G**

(1) VFSS: 6 studies reported VFSS and included 324 patients, 162 in group G and 162 in Group F. High heterogeneity across studies (P<0.00001, I^2^=90%) was analyzed using a random effect model. The results showed no significant difference between the two groups, [MD=0.10,95%CI(-0.83,1.04),P=0.83]. This indicated that there was no significant difference in the efficacy between F and G on VFSS.


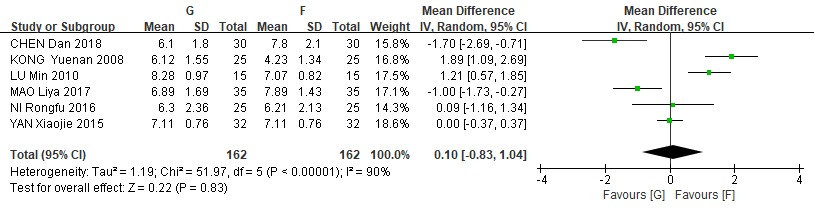


Appendix 4-figure 55 Forest plot of VFSS of F compared with G

Legend of the figure is same as Appendix 4-figure 1.

(2) SSA: The results of one qualitative analysis of RCT showed that there was no significant difference between the two groups [MD= -2.35, 95%CI (-5.01,0.31), P=0.08], indicating that there was no significant difference in the efficacy of SSA between F and C.


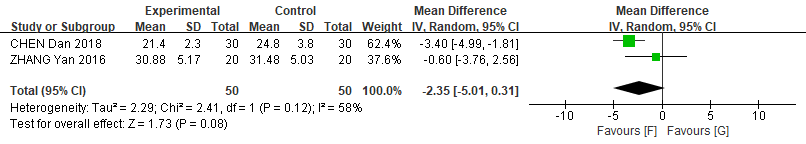


Appendix 4-figure 56 Forest plot of SSA of F compared with G

Legend of the figure is same as Appendix 4-figure 1.

(3) SWAL: There was no direct comparison.

(4) WST: 2 studies reported on WST, including 102 patients, 51 in Group G and 51 in Group F. High heterogeneity across studies (P<0.00001, I^2^=91%) was analyzed using a random effect model. The results showed no statistically significant difference between the two groups [MD= -0.62, 95%CI (-1.52,0.28), P=0.18], indicating that there was no significant difference in the efficacy of WST between F and .


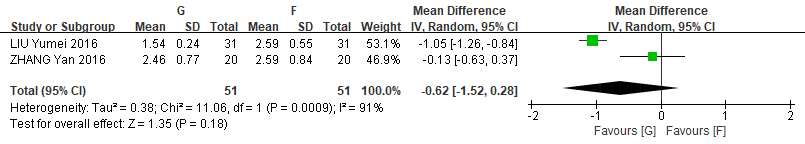


Appendix 4-figure 57 Forest plot of WST of F compared with G

Legend of the figure is same as Appendix 4-figure 1.

**F compared with H**

(1) VFSS: The results of one qualitative RCT analysis showed that H had a better effect in improving VFSS than F, and there was a statistically significant difference [MD=1.72, 95%CI(0.51,2.93), P=0.006].


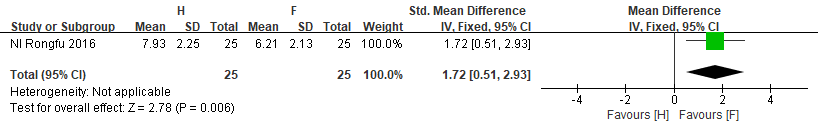
 Appendix 4-figure 58 Forest plot of VFSS of F compared with H

Legend of the figure is same as Appendix 4-figure 1.

(2) SSA: 3 studies reported on SSA, including 250 patients, 125 in Group H and 125 in Group F. Study homogeneity was great (P=0.31, I^2^=15%), using a fixed-effects model. The results showed that A decreased SSA better than C, and the difference was statistically significant [MD=-6.27, 95%CI(-7.31,-5.23), P＜0.00001].


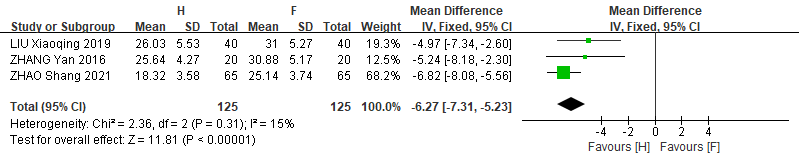


Appendix 4-figure 59 Forest plot of SSA of F compared with H

Legend of the figure is same as Appendix 4-figure 1.

(3) SWAL: 2 studies reported a total of 210 patients enrolled in SWAL, 105 in Group H and 105 in Group F. Great interstudy homogeneity (P=0.38, I^2^=0%) was analyzed by a fixed-effects model. The results showed that H improved improved SWAL than F, and the difference was statistically significant [MD=26.27, 95%CI(22.90,29.65), P<0.00001].
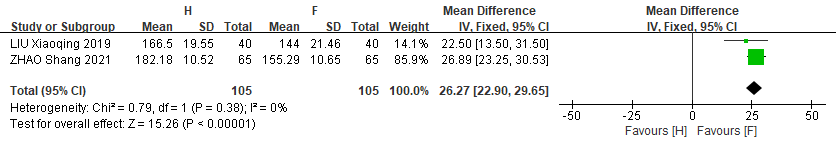


Appendix 4-figure 60 Forest plot of SWAL of F compared with H

Legend of the figure is same as Appendix 4-figure 1.

(4) WST: 2 studies reported on WST, including 102 patients, 51 in Group G and 51 in Group F. High heterogeneity across studies (P<0.00001, I^2^=94%) was analyzed using a random effect model. The results showed no significant difference between the two groups [MD=0.64, 95%CI (-0.49,1.77), P=0.26], indicating that there was no significant difference in WST efficacy between F and H.


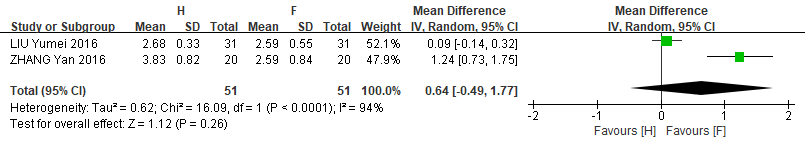


Appendix 4-figure 61 Forest plot of WST of F compared with H

Legend of the figure is same as Appendix 4-figure 1.

**F compared with I**

None of them were directly comparable.

**F compared with J**

(1) VFSS: There was no direct comparison.

(2) SSA: The results of one RCT showed that J reduced more SSA than F, with a statistically significant difference [MD=-2.89, 95%CI(-5.17,-0.61), P=0.01].


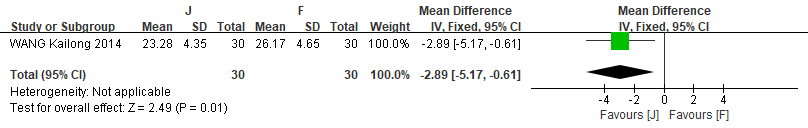


Appendix 4-figure 62 Forest plot of SSA of F compared with J

Legend of the figure is same as Appendix 4-figure 1.

(3) SWAL: There was no direct comparison.

(4) WST: There was no direct comparison.

**F compared with K**

None of them were directly comparable.

**F compared with L**

None of them were directly comparable.

**G compared with H**

(1) VFSS: 3 studies reported on VFSS, including 181 patients, 91 in Group H and 91 in Group G. Study homogeneity was great (P=0.13, I^2^=50%), using a fixed-effect model. The results showed that G was better in improving VFSS than H, and the difference was statistically significant [MD=2.12, 95%CI(1.77,2.47), P＜0.00001].


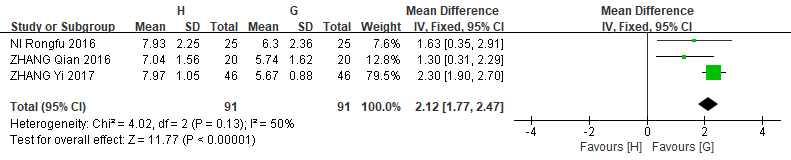


Appendix 4-figure 63 Forest plot of VFSS of G compared with H

Legend of the figure is same as Appendix 4-figure 1.

(2) SSA: 9 studies reported a total of 224 patients enrolled in SSA, 112 in Group H and 112 in Group G. High heterogeneity across studies (P<0.00001, I^2^=98%) was analyzed using a random effect model. The results showed no statistically significant difference between the two groups [MD= -1.14, 95%CI (-8.26,5.98), P=0.75], indicating that there was no significant difference in the efficacy of SSA between H and G.


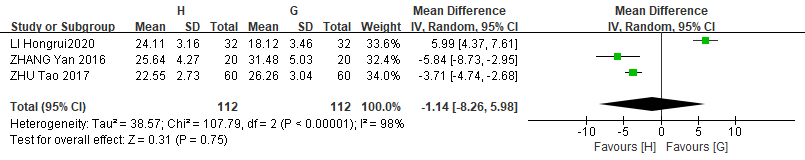


Appendix 4-figure 64 Forest plot of SSA of G compared with H

Legend of the figure is same as Appendix 4-figure 1.

(3) SWAL: 2 studies reported a total of 138 patients enrolled in SWAL, 69 in group H and 69 in Group G. High heterogeneity across studies (P=0.02, I^2^=82%) was analyzed using a random effect model. The results showed that H improved SWAL better than G, and the difference was statistically significant [MD=34.90, 95%CI(18.28,51.51), P＜0.0001].


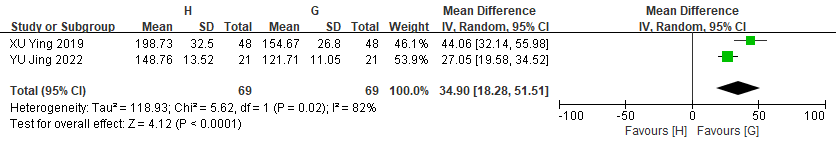


Appendix 4-figure 65 Forest plot of SWAL of G compared with H

Legend of the figure is same as Appendix 4-figure 1.

(4) WST: 4 studies reported on WST and included 258 patients, 129 in group H and 129 in Group G. High heterogeneity across studies (P<0.00001, I^2^=99%), which was analyzed using a random effect model. The results showed no statistically significant difference between the two groups [MD=0.11, 95%CI (-1.17,1.39), P=0.87], indicating that there was no significant difference in WST efficacy between H and G.


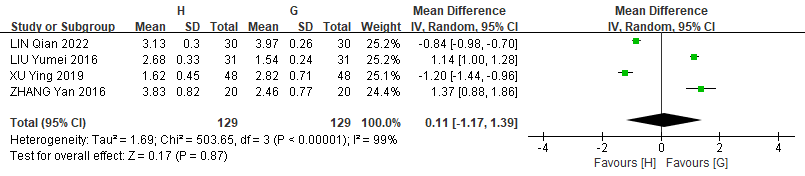


Appendix 4-figure 66 Forest plot of WST of G compared with H

Legend of the figure is same as Appendix 4-figure 1.

**G compared with I**

None of them were directly comparable.

**G compared with J**

None of them were directly comparable.

**G compared with K**

None of them were directly comparable.

**G compared with L**

None of them were directly comparable.

**H compared with I**

None of them were directly comparable.

**H compared with J**

None of them were directly comparable.

**H compared with K**

None of them were directly comparable.

**H compared with L**

None of them were directly comparable.

**I compared with J**

None of them were directly comparable.

**I compared with K**

None of them were directly comparable.

**I compared with L**

None of them were directly comparable.

**J compared with K**

None of them were directly comparable.

**J compared with L**

None of them were directly comparable.

**K compared with L**

None of them were directly comparable.
