# Supplementary material for: Comparison of efficacy of non-pharmacological intervention for post-stroke dysphagia: a systematic review and Bayesian network meta-analysis
Source: BMC Neurosci. 2023 Oct 16;24:53. doi: 10.1186/s12868-023-00825-0 (PMC10578008; doi:10.1186/s12868-023-00825-0)
Supplement: Supplementary file 5 — Additional file 5. Consistency analysis and subgroup analysis. [file 12868_2023_825_MOESM5_ESM.docx]

**Appendix 5- consistency analysis and subgroup analysis**

**Results of consistency analysis**

1.VFSS

**
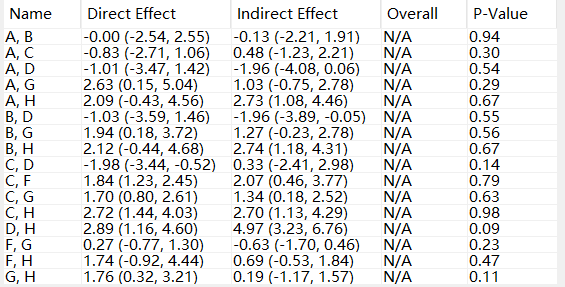
**

Appendix 5-figure 1 Results of consistency analysis of VFSS

The letters in the figure represent the 12 non-pharmacological interventions. A means acupuncture; B means electrotherapy; C means rehabilitation training; D means conventional treatment; E means acupuncture+electrotherapy; F means acupuncture+rehabilitation training; G means electrotherapy+rehabilitation training; H means acupuncture+electrotherapy+rehabilitation training; I means acupoints sticking; J means acupuncture+rehabilitation training+massage; K means rehabilitation training+acupoints sticking; L means acupuncture+rehabilitation training+acupoints sticking. The following figures and tables are the same.

P-Value shows the results of consistency analysis. If P > 0.05, it indicates consistency; otherwise, it is inconsistent.

2.SSA

**
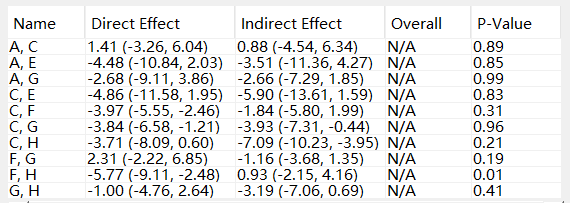
**

Appendix 5-figure 2 Results of consistency analysis of SSA

Legend of the figure is same as Appendix 5-figure 1.

3.SWAL

**
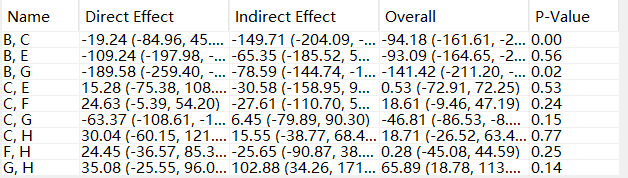
**

Appendix 5-figure 3 Results of consistency analysis of SWAL

Legend of the figure is same as Appendix 5-figure 1.

4.WST

**
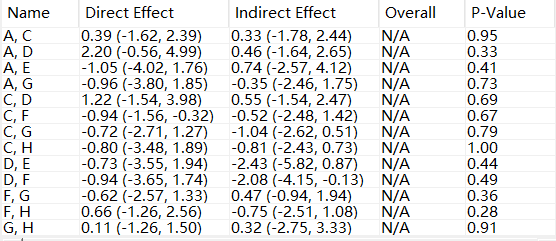
**

Appendix 5-figure 4 Results of consistency analysis of WST

Legend of the figure is same as Appendix 5-figure 1.

**Subgroup analysis of SSA**

1. Average age

According to the different average age, the subgroup analysis showed that when the average age was less than 60 years old, the probability ranking was H (60%) > G (25%) >F (8%) > A (7%). Suggesting that H was the most lively to be the best intervention. Node-splitting analysis showed that the P-Value of consistency analysis was greater than 0.05, and the consistency was good. When the average age was over 60 years old, there were local inconsistencies between C and H, F and H according to node-splittings.

1. 1 Average age ≤60 years old

Appendix 5-table 1 Network meta-analysis of SSA by average age ≤60 years old

| A | 2.00 (-4.42, 8.40) | -1.03 (-8.17, 5.93) | -3.20 (-9.40, 3.05) | -4.12 (-11.56, 3.37) |
| --- | --- | --- | --- | --- |
| -2.00 (-8.40, 4.42) | C | -3.07 (-6.72, 0.64) | -5.19 (-10.46, 0.03) | -6.12 (-12.65, 0.24) |
| 1.03 (-5.93, 8.17) | 3.07 (-0.64, 6.72) | F | -2.16 (-7.59, 3.16) | -3.12 (-9.42, 3.16) |
| 3.20 (-3.05, 9.40) | 5.19 (-0.03, 10.46) | 2.16 (-3.16, 7.59) | G | -0.96 (-5.63, 3.53) |
| 4.12 (-3.37, 11.56) | 6.12 (-0.24, 12.65) | 3.12 (-3.16, 9.42) | 0.96 (-3.53, 5.63) | H |

A means acupuncture; C means rehabilitation training; F means acupuncture+rehabilitation training; G means electrotherapy+rehabilitation training; H means acupuncture+electrotherapy+rehabilitation training; sticking. The following are the same.


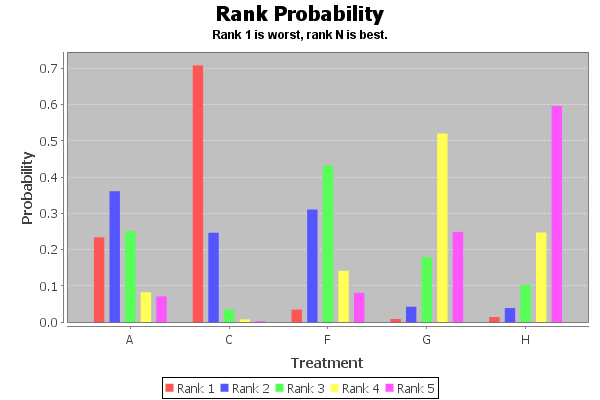


Appendix 5-figure 5 Rank probability of SSA by average age ≤60 years old

Legend of the figure is same as Appendix 5-table 1.

Appendix 5-figure 6 Details of rank probability of SSA by average age ≤60 years old

Legend of the figure is same as Appendix 5-table 1.

Appendix 5-figure 7 Results of consistency analysis of SSA by average age ≤60 years old

Legend of the figure is same as Appendix 5-figure 1.

1.2 Average age > 60 years old


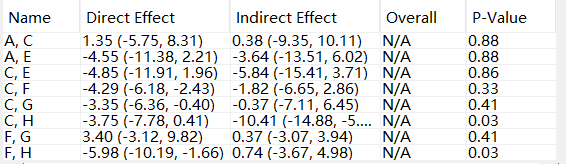


Appendix 5-figure 8 Results of consistency analysis of SSA by average age > 60 years old

Legend of the figure is same as Appendix 5-figure 1.

2. Course of treatment

Subgroup analysis was carried out according to the different course of treatment. The results showed that there was local inconsistency between C and H when the course of treatment was less than 4 weeks. After the course of treatment was more than 4 weeks, there was local

inconsistency between C and H, G and H according to node-splittings.

2.1 Course of treatment ≤4 weeks.


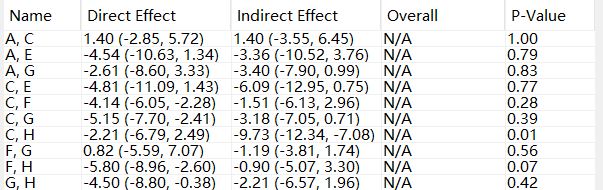


Appendix 5-figure 9 Results of consistency analysis of SSA by course of treatment ≤4 weeks

Legend of the figure is same as Appendix 5-figure 1.

2.2 Course of treatment > 4 weeks


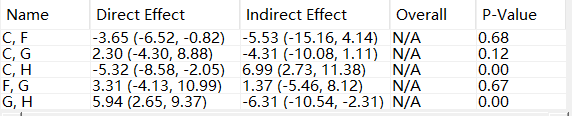


Appendix 5-figure 10 Results of consistency analysis of SSA by course of treatment > 4 weeks

Legend of the figure is same as Appendix 5-figure 1.

3. Sample size

According to the different sample size, the subgroup analysis showed that there was local inconsistency between F and H when the sample size was less than 80 cases. When the sample size was more than 80 cases, the results showed that the probability ranking was H (51%) > E (26%) >K (20%) > l (2%), suggesting that H was the most likely to be the best intervention. The node-splitting analysis showed that the P-Value > 0.05, and the consistency was good.

3.1 The sample size < 80 cases

Appendix 5-figure 11 Results of consistency analysis of SSA by sample size < 80 cases

Legend of the figure is same as Appendix 5-figure 1.

3.2 Sample size ≥80 cases

Appendix 5-table 2 Network meta-analysis of SSA by sample size ≥80 cases

| A | 2.97 (-5.39, 11.47) | 0.84 (-6.17, 8.01) | 5.94 (-3.28, 15.54) | -4.56 (-10.79, 1.64) | -2.49 (-9.70, 4.80) | -2.69 (-8.79, 3.53) | -6.89 (-14.32, 0.58) | -4.83 (-14.11, 4.88) | -1.08 (-10.33, 8.32) |
| --- | --- | --- | --- | --- | --- | --- | --- | --- | --- |
| -2.97 (-11.47, 5.39) | B | -2.07 (-7.89, 3.57) | 3.05 (-5.29, 11.41) | -7.53 (-18.17, 3.00) | -5.43 (-11.46, 0.40) | -5.61 (-11.39, 0.02) | -9.84 (-16.15, -3.61) | -7.69 (-15.82, 0.84) | -4.02 (-12.43, 4.25) |
| -0.84 (-8.01, 6.17) | 2.07 (-3.57, 7.89) | C | 5.14 (-0.92, 11.35) | -5.47 (-15.08, 4.13) | -3.35 (-5.10, -1.58) | -3.55 (-7.29, 0.26) | -7.73 (-11.17, -4.37) | -5.61 (-11.71, 0.62) | -1.96 (-7.97, 4.14) |
| -5.94 (-15.54, 3.28) | -3.05 (-11.41, 5.29) | -5.14 (-11.35, 0.92) | D | -10.54 (-21.74, 0.64) | -8.49 (-14.96, -2.19) | -8.67 (-15.89, -1.48) | -12.87 (-19.89, -5.89) | -10.77 (-19.35, -1.91) | -7.06 (-15.70, 1.57) |
| 4.56 (-1.64, 10.79) | 7.53 (-3.00, 18.17) | 5.47 (-4.13, 15.08) | 10.54 (-0.64, 21.74) | E | 2.07 (-7.69, 11.74) | 1.86 (-6.86, 10.64) | -2.25 (-12.21, 7.56) | -0.17 (-11.31, 11.34) | 3.51 (-7.97, 14.63) |
| 2.49 (-4.80, 9.70) | 5.43 (-0.40, 11.46) | 3.35 (1.58, 5.10) | 8.49 (2.19, 14.96) | -2.07 (-11.74, 7.69) | F | -0.23 (-4.20, 3.84) | -4.37 (-7.72, -1.01) | -2.25 (-8.57, 4.22) | 1.39 (-4.88, 7.70) |
| 2.69 (-3.53, 8.79) | 5.61 (-0.02, 11.39) | 3.55 (-0.26, 7.29) | 8.67 (1.48, 15.89) | -1.86 (-10.64, 6.86) | 0.23 (-3.84, 4.20) | G | -4.20 (-8.28, -0.05) | -2.08 (-9.15, 5.14) | 1.61 (-5.48, 8.63) |
| 6.89 (-0.58, 14.32) | 9.84 (3.61, 16.15) | 7.73 (4.37, 11.17) | 12.87 (5.89, 19.89) | 2.25 (-7.56, 12.21) | 4.37 (1.01, 7.72) | 4.20 (0.05, 8.28) | H | 2.16 (-4.88, 9.13) | 5.79 (-1.06, 12.71) |
| 4.83 (-4.88, 14.11) | 7.69 (-0.84, 15.82) | 5.61 (-0.62, 11.71) | 10.77 (1.91, 19.35) | 0.17 (-11.34, 11.31) | 2.25 (-4.22, 8.57) | 2.08 (-5.14, 9.15) | -2.16 (-9.13, 4.88) | K | 3.69 (-5.15, 12.30) |
| 1.08 (-8.32, 10.33) | 4.02 (-4.25, 12.43) | 1.96 (-4.14, 7.97) | 7.06 (-1.57, 15.70) | -3.51 (-14.63, 7.97) | -1.39 (-7.70, 4.88) | -1.61 (-8.63, 5.48) | -5.79 (-12.71, 1.06) | -3.69 (-12.30, 5.15) | L |

A means acupuncture; B means electrotherapy; C means rehabilitation training; D means conventional treatment; E means acupuncture+electrotherapy; F means acupuncture+rehabilitation training; G means electrotherapy+rehabilitation training; H means acupuncture+electrotherapy+rehabilitation training; K means rehabilitation training+acupoints sticking; L means acupuncture+rehabilitation training+acupoints sticking. The following figures and tables are the same.


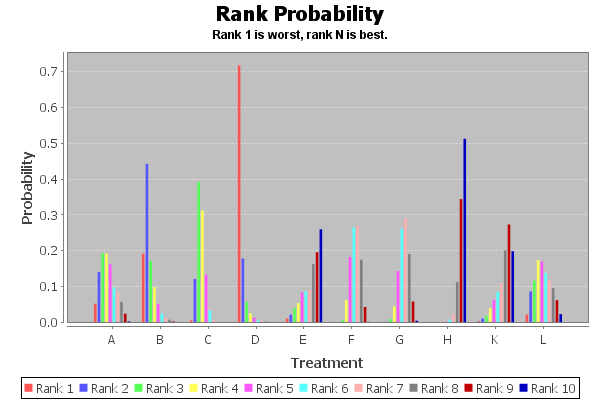


Appendix 5-figure 12 Rank probability of SSA by sample size ≥80 cases

Legend of the figure is same as Appendix 5-table 2.

Appendix 5-figure 13 Details of rank probability of SSA by sample size ≥80 cases

Legend of the figure is same as Appendix 5-table 2.


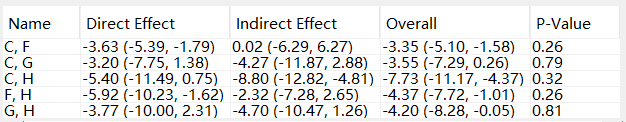


Appendix 5-figure 14 Results of consistency analysis of SSA by sample size ≥80 cases

Legend of the figure is same as Appendix 5-figure 1.

**Sensitivity analysis of SSA**

Subgroup analysis failed to find the source of inconsistency completely. Because many subgroup analyses showed that there was a high probability of inconsistency between F and H, sensitivity analysis of three studies excluding F-H was adopted. The results of sensitivity analysis showed that the probability ranking was J (49%) > K (26%) > E (19%) > l (3%). Suggesting that J was the most likely to be the best intervention. Node-splitting analysis showed that the P-Value > 0.05, and the consistency was good.

Appendix 5-table 3 Results of sensitivity analysis

| A | 3.19 (-2.83, 9.18) | 1.12 (-2.13, 4.29) | 6.20 (-0.51, 12.78) | -4.19 (-8.80, 0.50) | -3.00 (-6.46, 0.43) | -2.49 (-5.98, 0.98) | -2.05 (-6.30, 2.22) | -5.85 (-12.78, 1.21) | -4.47 (-11.05, 2.00) | -0.77 (-7.41, 5.78) |
| --- | --- | --- | --- | --- | --- | --- | --- | --- | --- | --- |
| -3.19 (-9.18, 2.83) | B | -2.06 (-7.24, 3.15) | 3.00 (-4.62, 10.82) | -7.38 (-14.16, -0.39) | -6.19 (-11.60, -0.92) | -5.69 (-10.88, -0.44) | -5.22 (-11.01, 0.60) | -9.05 (-17.01, -0.92) | -7.61 (-15.58, 0.11) | -3.98 (-11.58, 3.61) |
| -1.12 (-4.29, 2.13) | 2.06 (-3.15, 7.24) | C | 5.06 (-0.75, 10.92) | -5.28 (-9.93, -0.76) | -4.12 (-5.53, -2.74) | -3.61 (-5.63, -1.55) | -3.16 (-6.13, -0.15) | -6.97 (-13.13, -0.83) | -5.59 (-11.45, 0.16) | -1.92 (-7.68, 3.79) |
| -6.20 (-12.78, 0.51) | -3.00 (-10.82, 4.62) | -5.06 (-10.92, 0.75) | D | -10.40 (-17.56, -2.93) | -9.21 (-15.17, -3.17) | -8.68 (-14.80, -2.46) | -8.22 (-14.86, -1.76) | -12.02 (-20.33, -3.38) | -10.61 (-18.88, -2.64) | -6.95 (-15.29, 1.25) |
| 4.19 (-0.50, 8.80) | 7.38 (0.39, 14.16) | 5.28 (0.76, 9.93) | 10.40 (2.93, 17.56) | E | 1.17 (-3.68, 6.02) | 1.67 (-3.26, 6.57) | 2.07 (-3.28, 7.57) | -1.65 (-9.30, 6.00) | -0.28 (-7.84, 7.06) | 3.35 (-3.97, 10.81) |
| 3.00 (-0.43, 6.46) | 6.19 (0.92, 11.60) | 4.12 (2.74, 5.53) | 9.21 (3.17, 15.17) | -1.17 (-6.02, 3.68) | F | 0.50 (-1.86, 2.89) | 0.94 (-2.34, 4.25) | -2.83 (-8.75, 3.15) | -1.46 (-7.52, 4.54) | 2.23 (-3.69, 8.17) |
| 2.49 (-0.98, 5.98) | 5.69 (0.44, 10.88) | 3.61 (1.55, 5.63) | 8.68 (2.46, 14.80) | -1.67 (-6.57, 3.26) | -0.50 (-2.89, 1.86) | G | 0.43 (-2.61, 3.52) | -3.37 (-9.73, 3.08) | -1.95 (-8.16, 4.13) | 1.70 (-4.33, 7.78) |
| 2.05 (-2.22, 6.30) | 5.22 (-0.60, 11.01) | 3.16 (0.15, 6.13) | 8.22 (1.76, 14.86) | -2.07 (-7.57, 3.28) | -0.94 (-4.25, 2.34) | -0.43 (-3.52, 2.61) | H | -3.84 (-10.51, 2.97) | -2.40 (-8.99, 4.14) | 1.25 (-5.31, 7.79) |
| 5.85 (-1.21, 12.78) | 9.05 (0.92, 17.01) | 6.97 (0.83, 13.13) | 12.02 (3.38, 20.33) | 1.65 (-6.00, 9.30) | 2.83 (-3.15, 8.75) | 3.37 (-3.08, 9.73) | 3.84 (-2.97, 10.51) | J | 1.29 (-7.03, 9.68) | 5.05 (-3.30, 13.40) |
| 4.47 (-2.00, 11.05) | 7.61 (-0.11, 15.58) | 5.59 (-0.16, 11.45) | 10.61 (2.64, 18.88) | 0.28 (-7.06, 7.84) | 1.46 (-4.54, 7.52) | 1.95 (-4.13, 8.16) | 2.40 (-4.14, 8.99) | -1.29 (-9.68, 7.03) | K | 3.63 (-4.39, 11.97) |
| 0.77 (-5.78, 7.41) | 3.98 (-3.61, 11.58) | 1.92 (-3.79, 7.68) | 6.95 (-1.25, 15.29) | -3.35 (-10.81, 3.97) | -2.23 (-8.17, 3.69) | -1.70 (-7.78, 4.33) | -1.25 (-7.79, 5.31) | -5.05 (-13.40, 3.30) | -3.63 (-11.97, 4.39) | L |

A means acupuncture; B means electrotherapy; C means rehabilitation training; D means conventional treatment; E means acupuncture+electrotherapy; F means acupuncture+rehabilitation training; G means electrotherapy+rehabilitation training; H means acupuncture+electrotherapy+rehabilitation training; J means acupuncture+rehabilitation training+massage; K means rehabilitation training+acupoints sticking; L means acupuncture+rehabilitation training+acupoints sticking.

**
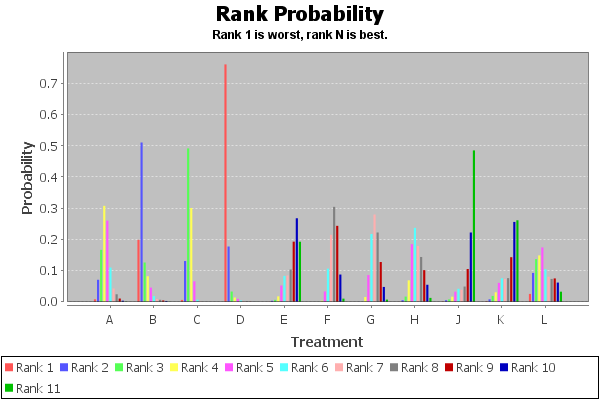
**

Appendix 5-figure 15 Rank probability of SSA

Legend of the figure is same as Appendix 5-table 3.

Appendix 5-figure 16 Details of rank probability of SSA

Legend of the figure is same as Appendix 5-table 3.

**
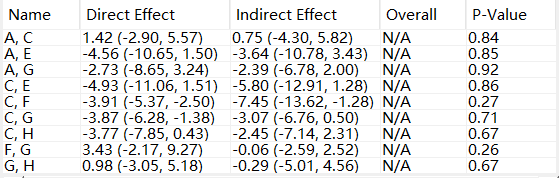
**

Appendix 5-figure 17 Results of consistency analysis of SSA

Legend of the figure is same as Appendix 5-figure 1.

**Subgroup analysis of SWAL**

1. Average age

According to the difference of average age, the subgroup analysis showed that when the average age was less than 65 years old, the probability ranking was H (54%) > G (17%) > I (15%). The node-splitting analysis showed that the P-Value > 0.05, and consistency was good. When the average age was over 65 years old, the results showed that the probability ranking was B (68%) > F (16%) > A (6%) > H (5%), suggesting that B was the most likely to be the best intervention. The node-splitting analysis showed that the P-Value > 0.05, and the consistency was good.

1. 1 Average age ≤65 years old

Appendix 5-table 4 Network meta-analysis of SWAL by average age ≤65 years old

| C | -11.66 (-37.37, 14.13) | 15.24 (-11.14, 40.72) | 17.05 (5.77, 28.44) | 26.41 (-0.40, 54.36) | 36.75 (17.56, 55.72) | 25.84 (-2.08, 52.32) | 17.35 (-10.23, 45.14) | 24.89 (8.10, 39.93) |
| --- | --- | --- | --- | --- | --- | --- | --- | --- |
| 11.66 (-14.13, 37.37) | D | 27.20 (-10.40, 62.61) | 28.59 (1.03, 57.01) | 38.24 (0.71, 75.48) | 48.59 (16.70, 80.37) | 37.69 (-0.36, 73.04) | 28.92 (-7.62, 66.30) | 36.46 (6.04, 65.87) |
| -15.24 (-40.72, 11.14) | -27.20 (-62.61, 10.40) | E | 1.78 (-25.66, 30.53) | 11.06 (-26.12, 49.02) | 21.71 (-10.68, 54.19) | 10.51 (-26.67, 47.94) | 2.11 (-36.11, 40.69) | 9.66 (-20.94, 40.38) |
| -17.05 (-28.44, -5.77) | -28.59 (-57.01, -1.03) | -1.78 (-30.53, 25.66) | F | 9.31 (-19.56, 39.01) | 19.84 (0.47, 38.17) | 8.89 (-21.85, 37.22) | 0.25 (-29.94, 30.32) | 7.83 (-12.68, 26.38) |
| -26.41 (-54.36, 0.40) | -38.24 (-75.48, -0.71) | -11.06 (-49.02, 26.12) | -9.31 (-39.01, 19.56) | G | 10.32 (-23.37, 43.06) | -0.57 (-39.69, 37.72) | -8.90 (-48.09, 30.24) | -1.50 (-34.59, 29.21) |
| -36.75 (-55.72, -17.56) | -48.59 (-80.37, -16.70) | -21.71 (-54.19, 10.68) | -19.84 (-38.17, -0.47) | -10.32 (-43.06, 23.37) | H | -10.92 (-44.69, 20.75) | -19.50 (-53.65, 13.74) | -11.92 (-37.04, 12.29) |
| -25.84 (-52.32, 2.08) | -37.69 (-73.04, 0.36) | -10.51 (-47.94, 26.67) | -8.89 (-37.22, 21.85) | 0.57 (-37.72, 39.69) | 10.92 (-20.75, 44.69) | I | -8.30 (-46.30, 29.99) | -0.94 (-32.96, 30.41) |
| -17.35 (-45.14, 10.23) | -28.92 (-66.30, 7.62) | -2.11 (-40.69, 36.11) | -0.25 (-30.32, 29.94) | 8.90 (-30.24, 48.09) | 19.50 (-13.74, 53.65) | 8.30 (-29.99, 46.30) | K | 7.51 (-25.13, 38.29) |
| -24.89 (-39.93, -8.10) | -36.46 (-65.87, -6.04) | -9.66 (-40.38, 20.94) | -7.83 (-26.38, 12.68) | 1.50 (-29.21, 34.59) | 11.92 (-12.29, 37.04) | 0.94 (-30.41, 32.96) | -7.51 (-38.29, 25.13) | L |

C means rehabilitation training; D means conventional treatment; E means acupuncture+electrotherapy; F means acupuncture+rehabilitation training; G means electrotherapy+rehabilitation training; H means acupuncture+electrotherapy+rehabilitation training; I means acupoints sticking; K means rehabilitation training+acupoints sticking; L means acupuncture+rehabilitation training+acupoints sticking.


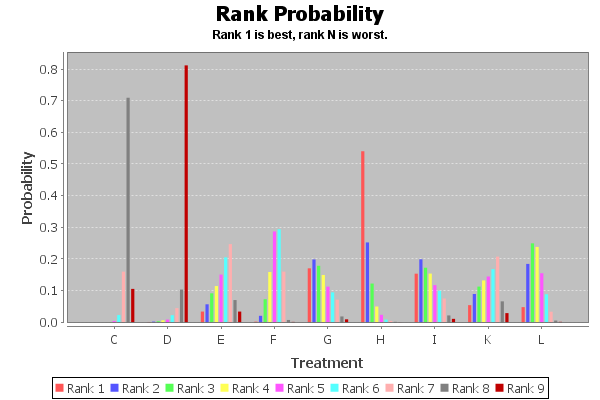


Appendix 5-figure 18 Rank probability of SWAL by average age ≤65 years old

Legend of the figure is same as Appendix 5-table 4.

Appendix 5-figure 19 Details of rank probability of SWAL by average age ≤65 years old

Legend of the figure is same as Appendix 5-table 4.


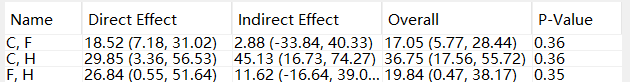


Appendix 5-figure 20 Results of consistency analysis of SWAL by average age ≤65 years old

Legend of the figure is same as Appendix 5-figure 1.

1.2 Average age > 65 years old
Appendix 5-table 5 Network meta-analysis of SWAL by average age >65 years old

| A | 133.90 (-68.28, 334.81) | 69.18 (-171.93, 303.06) | 24.31 (-120.77, 162.99) | 85.16 (-159.75, 329.09) | -9.10 (-245.94, 226.73) | 52.83 (-189.94, 294.77) |
| --- | --- | --- | --- | --- | --- | --- |
| -133.90 (-334.81, 68.28) | B | -66.12 (-190.19, 56.71) | -110.37 (-247.73, 28.71) | -48.81 (-191.33, 89.92) | -142.64 (-268.60, -21.27) | -81.51 (-224.60, 63.51) |
| -69.18 (-303.06, 171.93) | 66.12 (-56.71, 190.19) | C | -44.16 (-228.03, 144.44) | 17.08 (-48.94, 83.81) | -76.92 (-151.06, -2.15) | -14.39 (-111.95, 85.46) |
| -24.31 (-162.99, 120.77) | 110.37 (-28.71, 247.73) | 44.16 (-144.44, 228.03) | E | 61.12 (-139.84, 252.73) | -33.44 (-220.08, 148.30) | 30.08 (-170.11, 226.18) |
| -85.16 (-329.09, 159.75) | 48.81 (-89.92, 191.33) | -17.08 (-83.81, 48.94) | -61.12 (-252.73, 139.84) | F | -94.17 (-186.17, -3.89) | -32.14 (-133.97, 69.35) |
| 9.10 (-226.73, 245.94) | 142.64 (21.27, 268.60) | 76.92 (2.15, 151.06) | 33.44 (-148.30, 220.08) | 94.17 (3.89, 186.17) | G | 61.84 (-23.91, 147.68) |
| -52.83 (-294.77, 189.94) | 81.51 (-63.51, 224.60) | 14.39 (-85.46, 111.95) | -30.08 (-226.18, 170.11) | 32.14 (-69.35, 133.97) | -61.84 (-147.68, 23.91) | H |

A means acupuncture; B means electrotherapy; C means rehabilitation training; E means acupuncture+electrotherapy; F means acupuncture+rehabilitation training; G means electrotherapy+rehabilitation training; H means acupuncture+electrotherapy+rehabilitation training.


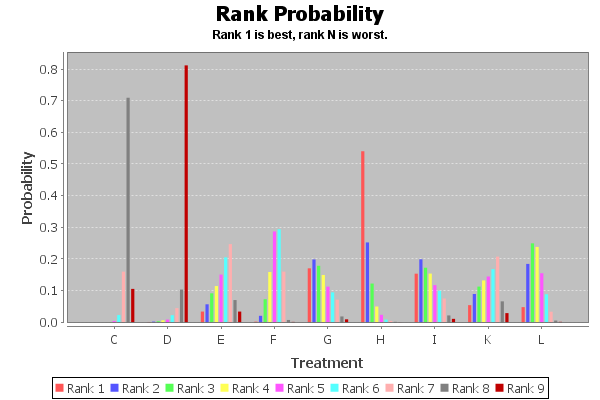


Appendix 5-figure 21 Rank probability of SWAL by average age > 65 years old

Legend of the figure is same as Appendix 5-table 5.

Appendix 5-figure 22 Details of rank probability of SWAL by average age >65 years old

Legend of the figure is same as Appendix 5-table 5.


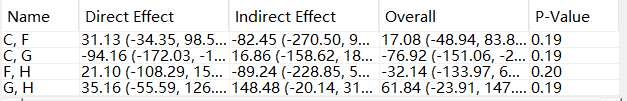


Appendix 5-figure 23 Results of consistency analysis of SWAL by average age >65 years old

Legend of the figure is same as Appendix 5-figure 1.

2. Course of treatment

Subgroup analysis was conducted according to the different courses of treatment. The results showed that there was local inconsistency between B and C when the course of treatment was less than 4 weeks. After a course of treatment of more than 4 weeks, the results showed that the probability ranking was H (73%) > K (12%) >F (11%) > G (2%), suggesting that H was the most likely to be the best intervention. Node-splitting analysis showed that the P-Value > 0.05, and the consistency was good.

2.1 Course of treatment ≤4 weeks.


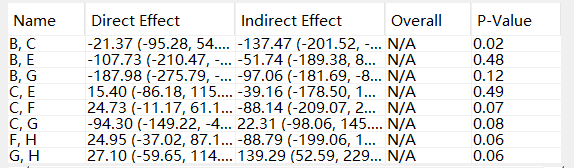


Appendix 5-figure 24 Results of consistency analysis of SWAL by course of treatment ≤4 weeks

Legend of the figure is same as Appendix 5-figure 1.

2.2 Course of treatment > 4 weeks

Appendix 5-table 6 Network meta-analysis of SWAL by course of treatment > 4 weeks

| C | -11.65 (-57.45, 33.27) | 23.78 (-2.87, 49.91) | 12.45 (-25.00, 52.10) | 41.92 (5.47, 80.68) | 17.94 (-28.89, 64.32) |
| --- | --- | --- | --- | --- | --- |
| 11.65 (-33.27, 57.45) | D | 35.28 (-14.72, 87.92) | 24.18 (-34.33, 84.63) | 53.56 (-4.37, 113.90) | 29.94 (-36.16, 94.36) |
| -23.78 (-49.91, 2.87) | -35.28 (-87.92, 14.72) | F | -11.12 (-57.54, 37.02) | 18.09 (-28.14, 66.09) | -5.43 (-59.65, 47.55) |
| -12.45 (-52.10, 25.00) | -24.18 (-84.63, 34.33) | 11.12 (-37.02, 57.54) | G | 29.53 (-8.53, 66.56) | 5.28 (-55.61, 62.97) |
| -41.92 (-80.68, -5.47) | -53.56 (-113.90, 4.37) | -18.09 (-66.09, 28.14) | -29.53 (-66.56, 8.53) | H | -24.22 (-84.07, 35.09) |
| -17.94 (-64.32, 28.89) | -29.94 (-94.36, 36.16) | 5.43 (-47.55, 59.65) | -5.28 (-62.97, 55.61) | 24.22 (-35.09, 84.07) | K |

C means rehabilitation training; D means conventional treatment; F means acupuncture+rehabilitation training; G means electrotherapy+rehabilitation training; H means acupuncture+electrotherapy+rehabilitation training; K means rehabilitation training+acupoints sticking.


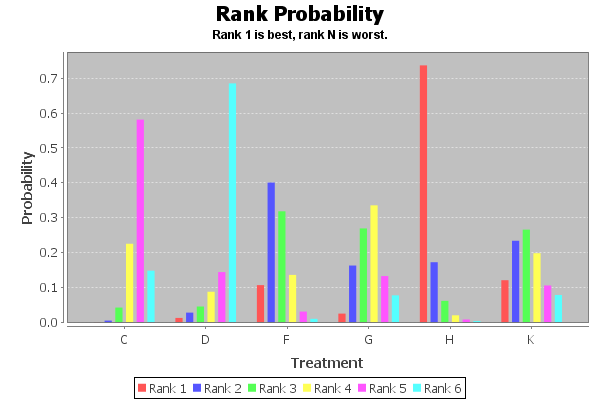


Appendix 5-figure 25 Rank probability of SWAL by course of treatment > 4 weeks

Legend of the figure is same as Appendix 5-table 6.

Appendix 5-figure 26 Details of rank probability of SWAL by course of treatment > 4 weeks

Legend of the figure is same as Appendix 5-table 6.


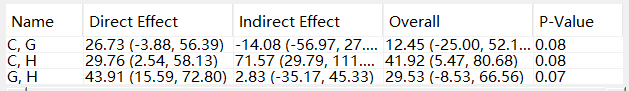


Appendix 5-figure 27 Results of consistency analysis of SWAL by course of treatment >4 weeks

Legend of the figure is same as Appendix 5-figure 1.

3. Sample size

According to the different sample sizes, the subgroup analysis showed that the probability ranking was H (49%) > F (14%) > E (8%) > l (7%), suggesting that H was the most likely to be the best intervention. Node-splitting analysis showed that the P-Value > 0.05, and the consistency was good. When the sample size was more than 80 cases, there was local inconsistency between C and G according to node-splittings.

3.1 Sample size < 80 cases
Appendix 5-table 7 Network meta-analysis of SWAL by sample size < 80 cases

| C | 15.39 (-31.44, 64.70) | 27.42 (-0.68, 56.92) | 11.88 (-24.11, 47.04) | 39.00 (-22.81, 99.92) | 25.38 (-22.82, 75.88) | 11.30 (-36.97, 60.77) |
| --- | --- | --- | --- | --- | --- | --- |
| -15.39 (-64.70, 31.44) | E | 12.01 (-44.63, 66.60) | -3.50 (-63.63, 56.86) | 23.86 (-53.36, 99.00) | 10.13 (-58.11, 79.25) | -3.85 (-71.66, 65.11) |
| -27.42 (-56.92, 0.68) | -12.01 (-66.60, 44.63) | F | -15.29 (-61.09, 28.93) | 11.60 (-55.65, 77.71) | -1.68 (-58.42, 55.55) | -15.87 (-71.32, 40.74) |
| -11.88 (-47.04, 24.11) | 3.50 (-56.86, 63.63) | 15.29 (-28.93, 61.09) | G | 27.07 (-22.88, 76.59) | 13.97 (-46.51, 75.03) | -0.43 (-59.98, 62.38) |
| -39.00 (-99.92, 22.81) | -23.86 (-99.00, 53.36) | -11.60 (-77.71, 55.65) | -27.07 (-76.59, 22.88) | H | -12.92 (-91.10, 65.84) | -27.45 (-104.03, 52.37) |
| -25.38 (-75.88, 22.82) | -10.13 (-79.25, 58.11) | 1.68 (-55.55, 58.42) | -13.97 (-75.03, 46.51) | 12.92 (-65.84, 91.10) | I | -14.44 (-82.42, 55.08) |
| -11.30 (-60.77, 36.97) | 3.85 (-65.11, 71.66) | 15.87 (-40.74, 71.32) | 0.43 (-62.38, 59.98) | 27.45 (-52.37, 104.03) | 14.44 (-55.08, 82.42) | L |

C means rehabilitation training; E means acupuncture+electrotherapy; F means acupuncture+rehabilitation training; G means electrotherapy+rehabilitation training; H means acupuncture+electrotherapy+rehabilitation training; I means acupoints sticking; L means acupuncture+rehabilitation training+acupoints sticking.


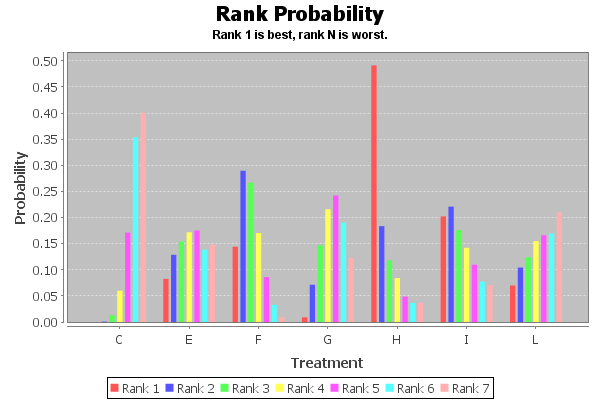


Appendix 5-figure 28 Rank probability of SWAL by sample size < 80 cases

Legend of the figure is same as Appendix 5-table 7.


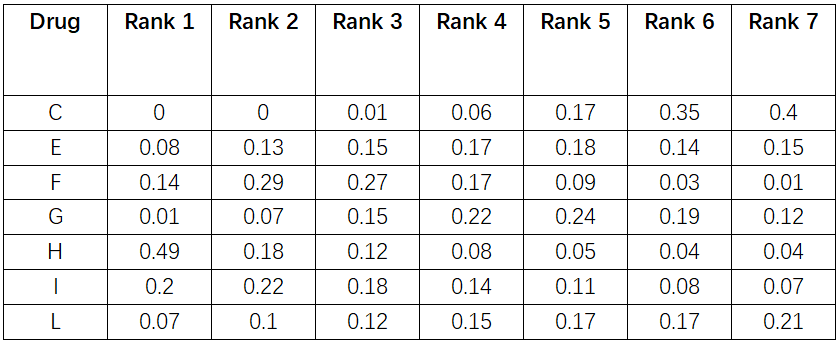


Appendix 5-figure 29 Details of rank probability of SWAL by sample size < 80 cases

Legend of the figure is same as Appendix 5-table 7.

3.2 Sample size ≥80 cases


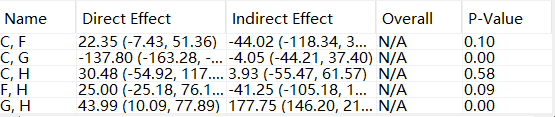


Appendix 5-figure 30 Results of consistency analysis of SWAL by sample size ≥80 cases

Legend of the figure is same as Appendix 5-figure 1.
